# Hi-GDT: A Hi-C-based 3D gene domain analysis tool for analyzing local chromatin contacts in plants

**DOI:** 10.1093/gigascience/giaf020

**Published:** 2025-03-21

**Authors:** Hongwoo Lee, Pil Joon Seo

**Affiliations:** Department of Chemistry, Seoul National University, Seoul 08826, Korea; Department of Chemistry, Seoul National University, Seoul 08826, Korea; Plant Genomics and Breeding Institute, Seoul National University, Seoul 08826, Korea

**Keywords:** *Arabidopsis*, Hi-C, fine-scale contact domain, gene domain, gene domain analysis tool

## Abstract

**Background:**

Three-dimensional (3D) chromatin organization is emerging as a key factor in gene regulation in eukaryotes. Recent studies using high-resolution Hi-C analysis have explored fine-scale local chromatin contact domains in plants, as exemplified by the basic contact domains established at accessible gene border regions in *Arabidopsis* (*Arabidopsis thaliana*). However, we lack effective tools to identify these contact domains and examine their structural dynamics.

**Results:**

We developed the Hi-C-based 3D Gene Domain analysis Tool (Hi-GDT) to identify fine-scale local chromatin contact domains in plants, with a particular focus on gene borders. Hi-GDT successfully identifies local contact domains, including single-gene and multigene domains, with high reproducibility. Hi-GDT can also be used to discover local contact domains that are differentially organized in association with differences in gene expression between tissue types, genotypes, or in response to environmental stimuli.

**Conclusions:**

Hi-GDT is a valuable tool for identifying genes regulated by dynamic 3D conformational changes, expanding our understanding of the structural and functional relevance of local 3D chromatin organization in plants. Hi-GDT is publicly available at https://github.com/CDL-HongwooLee/Hi-GDT.

**Key Points:**

- We developed the Hi-C-based 3D Gene Domain analysis Tool (Hi-GDT) to identify fine-scale local chromatin contact domains.
- Hi-GDT successfully identifies local contact domains, including single-gene and multigene domains, with high reproducibility.
- Hi-GDT also identifies 3D contact domains that are dynamically organized based on their transcriptional states.

## Introduction

Three-dimensional (3D) chromatin organization is important for the proper structural arrangement of the genome and for controlling gene transcription [1–7]. Eukaryotic genomes are organized into structural units known as topologically associating domains (TADs), which exhibit strong self-interaction and thus appear as triangular shapes in 3D chromatin contact maps [8–10]. TADs can be classified into 2 types depending on the presence or absence of strong contacts at anchor sites (shown as corner dots in 3D Hi-C chromatin contact maps) [11–15]. The 3D contact domains with strong contacts at anchor sites (called loop domains) are established by cohesin and chromatin insulator proteins, including CCCTC-binding factor (CTCF), through a loop-extrusion mechanism [16–18]. By contrast, 3D contact domains without strong contacts at anchor sites (called compartment domains) form independently of CTCF and cohesin and are instead associated with local A/B compartments and epigenetic states [11–13, 15, 19].

The CTCF core architectural protein is not conserved across various plant species [20]. Consistent with this finding, most plant 3D chromatin domains lack strong interactions at anchor sites. Instead, their formation is likely to be dependent on epigenetic states, as observed in tomato (*Solanum lycopersicum*), rice (*Oryza sativa*), sorghum (*Sorghum bicolor*), and foxtail millet (*Setaria italica*) [21–24]. Plants with large genomes, such as maize (*Zea mays*), cotton (*Gossypium hirsutum*), pepper, and wheat (*Triticum aestivum*), also frequently contain 3D chromatin domains demarcated by gene-to-gene loops, which are enriched with active chromatin marks [25–29], suggesting that epigenetic states are critical for organizing the 3D conformation of chromatin in plants. Although the molecular mechanisms of 3D chromatin domain formation vary, the sizes of such domains in these plant species are comparable to those of TADs in mammals. Therefore, conventional TAD analysis tools have been reasonably applied to identify and analyze large 3D chromatin domains in plants.

The compartment domains in plant species with small genomes have recently been explored. Using ultra-deep Hi-C sequencing and advanced Hi-C-based techniques, fine-scale local chromatin domains have been identified in the *Arabidopsis* (*Arabidopsis thaliana*) genome, which are intimately associated with epigenetic states [30–33]. Notably, representative local chromatin domains (called gene domains) are organized at gene border regions with high chromatin accessibility in *Arabidopsis* [34]. These contact domains form at the single-gene scale through a process driven by self-interaction between transcriptional start sites (TSSs) and transcriptional end sites (TESs). They also form at chromatin regions spanning a series of multiple genes with high chromatin accessibility at their borders [34]. Although gene domains are conserved across diverse plant species, including tomato, maize and *Marchantia polymorpha* (*Marchantia*), the functional importance of these fine-scale local chromatin domains in plants has received little attention. One study examined the *Arabidopsis* biosynthetic gene clusters of the specialized metabolites thalianol and marneral, finding that each biosynthetic gene cluster is located in a 3D chromatin domain whose structure is likely reorganized in response to internal and/or external cues to ensure the transcriptional coordination of the associated genes [35]. Otherwise, this area of research has been limited, largely due to the lack of proper analysis tools for such contact domains. In particular, most existing contact domain callers were originally designed for analyzing animal TADs. However, given that gene domains are much smaller than conventional TADs or compartment domains and exhibit relatively low contact frequencies between anchor sites, new tools are needed that can analyze the fine-scale chromatin domains with high sensitivity and precision from high-resolution 3D contact maps.

In this study, we describe the Hi-C-based Gene Domain analysis Tool (Hi-GDT), which we designed to identify 3D contact domains with a focus on gene border regions, enabling more stringent fine-scale gene domain analysis. This tool successfully identified local contact domains, including single-gene and multigene domains, across various Hi-C datasets from *Arabidopsis*, tomato, maize and *Marchantia*. Hi-GDT also identified genes with differential 3D contact domains that are dynamically organized based on their transcriptional states in different tissue types, genotypes, and in response to environmental stimuli. Hi-GDT serves as a valuable tool for analyzing fine-scale local chromatin organization in plants.

## Materials and Methods

### Plant materials

*Arabidopsis* (*A. thaliana*) accession Columbia (Col-0) was grown at 23°C under long-day conditions (16 hours light/8 hours dark) using white fluorescent lamps (120 μmol photons m^−2^ s^−1^). For callus induction, the third and fourth leaves from 2-week-old seedlings were excised and incubated on callus-inducing medium (CIM; Murashige and Skoog medium supplemented with 0.5 μg/mL 2,4-D and 0.05 μg/mL kinetin) for 7 days. The callus tissues were selectively harvested for Hi-C analyses.

### RNA sequencing experiments

Leaf explant samples were harvested immediately after excision (0 day), and leaf explant–derived callus samples were harvested at 7 days after incubation on CIM. RNA libraries were constructed with 1 μg total RNA using a TruSeq stranded mRNA library kit (Illumina) as previously described [36].

### Hi-C experiments and processing

Leaf explant–derived callus samples were harvested at 7 days after incubation on CIM. Hi-C data for callus tissue were generated using an Arima-HiC kit (Arima Genomics) according to the manufacturer’s protocol, followed by next-generation sequencing performed by Macrogen. The publicly available Hi-C sequencing files were downloaded from the NCBI Sequence Read Archive (SRA; Supplementary Table S1) [24, 37–42]. Fastq files were extracted from the SRA files and processed using Juicer (v2.0) (RRID:SCR_017226) [43] with the corresponding reference genome (TAIR10 for *Arabidopsis*, M82v1.0 for tomato, B73v5 for maize, and MpTak1v5 for *Marchantia*). Mapped reads were filtered with a mapping quality cutoff value of 30 in *Arabidopsis*, tomato, and *Marchantia*, whereas no mapping quality cutoff was applied in maize. SCALE normalization was applied to the Hi-C contact matrices to correct for sequencing bias. The compressed binary files (.hic) generated by Juicer were used as inputs for Hi-GDT.

### Single-gene domain analysis by Hi-GDT

**Figure 1:**
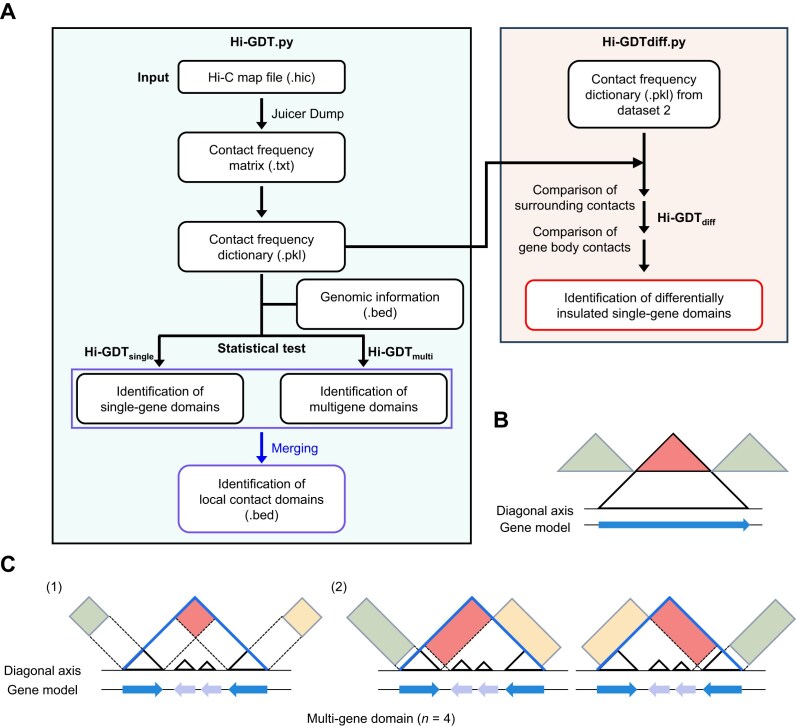
Workflow and strategy of Hi-GDT for identifying local contact domains. (A) The Hi-GDT workflow. Hi-GDT analysis begins with a .hic file generated by Juicer software. Hi-GDT identifies single- and multigene domains (Hi-GDT_single_ and Hi-GDT_multi_, respectively). Hi-GDT also provides an analysis of the structural dynamics of single-gene domains, which are associated with changes in gene expression (Hi-GDT_diff_). (B, C) Schematic diagrams illustrating the target and control regions in a Hi-C contact map, which are defined for gene domain identification by Hi-GDT. The regions used for single-gene domain identification (B) and multigene domain identification (C) are shown. Target regions are shown in red, and control regions are shown in green or yellow. The line at the bottom indicates the diagonal axis in a Hi-C contact map, and the black triangle indicates an individual gene. In (C), target and control regions used to identify quadruple-gene domains are shown as an example. Boundaries of a multigene domain are indicated by blue lines.

Hi-GDT identifies single-gene domains by comparing intragenic contact frequencies with those in designated control regions. Given the relatively high contact frequencies at TSS–TES anchor sites, the intragenic target region near the anchor site is visualized as a triangle in Fig. 1B. The size of this region was defined as half the length of the corresponding gene. The control regions were set to be equidistant with a target region from the diagonal axis of a Hi-C contact map, including intragenic regions at one side of the anchor (Fig. 1B). The distance-normalized intragenic contact frequencies (OE values; observed contact frequency/expected contact frequency) within both intragenic and control regions were collected and tested using the Mann–Whitney *U* test. Genes with significantly higher contact frequencies within intragenic regions compared to the surrounding control regions were defined as single-gene domains. A Benjamini–Hochberg corrected *P* value (*Q* value) cutoff of 0.05 was applied for the identification of single-gene domains from the Hi-C datasets at a 250-bp resolution.

For differential single-gene domain analysis, the OE values within (i) the surrounding 2-kb regions and (ii) the gene body regions of individual genes from 2 different Hi-C datasets were collected. The OE values within the surrounding regions were subjected to a Wilcoxon signed-rank test to identify genes with differential (*Q* < 0.05) surrounding contact frequencies between 2 Hi-C datasets. In addition, a cutoff value of 0.1 for mean fold change of OE values was used to filter the differentially insulated genes that exhibited both increased contact frequencies within gene body regions and decreased contact frequencies within surrounding 2-kb regions. For example, fold change filters of OE values to identify single-gene domains with enhanced insulation under certain treatment condition are applied as follows.


\begin{eqnarray*}
\frac{{\textit{Gene}\ \textit{body}\ O{E}_{\textit{control}}}}{{\textit{Gene}\ \textit{body}\ O{E}_{\textit{treat}}}} < \left( {1 - 0.1} \right),\ \frac{{\textit{Surrounding}\ 2\ kb\ O{E}_{\textit{treat}}}}{{\textit{Surrounding}\ 2\ kb\ O{E}_{\textit{control}}}} < \left( {1 - 0.1} \right)
\end{eqnarray*}

### Multigene domain analysis by Hi-GDT

The identification of multigene domains involved 2 types of comparisons between intradomain regions and outside control regions: (i) comparisons of gene-to-gene contact frequencies at the domain boundaries with those in outside control regions and (ii) comparisons of intradomain contact frequencies at each domain boundary with those in respective control regions (Fig. 1C; Supplementary Fig. S1). The control and target regions were set to be equidistant from the diagonal axis of a Hi-C contact map. Mann–Whitney *U* tests were employed to compare OE values between pairs of target and control regions. A multigene domain was defined as a cluster of adjacent genes that exhibited significantly higher OE values within target regions compared to all their respective control regions.

To analyze multigene domain dynamics, multigene domains were identified at a 500-bp resolution. Multigene domains were identified based on *Q* value cutoffs of 0.05. Dual- and triple-gene domains identified in each specific tissue were compared to analyze the structural dynamics of multigene domains. Genes forming dual-gene domains with a TSS at one boundary, without forming single-gene domains, were used to analyze changes in gene expression.

### Identification of 3D contact domains using existing domain callers

Domain calling with Arrowhead [43], HiCExplorer (RRID:SCR_022111) [44–46], and OnTAD [47] was conducted at a 250-, 500-, and 1,000-bp resolution for benchmarking with Hi-GDT. The parameter “-m 100 -k SCALE” was used for Arrowhead, and the parameter “-penalty 0.1 -minsz 3 -maxsz 200 -hic_norm SCALE” was used for OnTAD. For HiCExplorer, SCALE-normalized .hic files were converted to .h5 format using the hicConvertFormat command, and domain calling was conducted using hicFindTADs with the parameter “–correctForMultipleTesting fdr.”

### Analysis of A and B compartments

To define the local A/B compartment, Pearson’s correlation matrix of the SCALE-normalized Hi-C contact map at a 750-bp resolution was obtained using Juicer Tools [43]. The first eigenvector of the correlation matrix (compartment eigenvector) was calculated using numpy.linalg.eig (RRID:SCR_008633) [48]. For single-gene domain analysis, compartment eigenvector values were measured at the TSS, the center region of the gene body, and the TES. Compartment eigenvector values >0 were assigned to the A compartment, and values <0 were assigned to the B compartment.

### Metaplot analysis

Metaplot analysis for directionality index (DI) score, chromatin accessibility, and compartment eigenvector was performed using deepTools (RRID:SCR_016366) [49]. The DI score with a 5-kb window was calculated at a 250-bp resolution as described previously [8]. The processed BigWig file of Assay for Transposase-Accessible Chromatin (ATAC) sequencing data was downloaded from the Plant Chromatin State Database (PCSD) [50, 51]. The compartment eigenvector at a 750-bp resolution was converted to a BigWig file and used as input for metaplot analysis. For single-gene domain analysis, protein-coding genes without a single-gene domain structure were used for comparison.

### Pile-up analysis

The SCALE-normalized OE values of Hi-C contact matrices at a 250-bp resolution were used for pile-up analysis. The OE values within identified contact domains and their surrounding regions (0.5× domain size) were obtained with a bin size of 10 bp and subsequently resized to an 80 × 80 matrix using bicubic interpolation. For single-gene domain analysis, the images of ($- $)-stranded genes were flipped to synchronize gene orientation. Pile-up analysis was conducted using PileUpDomain.py [52].

### Analysis of chromatin state

Information about the 26 chromatin states was obtained from a previous report [53]. For single-gene domain analysis, chromatin states that overlapped with the TSS, the center of the gene body, and the TES were measured. For multigene domain analysis, chromatin states overlapping with the centers of genes located at multigene domain borders, at internal regions of multigene domains, and at outside regions of multigene domains were investigated. Genes forming single-gene domains were excluded from multigene domain analysis. To investigate the similarity of chromatin states between gene pairs in quadruple-gene domains, both genes in chromatin at the same states (constitutive heterochromatin [H], facultative heterochromatin [F], intergenic [I], and euchromatin [E]) were examined.

### RNA sequencing analysis

The RNA sequencing reads used in this study were downloaded from the SRA database (Supplementary Table S1) [21, 24, 38, 42, 54]. The reads were mapped to the corresponding reference genome using STAR (v2.7.10a) (RRID:SCR_004463) [55] with the parameter “–peOverlapNbasesMin 12 –peOverlapMMp 0.1 –twopassMode Basic.” Transcript abundance was quantified using RSEM (v1.3.1) (RRID:SCR_000262) [56]. Differentially expressed genes (DEGs) and fold change values were identified by Deseq2 (v1.34.0) (RRID:SCR_015687) [57] with a threshold of log_2_(fold change) >1 and *Q* value <0.05. Log_2_(transcripts per million [TPM] + 1) values were used to quantify gene expression levels.

### Calculation of Tau score

Tau score, a measure of tissue-specific gene expression, was calculated as previously described [58]. The expression levels of genes from various *Arabidopsis* tissues were downloaded from a previous report [59]. The corrected reads per kilobase of transcript per million mapped reads (cRPKM) values were used to calculate the Tau score.

### Gene Ontology analysis

Gene Ontology (GO) analysis was performed using AmiGO2 powered by PANTHER (RRID:SCR_004869) [60–62]. The top 10 or 20 significantly enriched GO terms with *P* < 0.05 and number of gene hits >1 were visualized.

### Colinear block analysis

Colinear blocks were identified using a pipeline that utilizes MCScanX (RRID:SCR_022067) [63]. Briefly, interspecies BLASTP analysis (RRID:SCR_001010) [64] was conducted between the *Arabidopsis* and tomato genomes, as well as between the *Arabidopsis* and maize genomes. A pairwise BLASTP was conducted by switching the reference genome, and the best 5 hits with an E-value cutoff of 1 × 10^−10^ were kept for the detection of colinear blocks. Colinear blocks between 2 different species were defined using MCScanX [65] with the default parameters. Among the genes within the colinear blocks in the tomato and maize genomes, those homologous to *Arabidopsis* single-gene domains and adjacent gene pairs homologous to *Arabidopsis* dual-gene domains were collected and visualized.

### Data visualization

All heatmaps and 1-dimensional plots were generated with matplotlib (RRID:SCR_008624) [66] and seaborn (RRID:SCR_018132) [67]. Hi-C maps were visualized using JuiceBox (RRID:SCR_021172) [43].

## Results

### Development of Hi-GDT to analyze local contact domain structures

The discovery of gene domains in plants motivated us to develop Hi-GDT, a specialized tool for identifying fine-scale gene domain structures [34]. Since gene domains are prevalent in *Arabidopsis*, which have been analyzed with various high-resolution Hi-C datasets, we employed high-resolution *Arabidopsis* Hi-C data to validate the performance of our tool [38]. We designed a rule-based algorithm considering key features of gene domains, which include self-interaction patterns and strong insulation at gene borders in Hi-C contact maps, to identify such domains (Hi-GDT_single_ for single-gene domains and Hi-GDT_multi_ for multigene domains) (Fig. 1A). For further analysis of the structural dynamics of single-gene domains, we also developed Hi-GDT_diff_ as a means to identify single-gene domains showing the 3D structural changes that are associated with differential gene expression in different tissue types, genotypes, or in response to environmental stimuli.

Given that single-gene domains display a self-interaction pattern with boundaries of TSSs and TESs that have high chromatin accessibility [34], we compared the distance-normalized intragenic contact frequencies (observed contact frequency/expected contact frequency, or OE) within a genic region defined by a TSS and TES with those of control regions outside genic regions to identify single-gene domains (Fig. 1B). For this comparison, we extracted OE values from the triangular region of a Hi-C contact map whose size was defined based on gene length (0.5× gene length). The target region (red triangle in Fig. 1B) was defined as the intragenic region near gene borders, whereas the control regions (green triangles) were determined based on 2 criteria: (i) the distance from the diagonal axis of a Hi-C contact map should be equal to that of the target region to minimize distance-dependent bias, and (ii) they should consist of interacting chromatin pairs, with one located outside and the other inside a gene body. We conducted a Mann–Whitney *U* test to compare OE values between target and control regions. Genes with significantly higher OE values in intragenic regions compared to those in both control regions were considered to form single-gene domains (Fig. 1B).

To identify multigene domains, we conducted 2 distinct comparisons of OE values between target (red squares) and control regions (green and yellow squares) that were equidistant from the diagonal axis of a Hi-C contact map (Fig. 1C; Supplementary Fig. S1): (i) OE values of gene-to-gene contact sites located at multigene domain boundaries were compared to those within the external control regions, and (ii) OE values at domain boundaries were compared to those in the 2 external control regions positioned in opposite directions (Fig. 1C; Supplementary Fig. S1). Adjacent gene sets that passed all of these statistical comparisons were annotated as multigene domains. In summary, Hi-GDT identified single-gene and multigene domains based on image analysis of Hi-C contact maps.

### Hi-GDT is suitable for analyzing local contact domains in *Arabidopsis*

**Figure 2:**
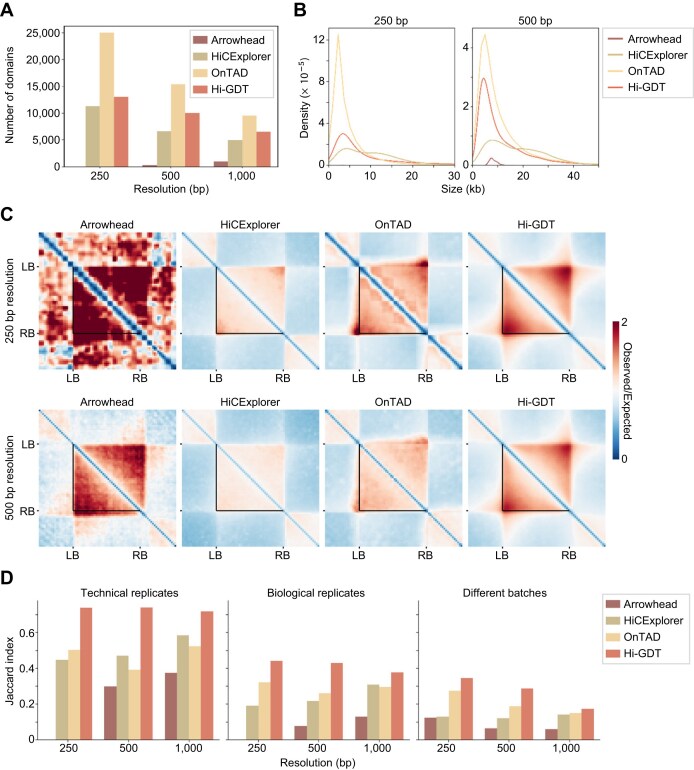
Benchmarking of Hi-GDT against conventional domain callers. (A) Comparison of the number of contact domains identified by Arrowhead, HiCExplorer, OnTAD, and Hi-GDT. Local contact domains were identified at a 250-, 500-, and 1,000-bp resolution. (B) Density plots showing the size distribution of contact domains identified by each domain caller at a 250- and 500-bp resolution. (C) Pile-up images of Hi-C contact matrices for contact domains identified by each domain caller. The images of contact domains identified at a 250-bp (top) and 500-bp (bottom) resolution are shown. Black lines indicate the boundaries of the identified contact domains. (D) Comparison of the Jaccard index values of contact domains identified by each domain caller across different datasets. Domain calling was conducted with 2 technical replicates subsampled (80%) from a merged Hi-C dataset, 2 biological replicates, and 2 distinct datasets from different batches at a 250-, 500-, and 1,000-bp resolution. Jaccard index was calculated as the intersection over the union between 2 sets of identified contact domains.

Since Hi-GDT was designed specifically for gene domain identification, we examined its possible advantages over conventional TAD callers, including Arrowhead [43], HiCExplorer [44–46], and OnTAD [47], in terms of the fine-scale identification of local contact domains in *Arabidopsis*. Hi-GDT identified a substantial number of local contact domains: 13,049 at a 250-bp resolution and 10,089 at a 500-bp resolution. These numbers were higher than the numbers of local contact domains identified by Arrowhead and HiCExplorer (Fig. 2A). The majority of contact domains identified by Hi-GDT were small in terms of size (<10 kb; Fig. 2B; Supplementary Fig. S2A). Notably, conventional domain callers generally predicted local contact domains at gene border regions (Supplementary Fig. S3), in agreement with the local contact domains formed mainly at gene boundaries in *Arabidopsis* [34]. We conducted pile-up analysis of the local contact domains identified by each domain caller to estimate their collective structural features. The contact domains identified by Hi-GDT and Arrowhead showed stronger contact domain structures that were more obvious than those identified by HiCExplorer and OnTAD across all tested resolutions, from 250 to 1,000 bp (Fig. 2C; Supplementary Fig. S2B).

We also assessed the reproducibility of local contact domain identification by each domain caller by comparing the contact domains identified from technical replicates with 80% subsampling of a merged Hi-C dataset, biological replicates, or 2 Hi-C datasets from different batches. The reproducibility of the conventional domain callers for domain identification was low, ranging from 29% to 61% between technical replicates, 4% to 29% between biological replicates, and 1% to 10% between datasets from different batches (Supplementary Fig. S4A), suggesting that conventional domain callers have a limited ability to identify fine-scale local contact domains. In comparison, Hi-GDT showed higher reproducibility (Supplementary Fig. S4A). Since, unlike the other domain callers, Hi-GDT primarily focuses on predefined boundaries (such as TSSs and TESs), we reanalyzed the reproducibility of the conventional domain callers for gene domain identification by allowing the callers to adjust domain boundaries to coincide with gene border regions for a fair comparison. Although this increased the reproducibility of the conventional domain callers to some extent, Hi-GDT still exhibited the highest reproducibility score as well as Jaccard index, which represents the similarity between 2 groups (Fig. 2D; Supplementary Fig. S4B). Taken together, these results indicate that Hi-GDT is highly optimized for fine-scale local contact domain identification in *Arabidopsis* with high reproducibility.

### Validation of single-gene domains identified by Hi-GDT_single_

**Figure 3:**
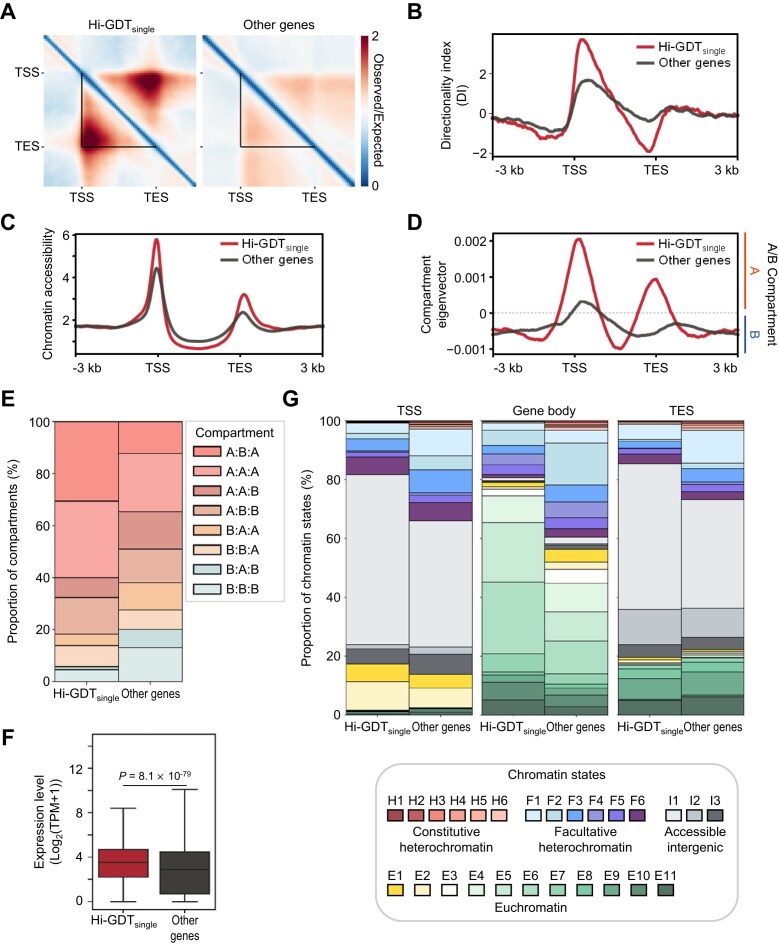
Validation of single-gene domains identified by Hi-GDT_single_. (A) Pile-up images of Hi-C matrices for single-gene domains identified by Hi-GDT_single_. Pile-up images of genes with single-gene domains (Hi-GDT_single_) and without single-gene domains (other genes) at a 250-bp resolution are shown. Black lines indicate gene borders. (B–D) Metagene plots of DI score (B), chromatin accessibility (C), and compartment eigenvector (D) for Hi-GDT_single_-identified (Hi-GDT_single_) or remaining genes (other genes). (E) The proportion of local A/B compartments within genic regions with or without single-gene domains. The local A/B compartments at TSS, gene body, and TES regions were analyzed. (F) Expression levels of genes with or without single-gene domains. Log_2_(transcripts per million [TPM] + 1) values were used to quantify gene expression levels. The *P* value was calculated by a 2-sided Mann–Whitney *U* -test. (G) Distribution of chromatin states at TSS, gene body, and TES regions for genes with or without single-gene domains. Twenty-six chromatin states were used for analysis, including 6 constitutive heterochromatin (H1–H6), 6 facultative heterochromatin (F1–F6), 3 accessible intergenic (I1–I3), and 11 euchromatin (E1–E11) states.

We investigated whether the single-gene domains identified by Hi-GDT_single_ showed key characteristics of previously reported single-gene domains [34]. Pile-up analysis and metagene analysis of DI revealed that the Hi-GDT_single_-identified single-gene domains at a 250-bp resolution were insulated at the TSS and TES regions (Fig. 3A, B). Furthermore, the border regions of these Hi-GDT_single_-identified gene domains had highly accessible chromatin conformations (Fig. 3C), as observed in previously reported single-gene domains [34]. We then analyzed the compartment eigenvector at a high resolution (750 bp) to explore the distribution of local A/B compartments in these single-gene domains. The compartment eigenvector in Hi-GDT_single_-identified gene domains showed positive values (A compartments) at gene domain borders but negative values (B compartments) inside gene domains (Fig. 3D). Consistent with the local compartment distribution of single-gene domains in *Arabidopsis* [34], the Hi-GDT_single_-identified gene domains predominantly had an A-B-A compartment pattern: A compartments at TSS and TES regions and B compartments at gene bodies (Fig. 3D, E). This observation is also in line with the finding that active TSSs are primarily found within local A compartments in both plants and animals [34, 68].

We further explored the association of Hi-GDT_single_-identified gene domains with transcriptional states. In general, genes with local contact domain structures had higher expression levels than control genes without local contact domains, implying that transcriptionally active genes are likely to form single-gene domains (Fig. 3F). We also analyzed the chromatin states of Hi-GDT_single_-identified gene domains based on the 26 previously reported chromatin states, including 6 constitutive heterochromatin states (H1–H6), 6 facultative heterochromatin states (F1–F6), 3 intergenic states with the highest chromatin accessibility (I1–I3), and 11 euchromatin states (E1–E11) [53]. Genes with local contact domain structures were frequently located in euchromatin, whereas genes without these structures were more frequently located in facultative heterochromatin (Fig. 3G), indicating that Hi-GDT_single_ successfully identifies gene domains in transcriptionally active states.

### Validation of multigene domains identified by Hi-GDT_multi_

**Figure 4:**
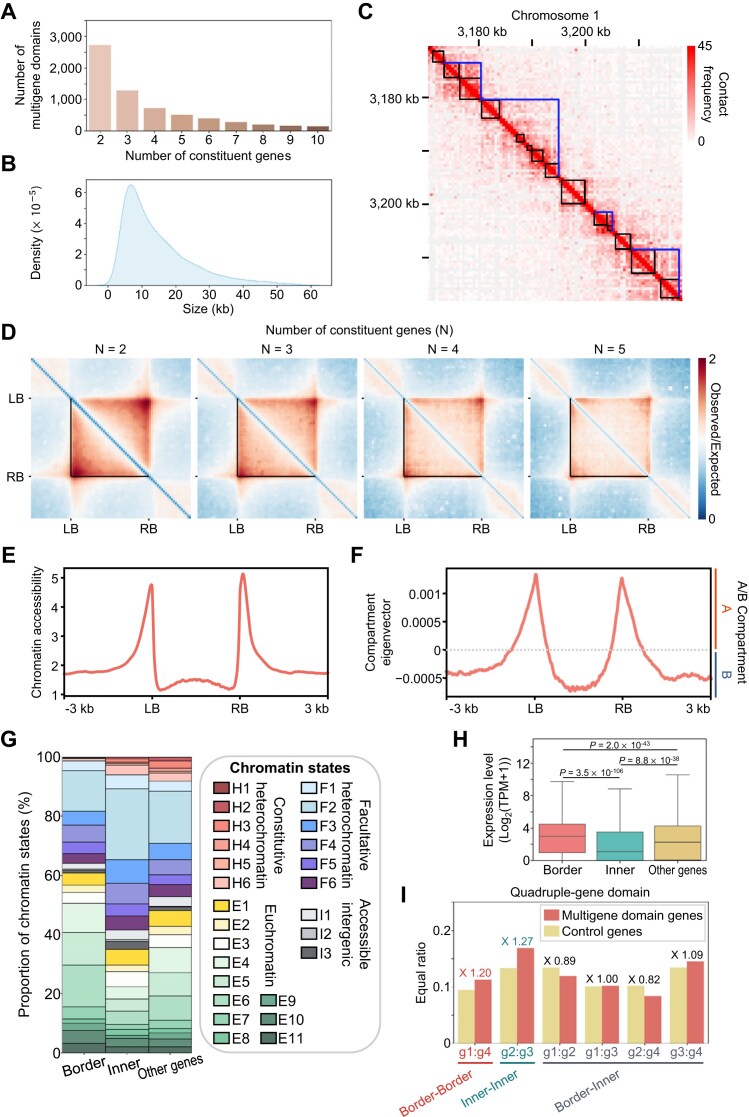
Validation of multigene domains identified by Hi-GDT_multi_. (A) The number of multigene domains identified by Hi-GDT_multi_ based on their number of constituent genes. (B) The size distribution of identified multigene domains. (C) An example region in a Hi-C contact map showing multigene domains identified by Hi-GDT_multi_. The blue lines indicate the boundaries of multigene domains, and black boxes indicate gene borders. (D) Pile-up images of Hi-C matrices for identified multigene domains based on the number of constituent genes. Black lines indicate the boundaries of identified multigene domains. (E, F) Metaplots of chromatin accessibility (E) and compartment eigenvector (F) for the identified multigene domains. (G) The distribution of chromatin states of genes located at boundaries of multigene domains (border), internal regions of multigene domains (inner), and outside of multigene domains (other genes). Twenty-six chromatin states were used for analysis, including 6 constitutive heterochromatin (H1–H6), 6 facultative heterochromatin (F1–F6), 3 accessible intergenic (I1–I3), and 11 euchromatin (E1–E11) states. (H) Expression levels of genes located at boundaries of multigene domains, internal regions of multigene domains, and outside of multigene domains. Log_2_(transcripts per million [TPM] + 1) values were used to quantify gene expression levels. *P* -values were calculated by a Kruskal–Wallis with Dunn’s post hoc test. In (G) and (H), genes forming single-gene domains were excluded. (I) The proportion of gene pairs with similar chromatin states in quadruple-gene domains. Four nearby genes lacking quadruple-gene domains were used for the random control (control genes). Gene 1 (g1) to gene 4 (g4) indicate the sequential order of genes from the 5′ to 3′ direction within a quadruple-gene domain. The ratio between gene pairs from quadruple-gene domains and control nearby genes is indicated above the graph.

In addition to identifying single-gene domains, Hi-GDT also enabled us to identify multigene domains by recognizing their insulating gene borders (Fig. 1C). Using Hi-GDT_multi_, we identified 6,420 multigene domains at a 500-bp resolution, with a median size of 11,550 bp comprising an average of 3.66 genes in *Arabidopsis* (Fig. 4A, B). Numerous multigene domains with distinct domain boundaries were observed in Hi-C contact maps (Fig. 4C; Supplementary Fig. S5A). Pile-up images of Hi-GDT_multi_-identified contact domains also revealed strong domain boundaries, regardless of the number of constituent genes (Fig. 4D). Genes located at the borders of the multigene domain were predominantly oriented in a convergent direction, leading to highly accessible TSS–TSS interactions (Supplementary Fig. S5B–D), which is consistent with previous findings [34].

Similar to single-gene domains, the borders of Hi-GDT_multi_-identified multigene domains exhibited high chromatin accessibility (Fig. 4E) and were usually located in the local A compartment, leading to strong A-B-A compartmentalization (Fig. 4F). Consistent with this finding, genes located at the borders of multigene domains resided in euchromatin regions with high transcriptional activity, whereas genes inside multigene domains were present in facultative heterochromatin, which usually has low transcriptional activity (Fig. 4G, H; Supplementary Fig. S6). Furthermore, pairs of genes located at the contact domain borders were coordinated to have active chromatin states in common, whereas series of internal genes within a multigene domain had similar transcriptionally repressive heterochromatic states (Fig. 4I). These results suggest that Hi-GDT can reliably identify multigene domains in *Arabidopsis*.

### Identification of differentially insulated single-gene domains associated with transcriptional activity depending on tissue type

Taking advantage of Hi-GDT_single_, we analyzed independent Hi-C datasets and compared them to examine the potential dynamics of single-gene domain structures. Our goal was to determine whether Hi-GDT_single_ is reliable across various resolutions and when using different Hi-C datasets. Despite the variation in the number of identified contact domains depending on the resolution (Supplementary Fig. S7A), we obtained relatively consistent results at all resolutions examined: Hi-GDT_single_-identified single-gene domains were characterized by strong anchors at the TSS–TES regions and transcriptionally active states (Fig. 3A, F; Supplementary Fig. S7B, C). We also confirmed the reliability of Hi-GDT_single_ for processing independent *Arabidopsis* Hi-C datasets obtained from different tissues or environmental conditions (Supplementary Fig. S7D).

**Figure 5:**
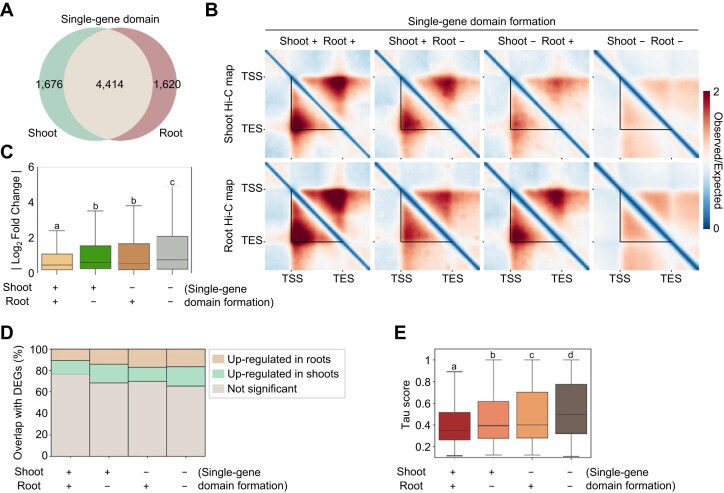
Identification of single-gene domains in shoot and root tissues. (A) Venn diagram illustrating the number of Hi-GDT_single_-identified single-gene domains from shoot and root tissues. (B) Pile-up images of Hi-C matrices for single-gene domains identified from shoot and root tissues based on their tissue specificity. Images of shoot (top) and root (bottom) Hi-C contact maps are shown. Black lines indicate the boundaries of the identified single-gene domains; +, the presence of a single-gene domain; −, the absence of a single-gene domain. (C, D) Changes in gene expression levels for single-gene domains grouped by their tissue specificity. Absolute value log_2_(fold change) of gene expression levels (C) and the proportions of overlap with DEGs between shoot and root tissues (D) in each group are shown. (E) Tau scores of single-gene domains grouped by their tissue specificity. In (C) and (E), different letters indicate statistically significant differences determined by a Kruskal–Wallis with Dunn’s post hoc test (*P* < 0.05).

Given that TADs are closely linked with cellular identity in animals [69–73], we reasoned that local contact domains might differ in *Arabidopsis* depending on tissue identity. We employed Hi-C and RNA sequencing datasets obtained independently from shoot and root tissues and extracted “tissue-specific single-gene domains” [38, 39, 54]. While most single-gene domains were common between shoot and root tissues, ∼25% of single-gene domains from each tissue were defined as tissue-specific single-gene domains (Fig. 5A). Pile-up analysis of these gene domains revealed stronger contact strengths in their respective tissues (Fig. 5B).

Genes with single-gene domains in both tissues exhibited constitutive expression patterns, with little differential expression (Fig. 5C, D) as well as relatively low Tau scores, a measure of tissue-specific expression (Fig. 5E), suggesting that constitutively organized single-gene domains are associated with stable gene expression. Unexpectedly, however, despite the association of single-gene domains with transcriptional activity, there was little correlation between tissue-specific gene domain formation and tissue-specific gene expression (Fig. 5D; Supplementary Fig. S8). Given that gene domain formation is associated with transcriptional activity, our results imply that the Hi-GDT analysis for 2 independent, different Hi-C datasets should be more optimized.

**Figure 6:**
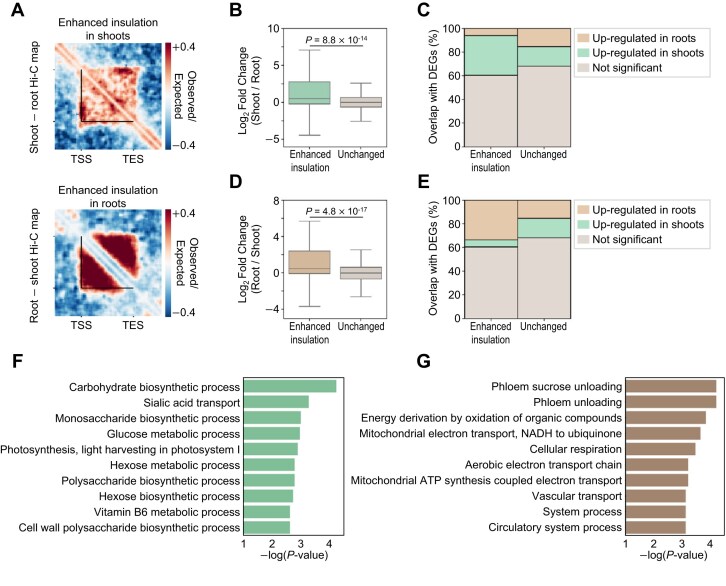
Identification of shoot- and root-specific active single-gene domains. (A) Pile-up images showing differences between shoot and root Hi-C matrices for differentially insulated single-gene domains identified by Hi-GDT_diff_. The differences in pile-up images of single-gene domains with enhanced insulation strength in shoots (upper) and roots (lower) are shown. Black lines indicate the boundaries of the identified single-gene domains. (B, C) Changes in gene expression levels for single-gene domains exhibiting enhanced insulation strength in shoots. Log_2_(fold change) of expression levels (B) and the proportions of overlap with DEGs between shoot and root tissues (C) in each group are shown. (D, E) Changes in gene expression levels for single-gene domains exhibiting enhanced insulation strength in roots. Log_2_(fold change) of expression levels (D) and the proportions of overlap with DEGs (E) in each group are shown. In (B) and (D), *P* values were calculated by 2-sided Mann–Whitney *U* -tests. (F, G) Top 10 significantly enriched GO terms of single-gene domains exhibiting enhanced insulation strength in shoots (F) and roots (G). GO terms with *P* < 0.05 and number of genes in each category >1 are shown.

To better define the linkage of single-gene domain structural dynamics with gene expression changes, we put our focus on the gene domain structure changes of DEGs between shoot and root tissues and found that differences in gene expression correlated positively with intragenic contact frequency changes but also negatively with contact frequency changes within surrounding chromatin regions outside gene bodies, consistent with previous findings (Supplementary Fig. S9) [12, 33, 34]. We therefore developed Hi-GDT_diff_ to compare the gene domain insulation strength, which is represented by chromatin contact frequencies within intragenic regions over those within the 2-kb surrounding regions of individual genes, across different Hi-C datasets. Hi-GDT_diff_ identified differentially insulated single-gene domains in one tissue compared to the other tissue (Fig. 6A; Supplementary Table S2). As expected, single-gene domains with enhanced insulation strength, which are marked by increased intragenic contact frequencies and decreased surrounding contact frequencies, exhibited higher transcription activity in the corresponding tissue (Fig. 6B–E). To further validate our findings, we performed GO analysis on differentially insulated single-gene domains between shoot and root tissues. Shoot-specific GO terms, including “Carbohydrate biosynthetic process” and “Photosynthesis, light harvesting in photosystem I,” were significantly enriched for single-gene domains having enhanced insulation strength in shoots (Fig. 6F; Supplementary Table S3), whereas GO terms related to transport systems, including “Phloem unloading” and “Vascular transport,” were enriched for those with enhanced insulation strength in roots (Fig. 6G; Supplementary Table S3). These results support the relevance of our approach for searching for differentially insulated single-gene domains that are associated with changes in gene expression.

We further applied our tool to other Hi-C datasets generated using *Arabidopsis* shoot tissues exposed to different environmental conditions or using different genotypes. Hi-GDT_diff_ identified only a small number of single-gene domains (*n* = 11) with differential insulation strength between mock and heat shock conditions, even with a weaker *Q* value cutoff (*Q* < 0.1), leading to mild changes in gene expression (Supplementary Fig. S10A, B; Supplementary Table S2) (see Discussion). In contrast, when we applied Hi-GDT_diff_ to different genotypes, it identified a substantial number of differentially insulated single-gene domains between wild type (Col-0) and *bmi1a bmi1b bmi1c* (*bmi1abc*), which also exhibited differential gene expression, supporting the reliability and broad applicability of our tool (Supplementary Fig. S10C–F; Supplementary Table S2). Overall, these results suggest that Hi-GDT_diff_ can be used to compare single-gene domain structures across different, independent Hi-C datasets and extract differentially insulated single-gene domains associated with changes in transcriptional activity. They also suggest that gene domain dynamics are more closely associated with tissue identity than with plant responses to environmental stimuli.

### Single-gene domains are dynamically regulated during cellular reprogramming in *Arabidopsis*

**Figure 7:**
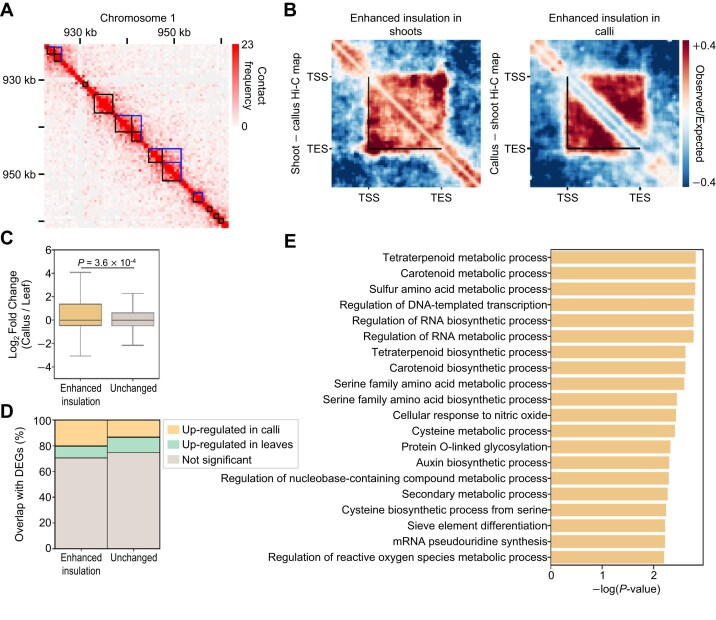
Identification of callus-specific active single-gene domains. (A) Hi-C contact map of callus tissue with local contact domains identified by Hi-GDT. Black squares indicate gene borders, and blue lines indicate the boundaries of the identified contact domains. (B) Pile-up images showing differences between shoot and callus Hi-C matrices for differentially insulated single-gene domains identified by Hi-GDT_diff_. The differences in pile-up images of single-gene domains with enhanced insulation strength in shoot (left) and callus tissues (right) are shown. Black lines indicate the boundaries of the identified single-gene domains. (C, D) Changes in gene expression levels for single-gene domains exhibiting enhanced insulation strength in calli. Log_2_(fold change) of expression levels (C) and the proportions of overlap with DEGs between leaf explant and callus tissues (D) in each group are shown. In (C), the *P* value was calculated by a 2-sided Mann–Whitney *U* test. (E) Top 20 significantly enriched GO terms of single-gene domains exhibiting enhanced insulation strength in calli. GO terms with *P* < 0.05 and number of genes in each category >1 are shown.

Considering the close association between local contact domains and tissue identity, we hypothesized that gene domains are dynamically regulated during the cellular reprogramming process, similar to TADs in animal systems [69, 71, 74–76]. To test this hypothesis, we generated high-resolution Hi-C data with 4 biological replicates from *Arabidopsis* leaf explant–derived calli, which undergo genome-wide reprogramming of cellular identity (Supplementary Table S4). Based on the fact that the 4 replicates of callus Hi-C data showed a high Spearman correlation coefficient with each other, we merged them into single-callus Hi-C data that exhibited a distance-decay model similar to that of shoot and root Hi-C data (Supplementary Fig. S11A, B). We also performed callus RNA sequencing in parallel and confirmed that the data were also highly correlated each other (Supplementary Fig. S11C). The high-resolution Hi-C contact maps of callus tissue showed numerous local contact domains, most with boundaries near gene borders (Fig. 7A), as observed in other *Arabidopsis* tissues (Fig. 4C), confirming that gene domains are prevalent 3D chromatin conformation units in *Arabidopsis*.

We then extracted genes showing dynamic changes in contact domain structures between shoots and calli. Although global Hi-C contact maps of calli showed a high degree of similarity to those of shoots, as evidenced by a Spearman correlation coefficient of 0.88 at a 25-kb resolution, we identified 961 differentially insulated single-gene domains, including 697 genes with enhanced insulation in calli (Fig. 7B; Supplementary Table S2), which were expressed at higher levels in calli compared to leaf explants (Fig. 7C, D). Moreover, GO terms related to callus development, such as “auxin biosynthetic process,” and “regulation of cell growth,” were significantly enriched for these single-gene domains (Fig. 7E; Supplementary Table S3). These results indicate that gene domain structures are tightly reorganized during the reprogramming of tissue identity, which may play a key role in this process.

### Multigene domains are dynamically regulated by tissue type

**Figure 8:**
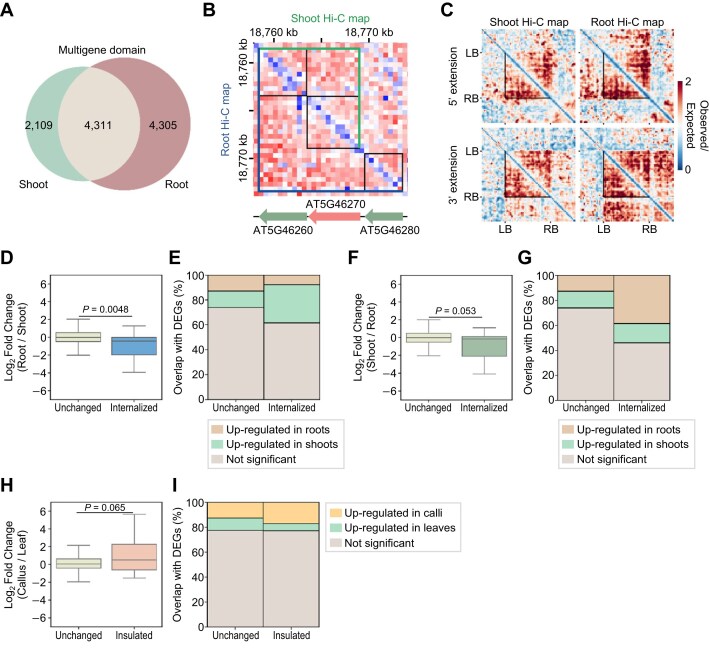
Identification of dynamically regulated multigene domains in different tissue types. (A) Venn diagram illustrating the number of multigene domains identified from shoot and root Hi-C datasets at a 500-bp resolution. (B) An example region showing the transition from a dual-gene domain to a triple-gene domain depending on tissue type. Upper-right triangles represent Hi-C maps in shoots, while lower-left triangles represent Hi-C maps in roots. Black boxes indicate the gene borders, and green and navy blue lines indicate the boundaries of identified multigene domains in shoot and root tissues, respectively. (C) Pile-up images of Hi-C matrices for differential multigene domains between shoot and root tissues. Dual-gene domains specifically identified in shoots and expanded to triple-gene domains in roots were collected. Pile-up images of dual-gene domains expanded to the 5′ direction (top) and the 3′ direction (bottom) in the formation of triple-gene domains are shown. Black lines indicate the boundaries of dual-gene domains. (D, E) Changes in gene expression levels for domain-internalized genes in roots (internalized). Log_2_(fold change) of expression levels (D) and the proportions of overlap with DEGs between shoot and root tissues (E) for domain-internalized genes from dual-gene domains compared to the control genes located in dual-gene domains without structural changes in both tissues (unchanged) are shown. (F, G) Changes in gene expression levels for domain-internalized genes in shoots (internalized). Log_2_(fold change) of expression levels (F) and the proportions of overlap with DEGs between shoot and root tissues (G) for domain-internalized genes from dual-gene domains compared to the control genes located in dual-gene domains without structural changes in both tissues (unchanged) are shown. (H, I) Changes in gene expression levels for domain-insulated genes in callus tissue, which are located within triple-gene domains in shoots but insulated at a border of a dual-gene domain in callus (insulated). Log_2_(fold change) of expression levels (H) and the proportions of overlap with DEGs between leaf explant and callus tissues (I) for domain-insulated genes from triple-gene domains compared to the control genes located in dual-gene domains without structural changes in both tissues (unchanged) are shown. In (D), (F), and (H), *P* values were calculated by 2-sided Mann–Whitney *U* -tests. In (B) to (I), genes that form dual-gene domains with their TSSs at the domain border were included in the analysis.

We then analyzed dynamic changes in multigene domain structures in different tissues. We extracted tissue-specific multigene domains consisting of up to 10 genes: 2,109 shoot-specific, 4,305 root-specific, and 4,311 common multigene domains (Fig. 8A). Since diverse types of structural changes can occur in multigene domains, we focused on a specific case: a tissue-specific change in multigene domain structure from a dual-gene domain to a triple-gene domain with one side of the multigene domain border fixed. For instance, the shoot-specific dual-gene domain consisting of AT5G46260 and AT5G46270 changed to the triple-gene domains incorporating their 3′-downstream gene AT5G46280 in roots (Fig. 8B). Pile-up images demonstrated the expansion of multigene domain structures and tissue-dependent structural dynamics (Fig. 8C; Supplementary Fig. S12A).

This approach allowed us to identify genes with multigene domain dynamics in association with gene expression levels by simply comparing the Hi-GDT_multi_ results from different Hi-C datasets. Genes located at the borders of multigene domains are transcriptionally active, whereas genes within multigene domains are usually silenced (Fig. 4G, H) [34]. Thus, structural changes from a dual-gene domain to a triple-gene domain may induce changes in the expression of genes comprising the domain: border genes of dual-gene domains that become internalized in triple-gene domains during the structural transition are expected to be silenced. It should also be noted that since a TSS has higher chromatin accessibility than a TES [77, 78], changes in gene expression were more obvious for genes whose TSSs, located at domain borders, were moved to the internal region during structural changes (Supplementary Fig. S13A–C).

Genes of shoot-specific dual-gene domains with their TSSs at domain borders that were internalized in roots (domain-internalized genes in roots) exhibited high expression in shoots but low expression in roots (Fig. 8D, E; Supplementary Table S5). We also identified domain-internalized genes in shoots, meaning those that formed dual-gene domains in roots but were internalized into triple-gene domains in shoots. These genes strongly overlapped with DEGs that were more highly expressed in roots than in shoots (Fig. 8F, G; Supplementary Table S5).

Finally, we identified dynamic multigene domain structures by comparing dual- and triple-gene domains between callus and shoot tissues. In particular, we focused on genes in the middle of triple-gene domains in shoots that moved to the borders of dual-gene domains in calli (domain-insulated genes in calli) (Supplementary Fig. S12B, C). These genes, including *CINNAMOYL COA REDUCTASE* (*CCR2*) and *NITRATE TRANSPORTER* (*NRT1.8*), were indeed transcriptionally activated in callus tissues (Fig. 8H, I; Supplementary Table S5). Overall, our results demonstrate that Hi-GDT is an effective tool for identifying local chromatin structures, including single-gene and multigene domains. Furthermore, this tool can also be used to identify gene domains with dynamic structural changes in association with transcriptional activity.

### Application of Hi-GDT in other plant species

**Figure 9:**
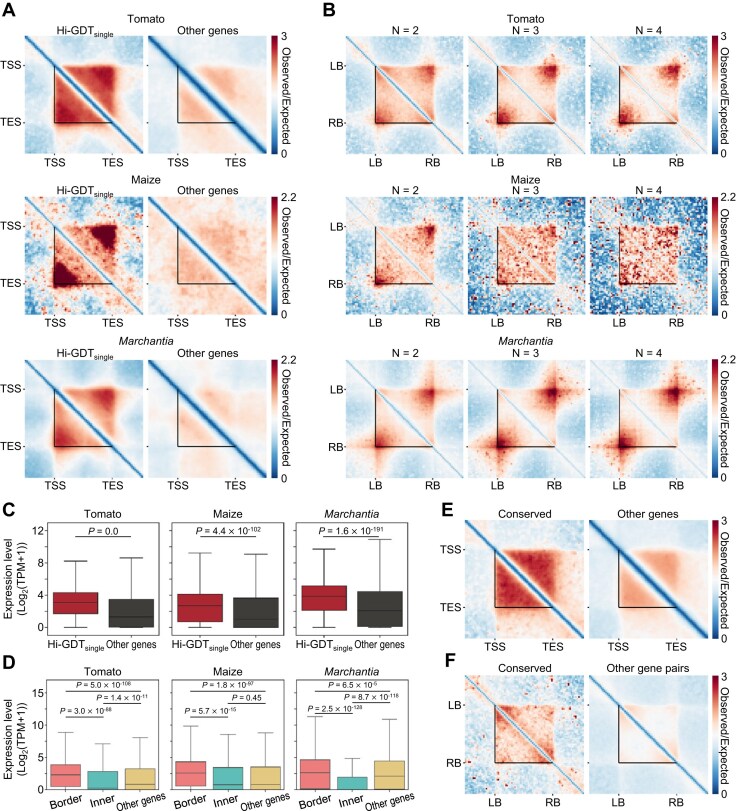
Application of Hi-GDT on other plant species. (A) Pile-up images of Hi-C matrices for single-gene domains identified by Hi-GDT_single_ in tomato, maize, and *Marchantia*. Pile-up images of genes with single-gene domains (Hi-GDT_single_) and without single-gene domains (other genes) at a 250-bp resolution are shown. Black lines indicate gene borders in each species. (B) Pile-up images of Hi-C matrices for identified multigene domains in tomato, maize, and *Marchantia* based on the number of constituent genes. Black lines indicate the boundaries of identified multigene domains. (C) Expression levels of genes with or without single-gene domains. *P* values were calculated by 2-sided Mann–Whitney *U* tests. (D) Expression levels of genes located at boundaries of multigene domains, internal regions of multigene domains, and outside of multigene domains. *P* values were calculated by Kruskal–Wallis with Dunn’s post hoc tests. Genes forming single-gene domains were excluded from all categories. In (C) and (D), log_2_(transcripts per million [TPM] + 1) values were used to quantify gene expression levels. (E) Pile-up image of Hi-C matrices for tomato genes homologous to *Arabidopsis* single-gene domains. All other genes in the tomato genome were used as control genes. Black lines indicate gene borders. (F) Pile-up image of Hi-C matrices for tomato-adjacent gene pairs homologous to *Arabidopsis* dual-gene domains. All other adjacent gene pairs in the tomato genome were used as control gene pairs. Black lines indicate the boundaries of identified adjacent gene pairs. In (E) and (F), homologous genes or gene pairs within the colinear block of tomato and *Arabidopsis* were used.

Given that gene domains have been identified in other plant species, including tomato, maize and *Marchantia* (34), we examined the applicability of Hi-GDT to various plant species. As expected, both Hi-GDT_single_ and Hi-GDT_multi_ successfully identified single-gene and multigene domains characterized by strong internal contact frequencies (Fig. 9A, B; Supplementary Fig. S14). Furthermore, consistent with the findings in *Arabidopsis*, genes with single-gene domain structures or genes located at the borders of multigene domains were transcriptionally active, whereas genes located in the middle of multigene domains were transcriptionally repressed (Fig. 9C, D). However, given a weak association between changes in insulation strength and gene expression levels in single-gene domains from tomato and *Marchantia*, Hi-GDT_diff_ is most effective in high-depth *Arabidopsis* Hi-C analysis (Supplementary Fig. S15).

Taking advantage of the broad applicability of Hi-GDT, we examined whether the gene domain structure is evolutionarily conserved across plant species. To this end, we identified colinear blocks between *Arabidopsis* and tomato genomes, as well as between *Arabidopsis* and maize genomes. Notably, tomato and maize genes, which are homologous to *Arabidopsis* single-gene and dual-gene domains in colinear blocks, exhibited single- or dual-gene domain structures (Fig. 9E, F; Supplementary Fig. S16). Overall, these results indicate that gene domain structures and their formation mechanisms are widely conserved across various plant species.

## Discussion

Numerous bioinformatics tools, including those using deep learning models, have been developed to identify hierarchical chromatin structures in animal genomes [43–47, 79, 80]. Although some of them have been applied to analyze TAD-like structures and compartment domains in large genome plants [28, 43–46], they usually showed low reliability and reproducibility in identifying fine-scale chromatin contact domains (Supplementary Fig. S4A). This limitation is mainly due to the combined effects of weak interaction strength within contact domains and high noise of the Hi-C matrix at a high resolution. To overcome this limitation, we developed a novel tool designed to analyze fine-scale local chromatin contact domains, with a particular focus on single-gene and multigene domains that are established at gene boundaries (Supplementary Fig. S3) [34]. This semi-targeted approach allows for high sensitivity and reproducibility, outperforming existing tools (Fig. 2; Supplementary Fig. S4B). For example, although OnTAD, which is optimized to identify hierarchical (nested) contact domain structures [47], identified a large number of potential fine-scale contact domains (Fig. 2A, B), its low reproducibility may limit the broad application (Fig. 2; Supplementary Fig. S4B). Indeed, our tool successfully identified local contact domains in various plant species, including *Arabidopsis*, tomato, soybean, rice, maize, *Jatropha curcas*, and *Marchantia*, and found that gene domains are conserved across plant species. Hi-GDT showed higher reproducibility than conventional domain callers, enabling effective analysis at a high resolution. This advantage makes it feasible to analyze 3D gene domain dynamics in different tissues and environmental conditions in association with their transcriptional activity.

Our findings confirm that changes in local chromatin structure, especially gene domains, are associated with transcriptional changes. In general, local contact domains identified by Hi-GDT showed high chromatin accessibility at their boundaries (Figs. 3C and 4E). Moreover, RNA polymerase II (RNA Pol II) is highly enriched at the gene domain boundaries (Supplementary Fig. S17), and the binding of multiple transcription factors is crucial for the formation of local 3D chromatin structure [7, 34, 81]. Hence, we suggest that the concerted action of transcription factors and RNA Pol II at gene borders plays a crucial role in shaping local chromatin structure in *Arabidopsis*. Indeed, transcriptional activity and RNA Pol II binding influence the single-gene domain structures of both animals and plants, as revealed by the finding that inhibiting RNA Pol II activity alters local chromatin structure at individual genes [12, 33].

Despite the strong association between local contact domain formation and transcriptional activity, the biochemical relevance of local contact domain formation to the regulation of 3D-conformation-dependent gene expression remains unclear. Considering the extremely short intergenic sequences in the small genome of *Arabidopsis*, local contact domain formation might be particularly important in that proximal *cis*-regulatory elements near gene borders play an additional role in regulating adjacent genes. Indeed, deletion of the promoter region of a gene affects expression of its adjacent gene connected by a promoter–promoter loop [82]. Alternatively, given that these local contact domains frequently form at gene borders, efficient transcription might be facilitated by the anchoring of border regions, similar to the transcription factory model that has been suggested for plants with large genomes [26, 28]. Integrative analysis of Hi-GDT data with intergenic genomic components, such as super enhancers, could provide a comprehensive understanding of the intricate principles governing the relationship between 3D chromatin structure and gene regulation.

Our understanding of the biological relevance of local contact domains in plants also remains limited. Using Hi-GDT, we determined that stable local contact domain structures are associated with constitutive gene expression. In addition, genes with the structural dynamics of local contact domains are frequently related to tissue identity. Notably, the structural plasticity of gene domains in response to environmental stimuli (such as heat) is relatively low (Supplementary Fig. S10), suggesting that local chromatin structures are more tightly linked with cell or tissue identity compared to environmental conditions. In this context, the low structural dynamics of gene domains in response to environmental stimuli might also be due to the dilution effect of heterogeneous cell types. Hi-GDT should facilitate functional studies of local contact domains in *Arabidopsis* and provide broader insight into the biological impact of local chromatin organization in plants.

We placed a particular emphasis on the relevance of our tool for analyzing dynamic structural changes associated with transcriptional activity at the gene scale, as this issue has not previously been analyzed due to the lack of optimal analytical tools. Although the analysis of local chromatin domains requires high sequencing depth for precise analysis, this limitation can be overcome using micrococcal nuclease–based techniques such as highly sensitive transposase-mediated analysis of chromatin (Hi-TrAC), Micro-C, or chemical-crosslinking assisted proximity capture (CAP-C) [81–84]. Notably, the newer cutting-edge techniques known as CAP-C and Micro-C-XL can be used to generate contact maps at an extremely high resolution of 200 bp to support analysis of fine-scale chromatin structure in plants [33, 82]. Hi-GDT is compatible with high-resolution contact maps, and the combinations of these techniques with Hi-GDT should be a powerful tool for analyzing fine-scale local contact domains.

## Availability of Source Code and Requirements

- Project name: Hi-GDT
- Project homepage: https://github.com/CDL-HongwooLee/Hi-GDT
- Operating system(s): Linux
- Programming language: Python
- Other requirements: Python 3.8, subprocess, numpy, pickle, multiprocessing, PIL, functools, itertools, scipy
- License: MIT license
- RRID:SCR_025798
- Software Heritage PID: swh:1:snp:a4d7864a7adfdf451f1058edde2c0014d7b51fe6

## Supplementary Material

giaf020_Supplemental_File

giaf020_GIGA-D-24-00394_Original_Submission

giaf020_GIGA-D-24-00394_Revision_1

giaf020_Response_to_Reviewer_Comments_Original_Submission

giaf020_Reviewer_1_Report_Original_Submission Hao Wu -- 11/9/2024

giaf020_Reviewer_1_Report_Revision_1 Hao Wu -- 1/12/2025

giaf020_Reviewer_2_Report_Original_Submission Yusen Ye -- 11/20/2024

giaf020_Reviewer_2_Report_Revision_1 Yusen Ye -- 1/20/2025

## Data Availability

All publicly available datasets used in this study are listed in Supplementary Table S1. The source codes and step-by-step instructions of Hi-GDT are available from the GitHub repository [85]. Snapshots of the code and data are available in Software Heritage [86]. Raw data and all implemented codes used in this study have been deposited at Zenodo [52]. Hi-C and RNA-seq data produced in this study were deposited in NCBI under accession number PRJNA1112149.

## Additional Files

**Supplementary Fig. S1**. Strategy of Hi-GDT_multi_ for multigene domain identification. (A, B) Schematic diagrams illustrating the target and control regions in a Hi-C contact map, which were defined for multigene domain identification by Hi-GDT_multi_. The regions compared for multigene domains composed of 2 (A) and 3 (B) adjacent genes are shown. Target regions are shown in red and control regions in green or yellow. Boundaries of a multigene domain are indicated by blue lines. The lines at the bottom indicate the diagonal axis in a Hi-C contact map, and each black triangle indicates an individual gene.

**Supplementary Fig. S2**. Benchmarking of Hi-GDT against conventional domain callers. (A) Density plot illustrating the size distribution of contact domains identified by each domain caller at 1,000 bp resolution. (B) Pile-up images of Hi-C contact matrices for contact domains identified by each domain caller at 1,000 bp resolution. Black lines indicate the boundaries of the identified contact domains.

**Supplementary Fig. S3**. Genomic contexts at boundary regions of local contact domains identified by conventional domain callers. (A , B) Proportions of domain boundaries overlapping with gene border, gene body, and intergenic regions at various resolutions. The overlap between identified contact domains and intra/intergenic regions of annotated protein-coding genes (A) or their 5-kb-shifted control regions (B) was investigated. The size of the domain boundaries was set to match the given resolution, and the size of gene borders was defined as 200 bp from both TSSs or TESs in order to measure the overlap.

**Supplementary Fig. S4**. Comparison of the reproducibility of local contact domains identified by Hi-GDT and conventional domain callers. (A) Reproducibility rates of local contact domain identification by conventional domain callers between technical replicates subsampled (80%) from a merged Hi-C dataset, biological replicates, and datasets from different batches. The identified contact domains, which exactly match their boundaries with another contact domain, were considered to represent a reproducible prediction. (B) Comparison of the reproducibility rates of local contact domain identification, with domain boundaries adjusted to coincide with gene border regions. In (A) and (B), reproducibility rates were calculated as the ratio of the number of overlapping domains to the number of domains in a single dataset.

**Supplementary Fig. S5**. Characteristics of Hi-GDTmulti-identified multigene domains. (A) An example region in a Hi-C OE map annotated with multigene domains identified by Hi-GDTmulti. The blue lines indicate the boundaries of multigene domains, and black boxes indicate gene borders. (B-D) The number of identified multigene domains categorized based on the orientations of their constituent genes. The distribution of gene orientations of constituent genes within dual-gene domains (B), triple-gene domains (C), and quadruple-gene domains (D) are shown. The colors indicate the gene orientations of gene pairs at the boundaries of multigene domains: convergent (pink), parallel (blue), and divergent (brown).

**Supplementary Fig. S6**. Expression levels of constituent genes of Hi-GDTmulti-identified multigene domains. (A-C) Expression levels of constituent genes of multigene domains. The expression levels of dual-gene domains (A), triple-gene domains (B), and quadruple-gene domains (C) identified by Hi-GDTmulti are shown. Gene 1 to Gene 4 indicate the sequential order of genes from the 5’ to 3’ direction within a multigene domain. Genes outside multigene domains were used as a control (other genes). Log2(transcripts per million [TPM] + 1) values were used to quantify gene expression levels. Different letters indicate statistically significant differences determined by a Kruskal–Wallis with Dunn’s post-hoc test (P-value < 0.05).

**Supplementary Fig. S7**. Identification of single-gene domains by Hi-GDTsingle at various resolutions using different datasets. (A-C) Profiles of single-gene domains identified by Hi-GDTsingle at 1-restriction fragment (1 f) and 2 f, 250 bp, and 500 bp resolution. In (A), the number of single-gene domains identified by Hi-GDTsingle at each resolution are shown. Pile-up images (B) and expression levels (C) for Hi-GDTsingle-identified (Hi- GDTsingle) or remaining (other genes) genes at each resolution are shown. In (C), P-values were calculated by two-sided Mann–Whitney U-tests. (D) Pile-up images of Hi-C matrices for genes with or without single-gene domains identified by Hi-GDTsingle from various Hi-C datasets at 250 bp resolution. Hi-C datasets obtained from roots or heat shock-exposed shoots were used for pile-up analysis. In (B) and (D), black lines indicate gene borders.

**Supplementary Fig. S8**. Transcript levels of genes with shoot- and root-specific single-gene domains. (A-C) Transcript levels of genes with single-gene domains grouped by their tissue specificity. Expression levels in shoot and root tissues (A), log2(fold change) of expression levels (B), and the number of single-gene domains overlapping with DEGs between shoot and root tissues (C) in each group are shown. +, the presence of a single-gene domain; −, the absence of a single-gene domain. In (A), log2(transcripts per million [TPM] + 1) values were used to quantify expression levels. In (A) and (B), different letters indicate statistically significant differences determined by a Kruskal-Wallis with Dunn’s post-hoc test (P-value < 0.05).

**Supplementary Fig. S9**. Structural alterations of single-gene domains in association with changes in gene expression. (A and B) Pile-up images showing differences between shoot and root Hi-C matrices for differentially expressed genes. Up-regulated genes (A) and down-regulated genes (B) in roots compared to shoots were included in the pile-up analysis. Genes with no significant changes in expression levels were used as control genes. Differences in pile-up images between up-regulated and control genes (A), and between down-regulated and control genes (B) are shown. Black lines indicate the boundaries of the identified single-gene domains.

**Supplementary Fig. S10**. Identification of differentially-insulated single-gene domains between different Hi-C datasets. (A and B) Changes in gene expression levels for single-gene domains with enhanced insulation after heat shock treatment. Log2(fold change) of expression levels (A) and the proportions of overlap with DEGs between mock and heat conditions (B) in each group are shown. (C and D) Changes in gene expression levels for single-gene domains with enhanced insulation in WT compared to bmi1abc mutant. Log2(fold change) of expression levels (C) and the proportions of overlap with DEGs between WT and bmi1abc mutant (D) in each group are shown. (E and F) Changes in gene expression levels for single-gene domains with enhanced insulation in bmi1abc mutant compared to WT. Log2(fold change) of expression levels (E) and the proportions of overlap with DEGs between WT and bmi1abc mutant (F) in each group are shown. In (A), (C) and (E), P-values were calculated by two-sided Mann–Whitney U-tests.

**Supplementary Fig. S11**. Validation of callus Hi-C and RNA-seq data. (A) Correlation plot for four callus Hi-C replicates. Spearman’s correlation coefficient values between each Hi-C replicate at 25-kb resolution are shown. (B) A distance-decay plot of callus Hi-C data with other public Hi-C data from shoot and root tissues. Four replicates were merged to estimate distance-decay model of callus Hi-C data. SCALE-normalized Hi-C counts at 25-kb resolution were estimated. (C) A correlation plot for RNA-seq data produced from leaf explant and callus tissues. Spearman’s correlation coefficient values between all RNA-seq replicates are shown. Hierarchical clustering was performed based on the Spearman’s correlation coefficient values.

**Supplementary Fig. S12**. Pile-up analysis of multigene domains with dynamic structural changes between different tissue types. (A) Pile-up images of Hi-C matrices for differential multigene domains between shoot and root tissues. Dual-gene domains specifically identified in shoots and expanded to triple-gene domains in roots were collected. Pile-up images of triple-gene domains expanded in the 5’ direction (top) or the 3’ direction (bottom) from dual-gene domains are shown. (B and C) Pile-up images of Hi-C matrices for differential multigene domains between shoot and callus tissues. Triple-gene domains specifically identified in shoots and insulated to dual-gene domains in callus were collected. In (B), pile-up images of dual-gene domains expanded to the 5’ direction (top) or the 3’ direction (bottom) in the formation of triple-gene domains are shown. In (C), pile-up images of triple-gene domains expanded in the 5’ direction (top) or the 3’ direction (bottom) from dual-gene domains are shown. In (A) and (C), black lines indicate the boundaries of triplegene domains. In (B), black lines indicate the boundaries of dual-gene domains.

**Supplementary Fig. S13**. Changes in the expression levels of genes with structural changes in multigene domains. (A and B) Changes in gene expression levels for domain-internalized genes in roots (internalized). Log2(fold change) of expression levels (A) and the proportions of overlap of DEGs between shoot and root tissues (B) for domain-internalized genes from dual-gene domains as compared to the control genes located in dual-gene domains without structural changes in both tissues (unchanged) are shown. In (A), the P-value was calculated by a two-sided Mann–Whitney U-test. (C) Log2(fold change) of gene expression levels between shoot and root tissues based on the orientations of constituent genes within dual-gene domains. Expression changes of dual-gene domains expanded to the 5’ direction (left) or the 3’ direction (right) in the formation of triple-gene domains are shown. Dual-gene domains expanded to triple-gene domains with internalization of TSSs at dual-gene domain borders are shown in orange.

**Supplementary Fig. S14**. Gene domains identified by Hi-GDT in various plant species. (A) Pile-up images of Hi-C matrices for single-gene domains identified by Hi-GDTsingle in rice, soybean, and Jatropha curcas (J. curcas). Pile-up images of genes with single-gene domains (Hi-GDTsingle) and without single-gene domains (other genes) at 250-bp resolution are shown. Black lines indicate gene borders in each species. (B) Pile-up images of Hi-C matrices for identified multigene domains in rice, soybean, and J. curcas based on the number of constituent genes. Black lines indicate the boundaries of identified multigene domains.

**Supplementary Fig. S15**. Identification of differentially-insulated single-gene domains between different Hi-C datasets from tomato and Marchantia. (A and B) Changes in gene expression levels for single-gene domains with reduced insulation after heat shock treatment in tomato. Log2(fold change) of expression levels (A) and the proportions of overlap with DEGs between mock and heat conditions (B) in each group are shown. (C and D) Changes in gene expression levels for single-gene domains with enhanced insulation after heat shock treatment in tomato. Log2(fold change) of expression levels (C) and the proportions of overlap with DEGs between mock and heat conditions (D) in each group are shown. (E and F) Changes in gene expression levels for singlegene domains with enhanced insulation in WT compared to tcp1 mutant in Marchantia. Log2(fold change) of expression levels (E) and the proportions of overlap with DEGs between WT and tcp1 mutant (F) in each group are shown. (G and H) Changes in gene expression levels for single-gene domains with enhanced insulation in tcp1 compared to WT in Marchantia. Log2(fold change) of expression levels (G) and the proportions of overlap with DEGs between WT and tcp1 mutant (H) in each group are shown. In (A), (C), (E) and (G), P-values were calculated by two-sided Mann–Whitney U-tests.

**Supplementary Fig. S16**. Local contact domain structures of maize genes homologous to Arabidopsis single-gene domains and dual-gene domains (A) Pile-up image of Hi-C matrices for maize genes homologous to Arabidopsis single-gene domains. All other genes in maize genome were used as control genes. Black lines indicate gene borders. (B) Pileup image of Hi-C matrices for maize adjacent gene pairs homologous to Arabidopsis dual-gene domains. All other gene pairs in maize genome were used as control gene pairs. Black lines indicate the boundaries of identified adjacent gene pairs. Homologous genes or gene pairs within the colinear block of maize and Arabidopsis were used.

**Supplementary Fig. S17**. Metaplots of RNA polymerase II (RNA Pol II) enrichment at the identified local contact domains. (A, B) RNA Pol II enrichment level at the single-gene domains (A) and multigene domains (B) identified by Hi-GDT. In (A), single-gene domains identified by Hi-GDTsingle and remaining genes (other genes) are shown. (C) RNA Pol II enrichment level at the local contact domains identified by HiCExplorer, OnTAD, or Hi-GDT at 250 bp resolution. The metaplot of those identified by Arrowhead is not displayed due to the low number of identified domains.

**Supplementary Table S1**. NGS datasets used in this study.

**Supplementary Table S2**. Differentially insulated single-gene domains identified by Hi-GDT_diff_.

**Supplementary Table S3**. GO enrichment analysis for differentially insulated single-gene domains.

**Supplementary Table S4**. Reads alignment statistics of callus Hi-C and RNA-seq data.

**Supplementary Table S5**. Domain-internalized and domain-boundary genes of multigene domains in different tissue types.

## Abbreviations

3D: three-dimensional; DEG: differentially expressed gene; DI: directionality index; GO: Gene Ontology; OE: observed contact frequency/expected contact frequency; TAD: topologically associating domain; TES: transcriptional end site; TPM: transcripts per million; TSS: transcriptional start site.
