# Supplementary material for: Hi-GDT: A Hi-C-based 3D gene domain analysis tool for analyzing local chromatin contacts in plants
Source: Gigascience. 2025 Mar 21;14:giaf020. doi: 10.1093/gigascience/giaf020 (PMC11927400; doi:10.1093/gigascience/giaf020)
Supplement: giaf020_Supplemental_File [file giaf020_supplemental_file.zip › Supplementary Figures.pdf]

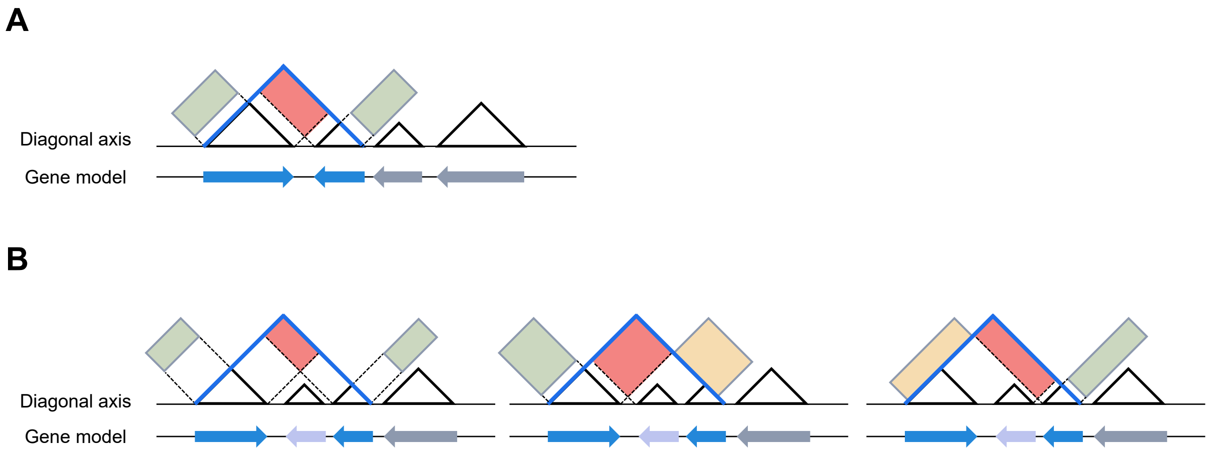

Supplementary Figure S1. Strategy of Hi-GDT$_{multi}$ for multigene domain identification.
(**A** and **B**) Schematic diagrams illustrating the target and control regions in a Hi-C contact map, which were defined for multigene domain identification by Hi-GDT$_{multi}$. The regions compared for multigene domains composed of two (**A**) and three (**B**) adjacent genes are shown. Target regions are shown in red and control regions in green or yellow. Boundaries of a multigene domain are indicated by blue lines. The lines at the bottom indicate the diagonal axis in a Hi-C contact map, and each black triangle indicates an individual gene.

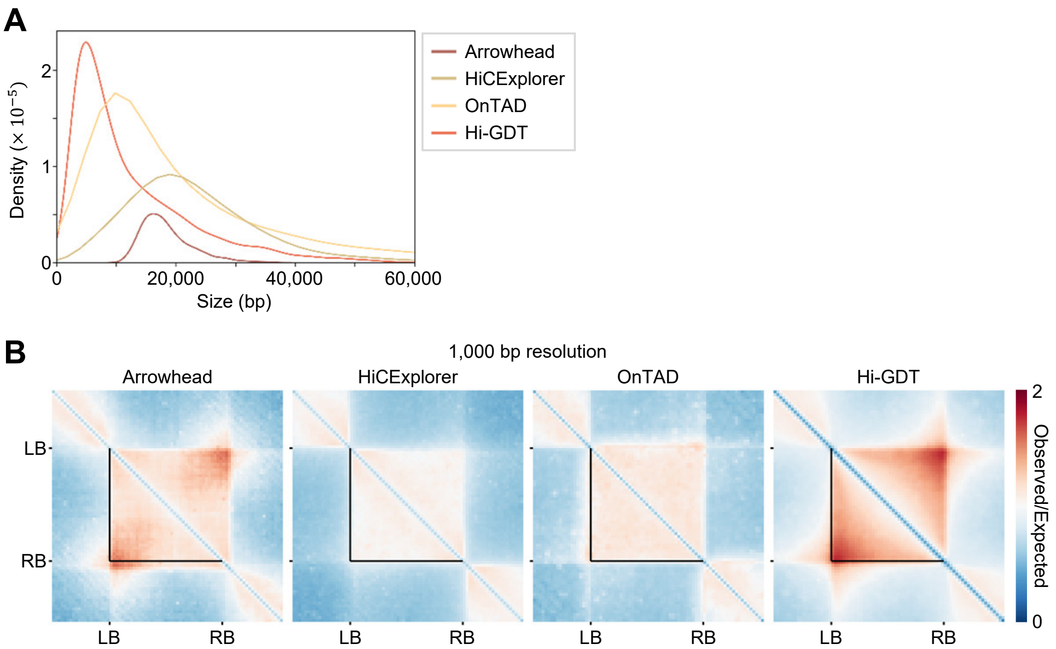

Supplementary Figure S2. Benchmarking of Hi-GDT against conventional domain callers.
(**A**) Density plot illustrating the size distribution of contact domains identified by each domain caller at 1,000 bp resolution. (**B**) Pile-up images of Hi-C contact matrices for contact domains identified by each domain caller at 1,000 bp resolution. Black lines indicate the boundaries of the identified contact domains.

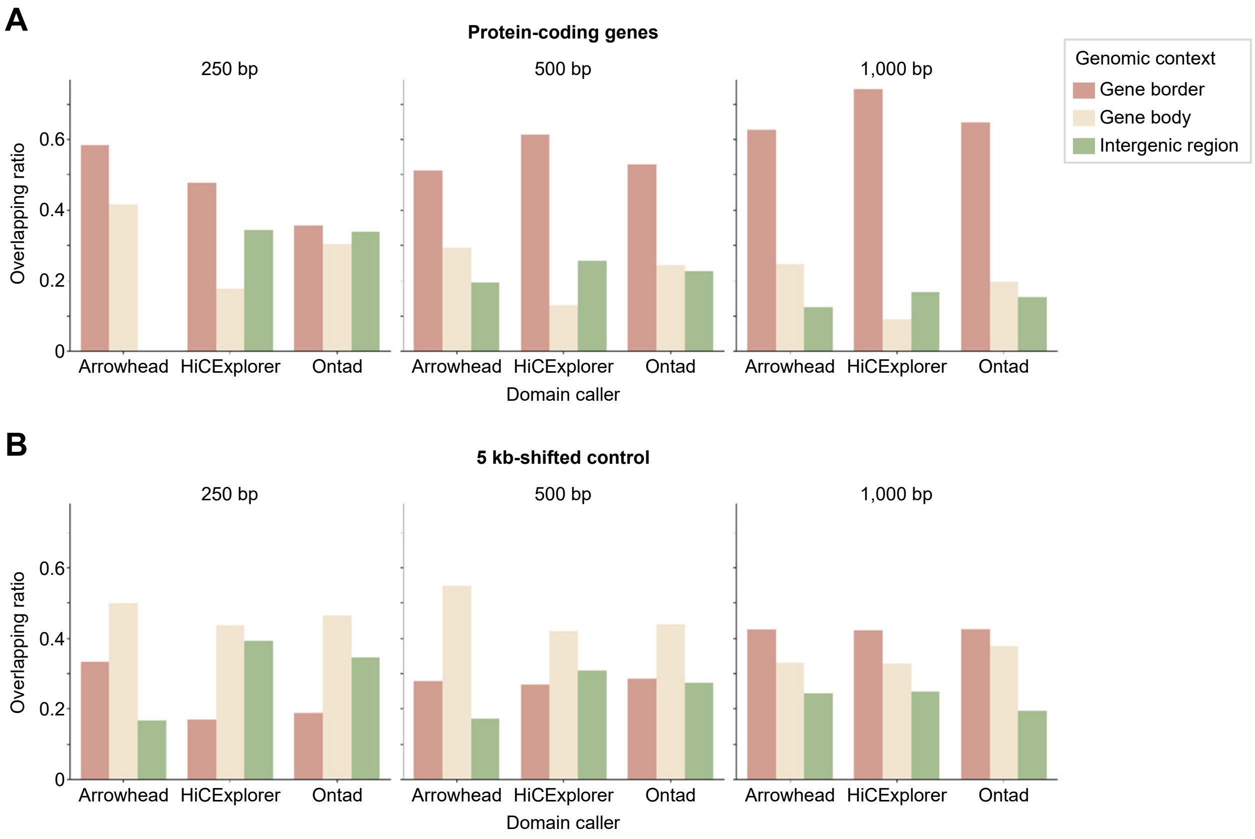

Supplementary Figure S3. Genomic contexts at boundary regions of local contact domains identified by conventional domain callers.

(**A** and **B**) Proportions of domain boundaries overlapping with gene border, gene body, and intergenic regions at various resolutions. The overlap between identified contact domains and intra/intergenic regions of annotated protein-coding genes (**A**) or their 5-kb-shifted control regions (**B**) was investigated. The size of the domain boundaries was set to match the given resolution, and the size of gene borders was defined as 200 bp from both TSSs or TESs in order to measure the overlap.

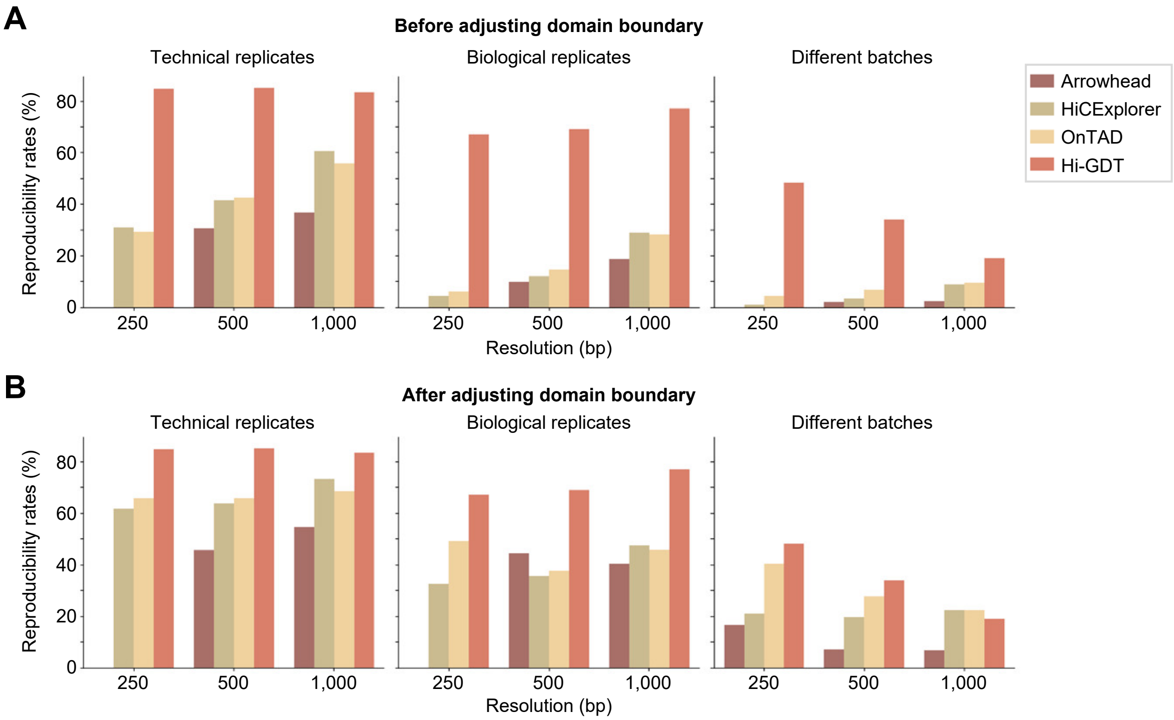

Supplementary Figure S4. Comparison of the reproducibility of local contact domains identified by Hi-GDT and conventional domain callers.

(**A**) Reproducibility rates of local contact domain identification by conventional domain callers between technical replicates subsampled (80%) from a merged Hi-C dataset, biological replicates, and datasets from different batches. The identified contact domains, which exactly match their boundaries with another contact domain, were considered to represent a reproducible prediction. (**B**) Comparison of the reproducibility rates of local contact domain identification, with domain boundaries adjusted to coincide with gene border regions. In (**A**) and (**B**), reproducibility rates were calculated as the ratio of the number of overlapping domains to the number of domains in a single dataset.

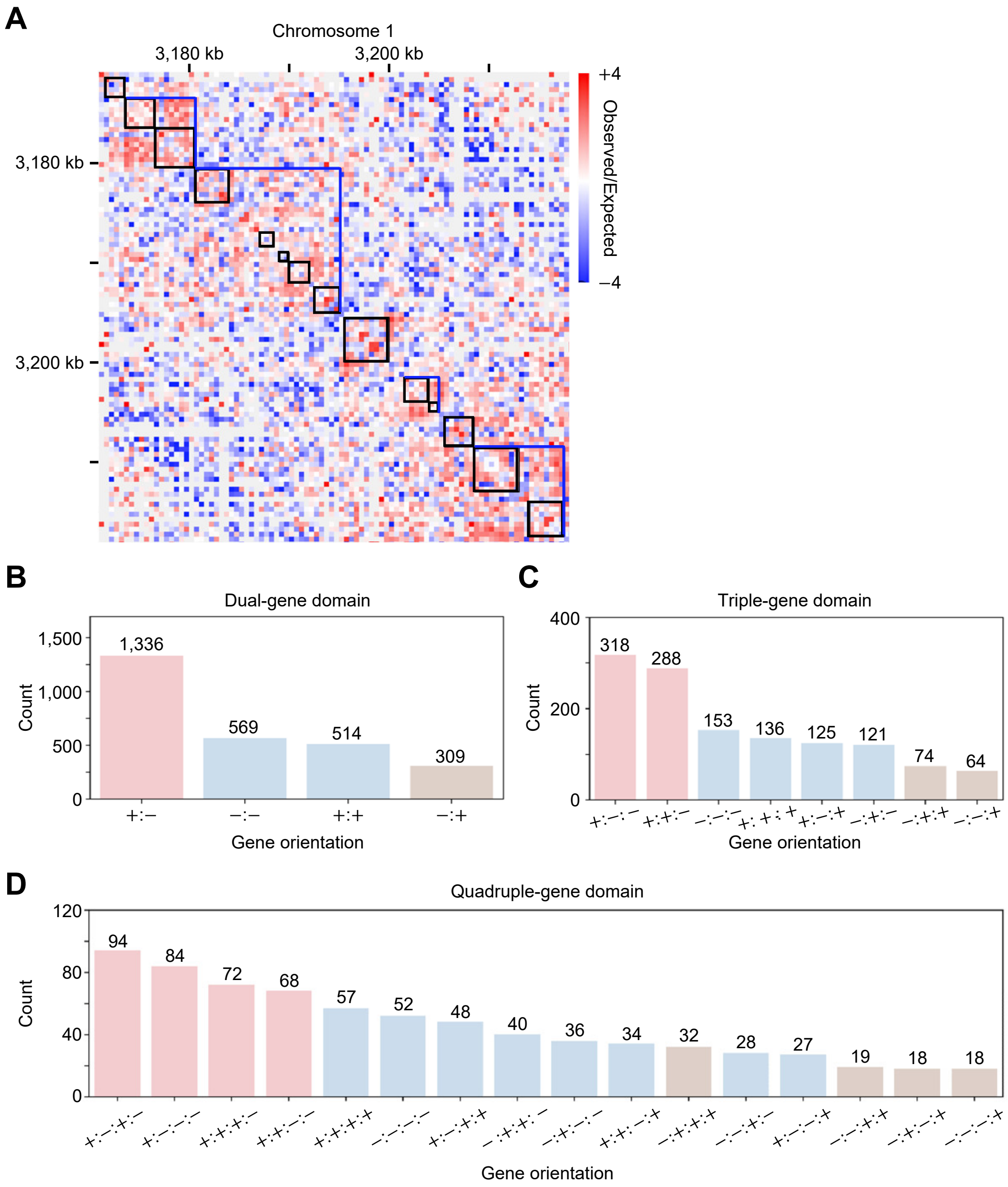

Supplementary Figure S5. Characteristics of Hi-GDT$_{multi}$-identified multigene domains.
(**A**) An example region in a Hi-C OE map annotated with multigene domains identified by Hi-GDT$_{multi}$. The blue lines indicate the boundaries of multigene domains, and black boxes indicate gene borders. (**B**-**D**) The number of identified multigene domains categorized based on the orientations of their constituent genes. The distribution of gene orientations of constituent genes within dual-gene domains (**B**), triple-gene domains (**C**), and quadruple-gene domains (**D**) are shown. The colors indicate the gene orientations of gene pairs at the boundaries of multigene domains: convergent (pink), parallel (blue), and divergent (brown).

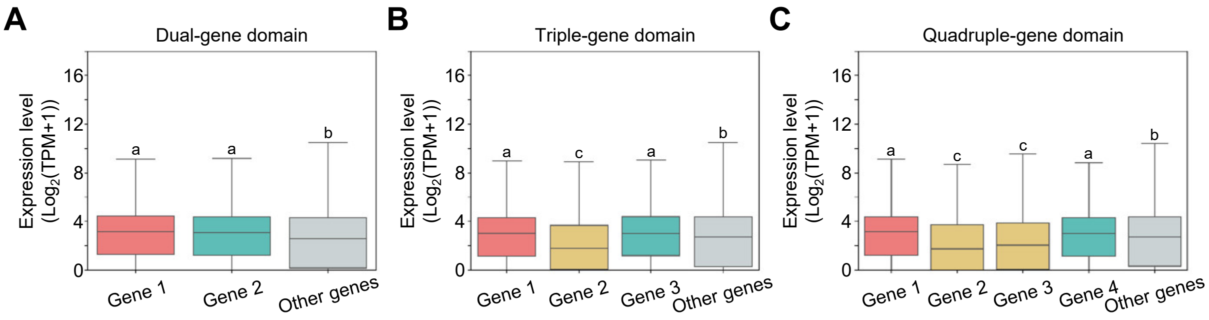

Supplementary Figure S6. Expression levels of constituent genes of Hi-GDT$_{multi}$-identified multigene domains.

(**A-C**) Expression levels of constituent genes of multigene domains. The expression levels of dual-gene domains (**A**), triple-gene domains (**B**), and quadruple-gene domains (**C**) identified by Hi-GDT$_{multi}$ are shown. Gene 1 to Gene 4 indicate the sequential order of genes from the 5' to 3' direction within a multigene domain. Genes outside multigene domains were used as a control (other genes). Log_2(transcripts per million [TPM] + 1) values were used to quantify gene expression levels. Different letters indicate statistically significant differences determined by a Kruskal–Wallis with Dunn's post-hoc test (*P*-value < 0.05).

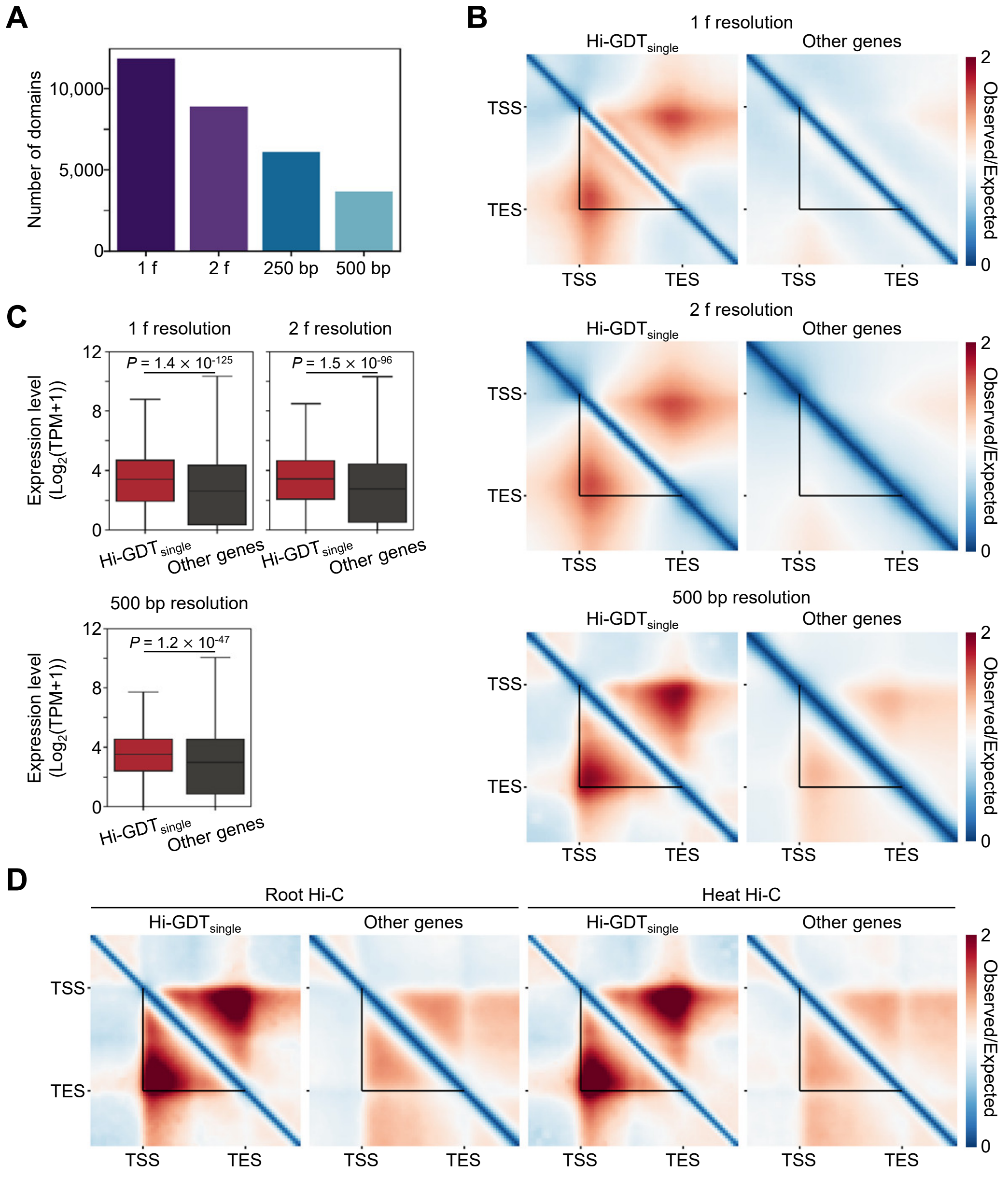

Supplementary Figure S7. Identification of single-gene domains by Hi-GDT$_{\mathbf{single}}$ at various resolutions using different datasets.

(**A**-**C**) Profiles of single-gene domains identified by Hi-GDT$_{single}$ at 1-restriction fragment (1 f) and 2 f, 250 bp, and 500 bp resolution. In (**A**), the number of single-gene domains identified by Hi-GDT$_{single}$ at each resolution are shown. Pile-up images (**B**) and expression levels (**C**) for Hi-GDT$_{single}$-identified (Hi-GDT$_{single}$) or remaining (other genes) genes at each resolution are shown. In (**C**), *P*-values were calculated by two-sided Mann–Whitney U-tests. (**D**) Pile-up images of Hi-C matrices for genes with or without single-gene domains identified by Hi-GDT$_{single}$ from various Hi-C datasets at 250 bp resolution. Hi-C datasets obtained from roots or heat shock-exposed shoots were used for pile-up analysis. In (**B**) and (**D**), black lines indicate gene borders.

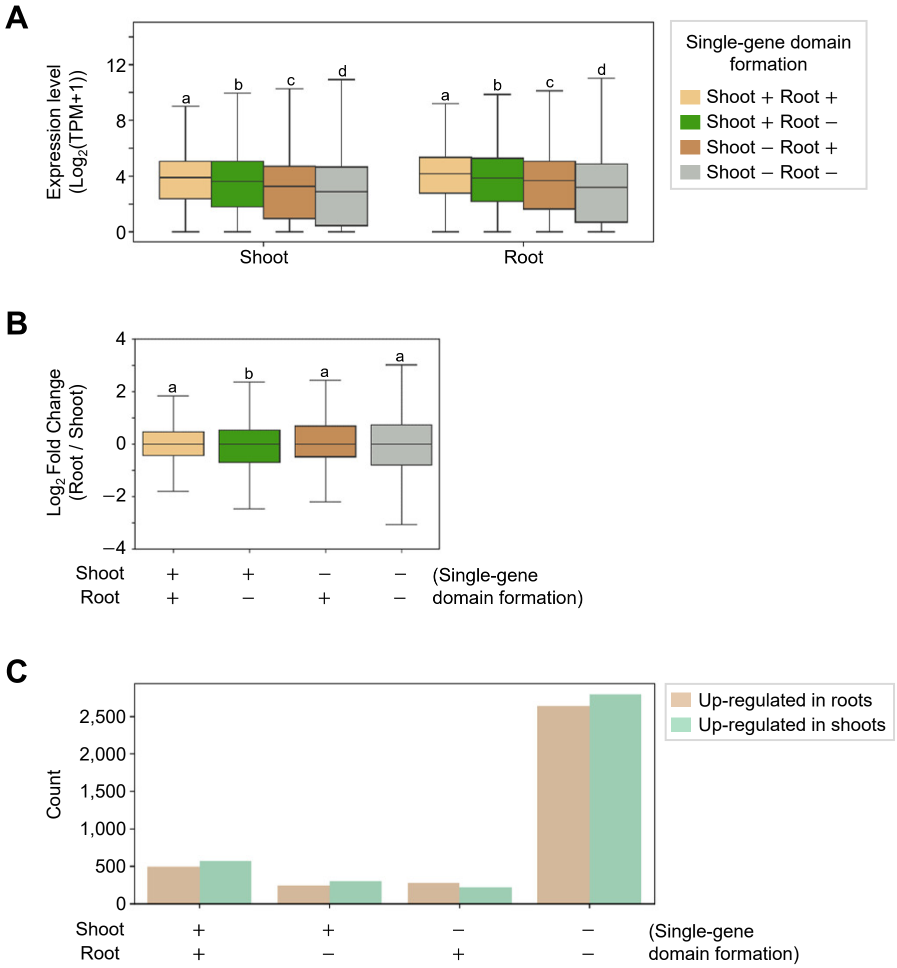

Supplementary Figure S8. Transcript levels of genes with shoot- and root-specific single-gene domains.

(**A**-**C**) Transcript levels of genes with single-gene domains grouped by their tissue specificity. Expression levels in shoot and root tissues (**A**), $\log_2$(fold change) of expression levels (**B**), and the number of single-gene domains overlapping with DEGs between shoot and root tissues (**C**) in each group are shown. +, the presence of a single-gene domain; −, the absence of a single-gene domain. In (**A**), $\log_2$(transcripts per million [TPM] + 1) values were used to quantify expression levels. In (**A**) and (**B**), different letters indicate statistically significant differences determined by a Kruskal-Wallis with Dunn's post-hoc test (*P*-value < 0.05).

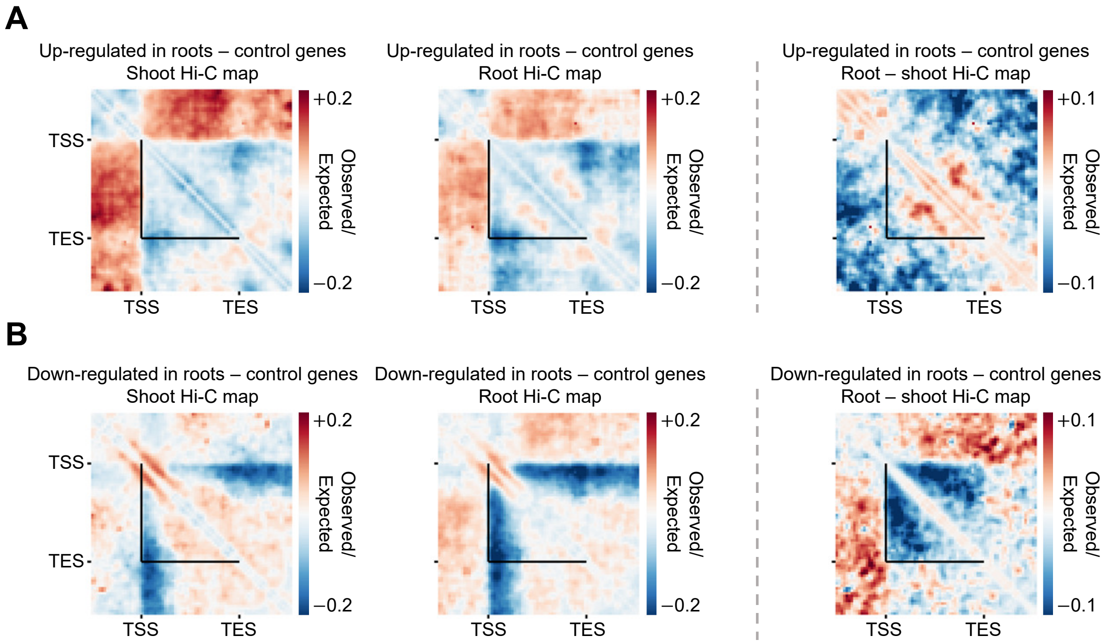

Supplementary Figure S9. Structural alterations of single-gene domains in association with changes in gene expression.

(**A** and **B**) Pile-up images showing differences between shoot and root Hi-C matrices for differentially expressed genes. Up-regulated genes (**A**) and down-regulated genes (**B**) in roots compared to shoots were included in the pile-up analysis. Genes with no significant changes in expression levels were used as control genes. Differences in pile-up images between up-regulated and control genes (**A**), and between down-regulated and control genes (**B**) are shown. Black lines indicate the boundaries of the identified single-gene domains.

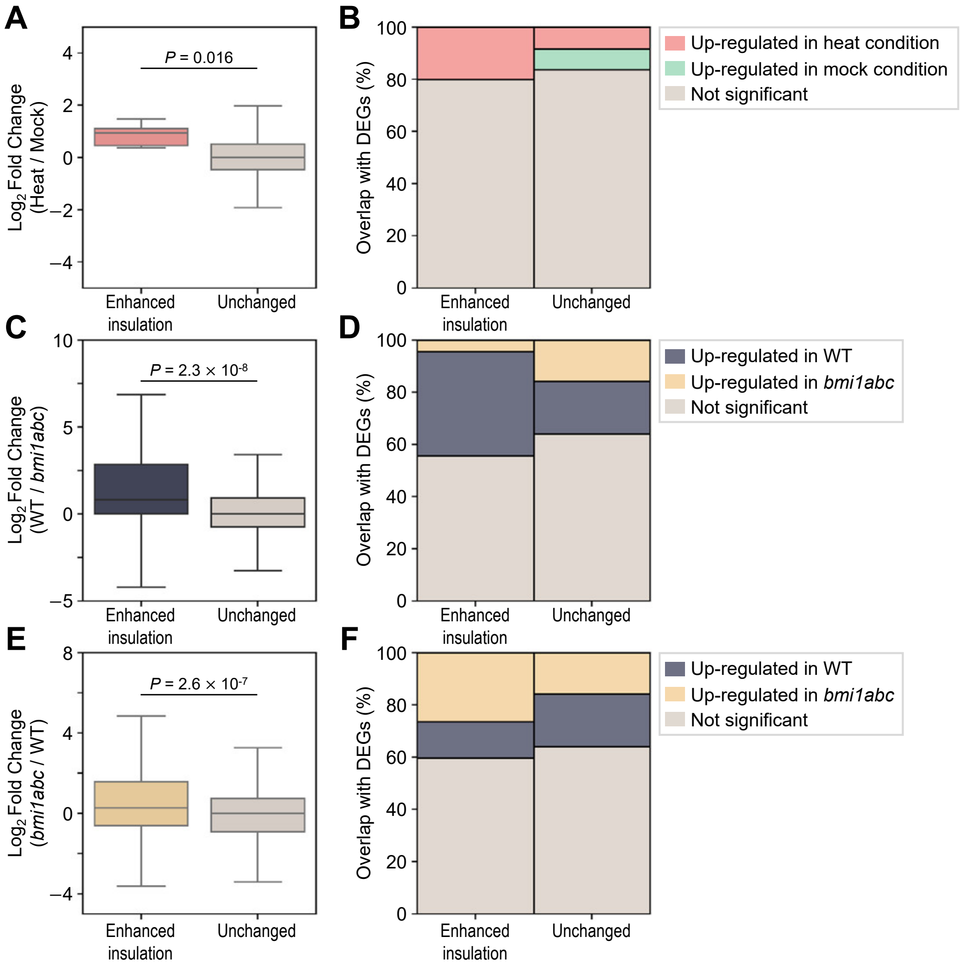

Supplementary Figure S10. Identification of differentially-insulated single-gene domains between different Hi-C datasets.

(**A** and **B**) Changes in gene expression levels for single-gene domains with enhanced insulation after heat shock treatment. Log_2(fold change) of expression levels (**A**) and the proportions of overlap with DEGs between mock and heat conditions (**B**) in each group are shown. (**C** and **D**) Changes in gene expression levels for single-gene domains with enhanced insulation in WT compared to *bmi1abc* mutant. Log_2(fold change) of expression levels (**C**) and the proportions of overlap with DEGs between WT and *bmi1abc* mutant (**D**) in each group are shown. (**E** and **F**) Changes in gene expression levels for single-gene domains with enhanced insulation in *bmi1abc* mutant compared to WT. Log_2(fold change) of expression levels (**E**) and the proportions of overlap with DEGs between WT and *bmi1abc* mutant (**F**) in each group are shown. In (**A**), (**C**) and (**E**), *P*-values were calculated by two-sided Mann–Whitney U-tests.

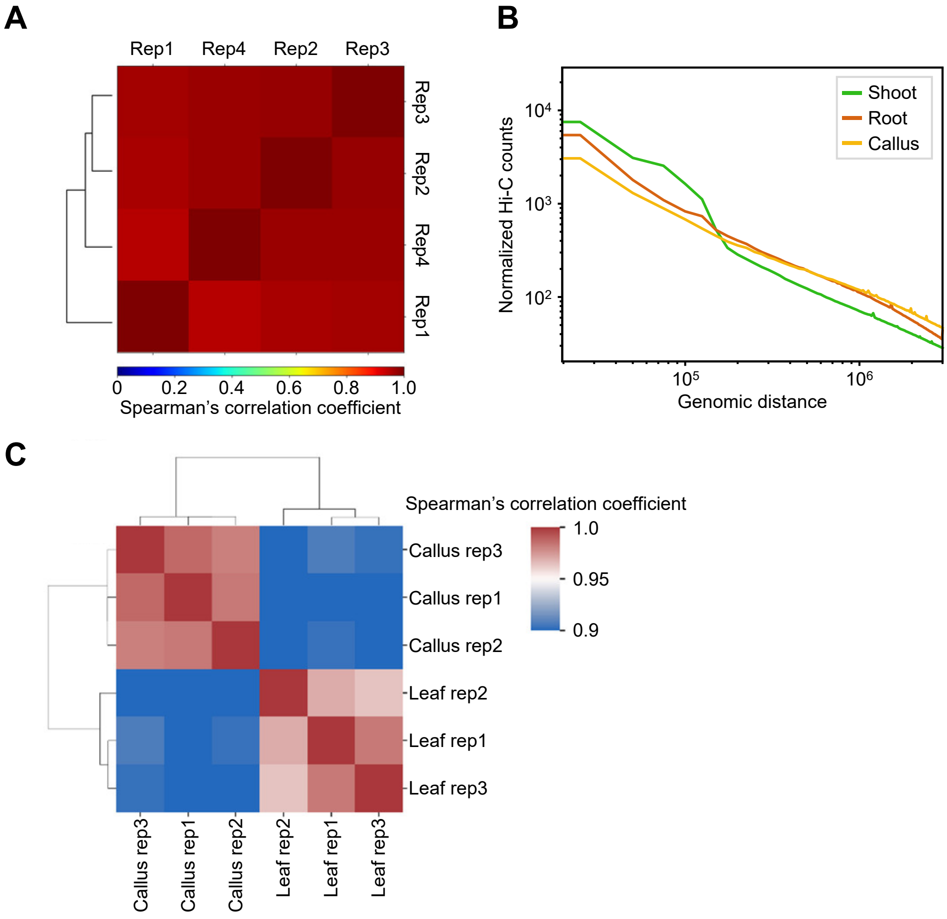

Supplementary Figure S11. Validation of callus Hi-C and RNA-seq data.
(A) Correlation plot for four callus Hi-C replicates. Spearman's correlation coefficient values between each Hi-C replicate at 25-kb resolution are shown. (**B**) A distance-decay plot of callus Hi-C data with other public Hi-C data from shoot and root tissues. Four replicates were merged to estimate distance-decay model of callus Hi-C data. SCALE-normalized Hi-C counts at 25-kb resolution were estimated. (**C**) A correlation plot for RNA-seq data produced from leaf explant and callus tissues. Spearman's correlation coefficient values between all RNA-seq replicates are shown. Hierarchical clustering was performed based on the Spearman's correlation coefficient values.

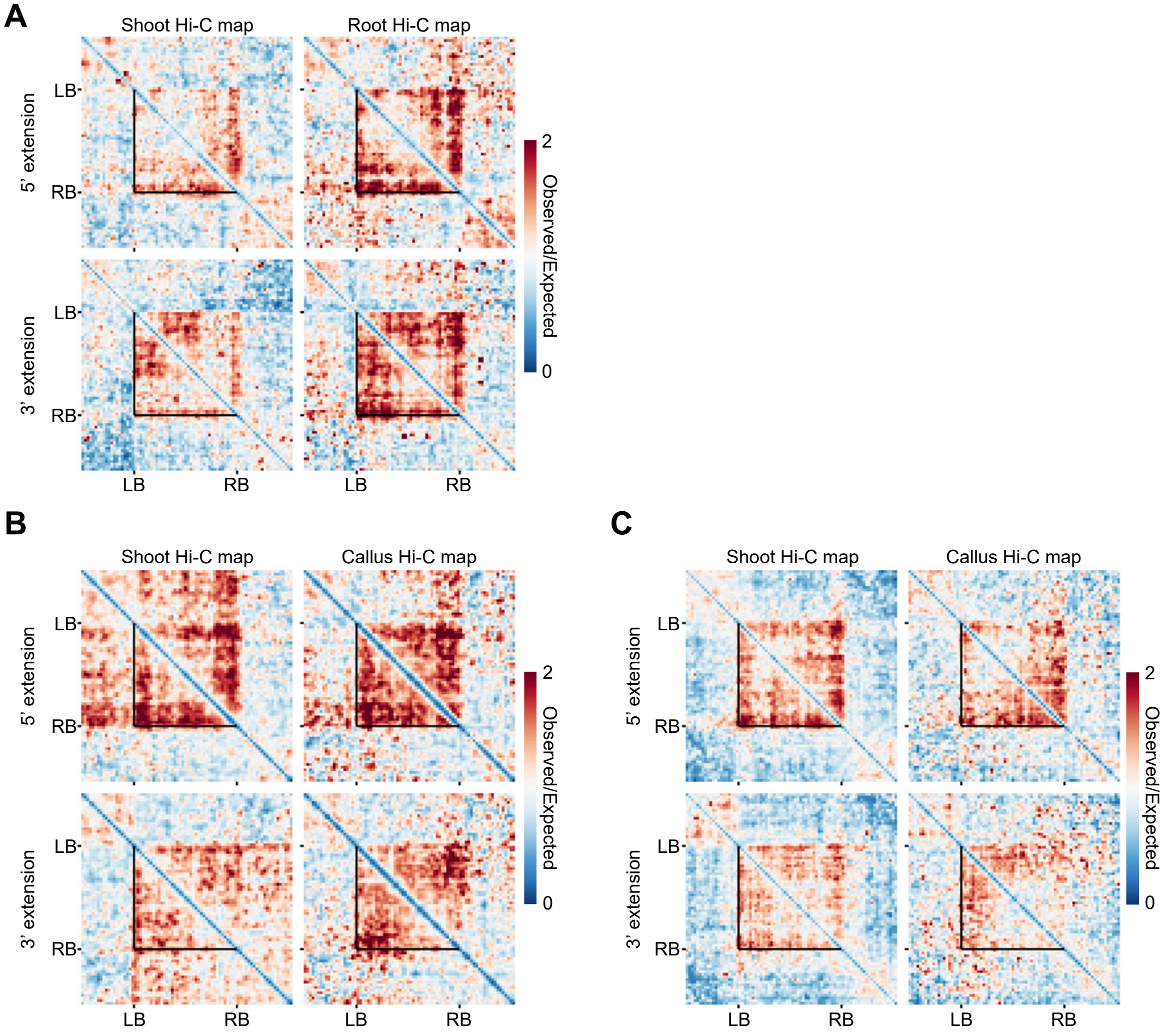

Supplementary Figure S12. Pile-up analysis of multigene domains with dynamic structural changes between different tissue types.

(**A**) Pile-up images of Hi-C matrices for differential multigene domains between shoot and root tissues. Dual-gene domains specifically identified in shoots and expanded to triple-gene domains in roots were collected. Pile-up images of triple-gene domains expanded in the 5' direction (top) or the 3' direction (bottom) from dual-gene domains are shown. (**B** and **C**) Pile-up images of Hi-C matrices for differential multigene domains between shoot and callus tissues. Triple-gene domains specifically identified in shoots and insulated to dual-gene domains in callus were collected. In (**B**), pile-up images of dual-gene domains expanded to the 5' direction (top) or the 3' direction (bottom) in the formation of triple-gene domains are shown. In (**C**), pile-up images of triple-gene domains expanded in the 5' direction (top) or the 3' direction (bottom) from dual-gene domains are shown. In (**A**) and (**C**), black lines indicate the boundaries of triple-gene domains. In (**B**), black lines indicate the boundaries of dual-gene domains.

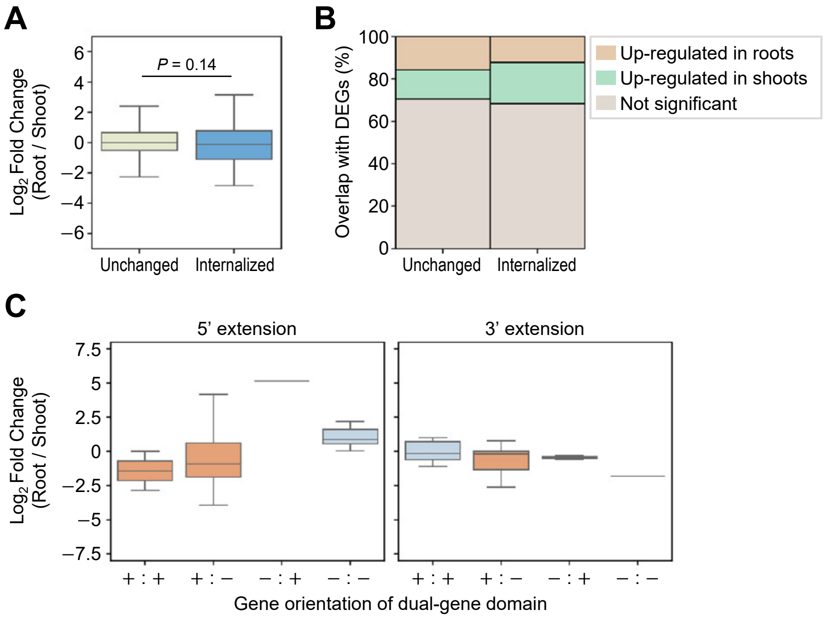

Supplementary Figure S13. Changes in the expression levels of genes with structural changes in multigene domains.

(**A** and **B**) Changes in gene expression levels for domain-internalized genes in roots (internalized). Log_2(fold change) of expression levels (**A**) and the proportions of overlap of DEGs between shoot and root tissues (**B**) for domain-internalized genes from dual-gene domains as compared to the control genes located in dual-gene domains without structural changes in both tissues (unchanged) are shown. In (**A**), the *P*-value was calculated by a two-sided Mann–Whitney U-test. (**C**) Log_2(fold change) of gene expression levels between shoot and root tissues based on the orientations of constituent genes within dual-gene domains. Expression changes of dual-gene domains expanded to the 5' direction (left) or the 3' direction (right) in the formation of triple-gene domains are shown. Dual-gene domains expanded to triple-gene domains with internalization of TSSs at dual-gene domain borders are shown in orange.

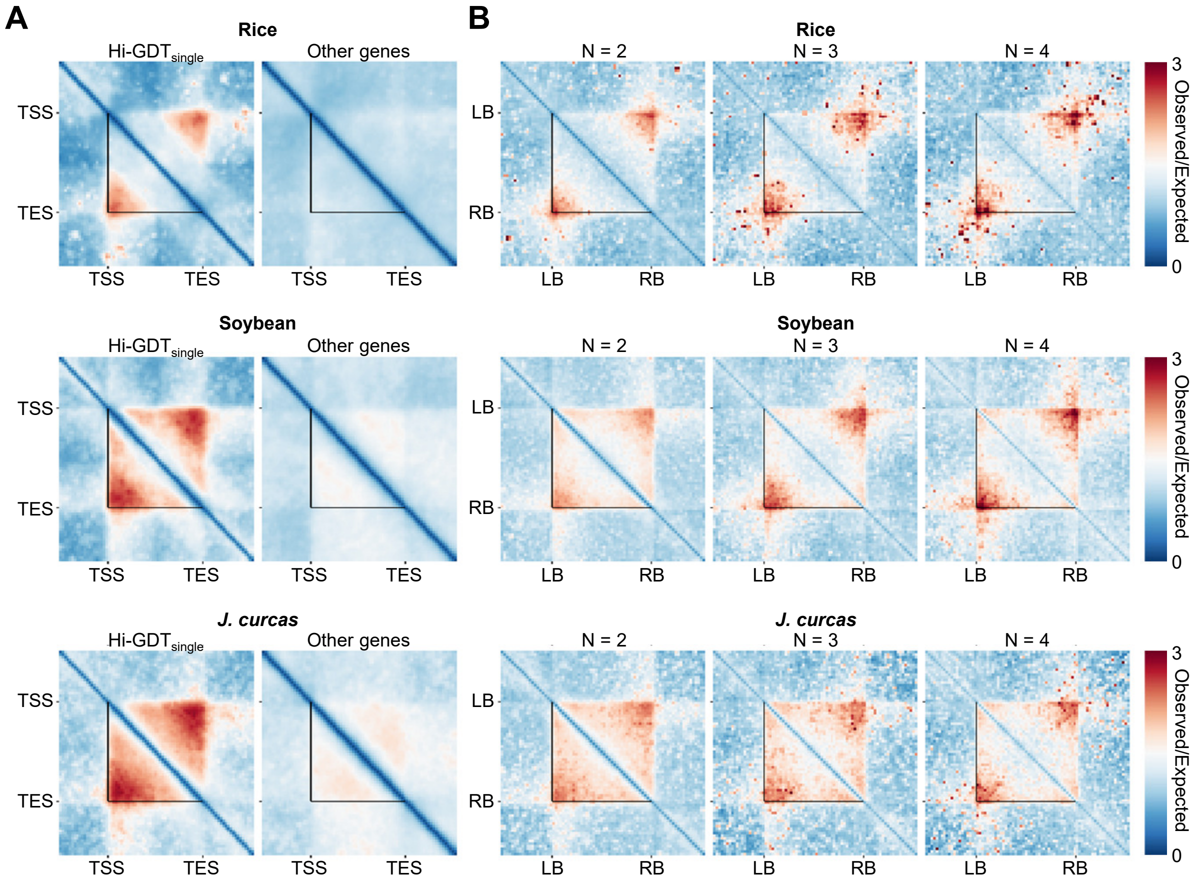

Supplementary Figure S14. Gene domains identified by Hi-GDT in various plant species.
(**A**) Pile-up images of Hi-C matrices for single-gene domains identified by Hi-GDT_{single} in rice, soybean, and *Jatropha curcas* (*J. curcas*). Pile-up images of genes with single-gene domains (Hi-GDT_{single}) and without single-gene domains (other genes) at 250-bp resolution are shown. Black lines indicate gene borders in each species. (**B**) Pile-up images of Hi-C matrices for identified multigene domains in rice, soybean, and *J. curcas* based on the number of constituent genes. Black lines indicate the boundaries of identified multigene domains.

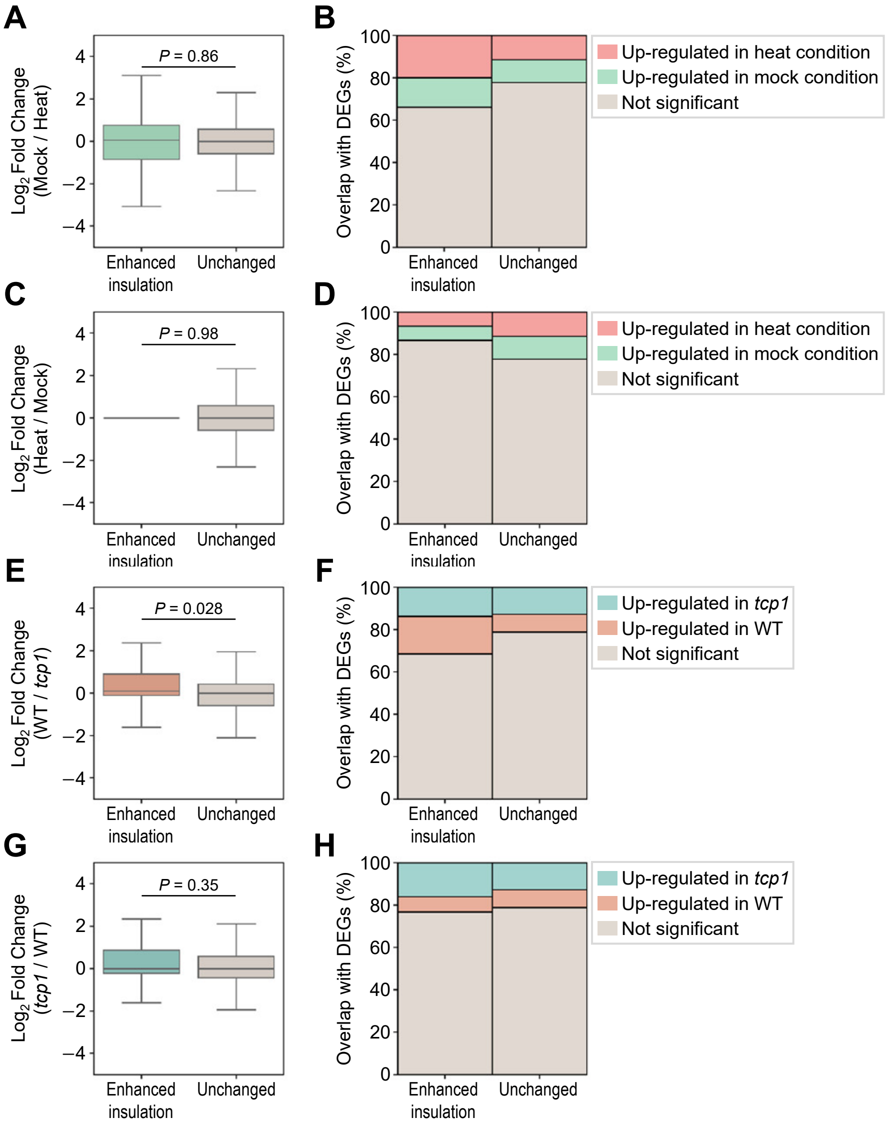

Supplementary Figure S15. Identification of differentially-insulated single-gene domains between different Hi-C datasets from tomato and *Marchantia*.

(**A** and **B**) Changes in gene expression levels for single-gene domains with reduced insulation after heat shock treatment in tomato. Log_2(fold change) of expression levels (**A**) and the proportions of overlap with DEGs between mock and heat conditions (**B**) in each group are shown. (**C** and **D**) Changes in gene expression levels for single-gene domains with enhanced insulation after heat shock treatment in tomato. Log_2(fold change) of expression levels (**C**) and the proportions of overlap with DEGs between mock and heat conditions (**D**) in each group are shown. (**E** and **F**) Changes in gene expression levels for single-gene domains with enhanced insulation in WT compared to *tcp1* mutant in *Marchantia*. Log_2(fold change) of expression levels (**E**) and the proportions of overlap with DEGs between WT and *tcp1* mutant (**F**) in each group are shown. (**G** and **H**) Changes in gene expression levels for single-gene domains with enhanced insulation in *tcp1* compared to WT in *Marchantia*. Log_2(fold change) of expression levels (**G**) and the proportions of overlap with DEGs between WT and *tcp1* mutant (**H**) in each group are shown. In (**A**), (**C**), (**E**) and (**G**), *P*-values were calculated by two-sided Mann–Whitney U-tests.

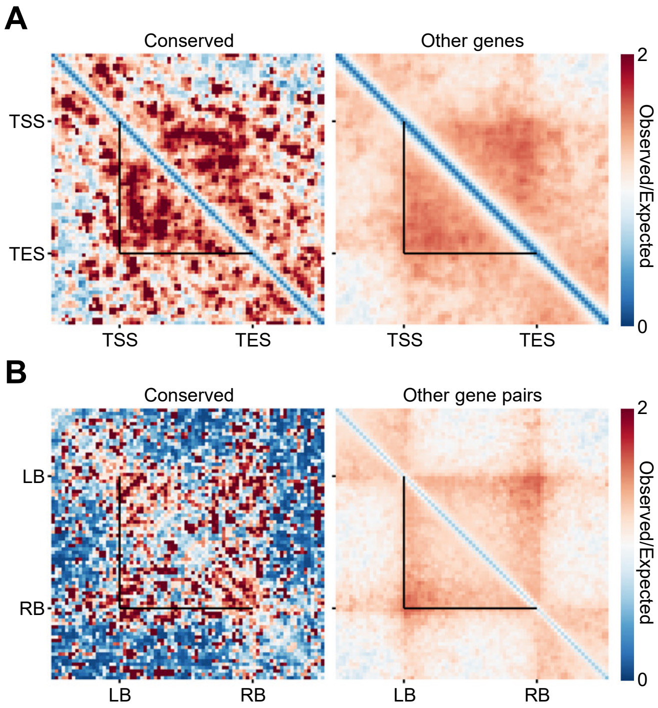

Supplementary Figure S16. Local contact domain structures of maize genes homologous to *Arabidopsis* single-gene domains and dual-gene domains

(**A**) Pile-up image of Hi-C matrices for maize genes homologous to *Arabidopsis* single-gene domains. All other genes in maize genome were used as control genes. Black lines indicate gene borders. (**B**) Pile-up image of Hi-C matrices for maize adjacent gene pairs homologous to *Arabidopsis* dual-gene domains. All other gene pairs in maize genome were used as control gene pairs. Black lines indicate the boundaries of identified adjacent gene pairs. Homologous genes or gene pairs within the colinear block of maize and *Arabidopsis* were used.

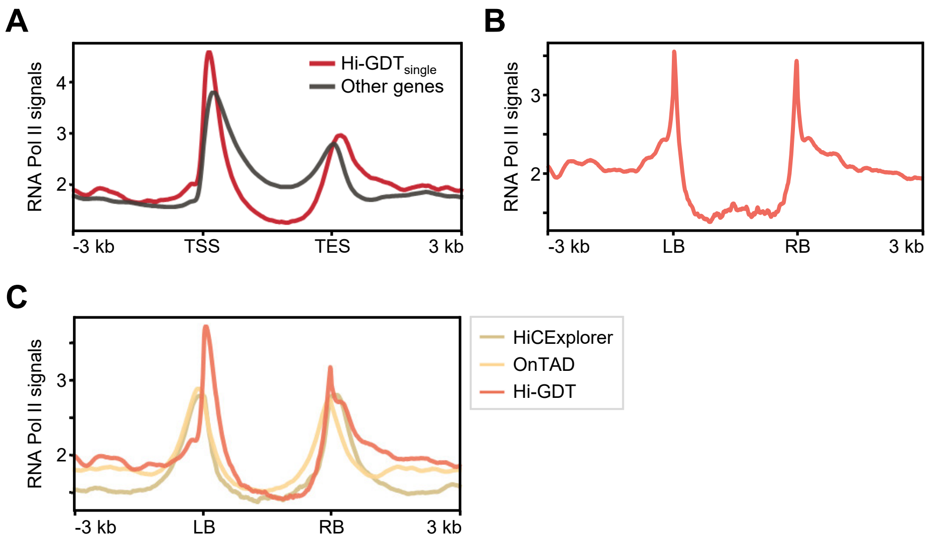

Supplementary Figure S17. Metaplots of RNA polymerase II (RNA Pol II) enrichment at the identified local contact domains.

(**A**, **B**) RNA Pol II enrichment level at the single-gene domains (**A**) and multigene domains (**B**) identified by Hi-GDT. In (**A**), single-gene domains identified by Hi-GDT$_{single}$ and remaining genes (other genes) are shown. (**C**) RNA Pol II enrichment level at the local contact domains identified by HiCExplorer, OnTAD, or Hi-GDT at 250 bp resolution. The metaplot of those identified by Arrowhead is not displayed due to the low number of identified domains.
