# Supplementary material for: Hi-GDT: A Hi-C-based 3D gene domain analysis tool for analyzing local chromatin contacts in plants
Source: Gigascience. 2025 Mar 21;14:giaf020. doi: 10.1093/gigascience/giaf020 (PMC11927400; doi:10.1093/gigascience/giaf020)
Supplement: giaf020_GIGA-D-24-00394_Revision_1 [file giaf020_giga-d-24-00394_revision_1.pdf]

GigaScience

Hi-GDT: A Hi-C-based 3D gene domain analysis tool for analyzing local chromatin contacts in plants.

--Manuscript Draft--

**Manuscript Number:**	GIGA-D-24-00394R1
**Full Title:**	Hi-GDT: A Hi-C-based 3D gene domain analysis tool for analyzing local chromatin contacts in plants.
**Article Type:**	Technical Note
**Funding Information:**	National Research Foundation of Korea (NRF-2022R1A2B5B02001266) — Prof. Pil Joon Seo
**Abstract:**	Background<br><br>Three-dimensional (3D) chromatin organization is emerging as a key factor in gene regulation in eukaryotes. Recent studies using high-resolution Hi-C analysis have explored fine-scale local chromatin contact domains in plants, as exemplified by the basic contact domains established at accessible gene border regions in Arabidopsis (Arabidopsis thaliana). However, we lack effective tools to identify these contact domains and examine their structural dynamics.<br><br>Results<br><br>We developed the Hi-C-based 3D Gene Domain analysis Tool (Hi-GDT) to identify fine-scale local chromatin contact domains in plants, with a particular focus on gene borders. Hi-GDT successfully identifies local contact domains, including single-gene and multigene domains, with high reproducibility. Hi-GDT can also be used to discover local contact domains that are differentially organized in association with differences in gene expression between tissue types, genotypes or in response to environmental stimuli.<br><br>Conclusions<br><br>Hi-GDT is a valuable tool for identifying genes regulated by dynamic 3D conformational changes, expanding our understanding of the structural and functional relevance of local 3D chromatin organization in plants. Hi-GDT is publicly available at https://github.com/CDL-HongwooLee/Hi-GDT.
**Corresponding Author:**	Pil Joon Seo<br>Seoul National University<br>Seoul, KOREA, REPUBLIC OF
**Corresponding Author Secondary Information:**	
**Corresponding Author's Institution:**	Seoul National University
**Corresponding Author's Secondary Institution:**	
**First Author:**	Hongwoo Lee
**First Author Secondary Information:**	
**Order of Authors:**	Hongwoo Lee<br>Pil Joon Seo
**Order of Authors Secondary Information:**	
**Response to Reviewers:**	Responses to Editorial Comments<br>Please register any new software application in the bio.tools and SciCrunch.org databases to receive RRID (Research Resource Identification Initiative ID) and biotoolsID identifiers, and include these in your manuscript. Computational workflows should be registered in workflowhub.eu and the DOIs cited in the relevant places in the

Powered by Editorial Manager® and ProduXion Manager® from Aries Systems Corporation

manuscript. These will facilitate tracking, reproducibility and re-use of your tool.
(RESPONSE) We have provided relevant information for your requests.

Responses to Reviewer 1

1. In Figure 2A, the comparison method OnTAD identifies a significantly higher number of local contact domains at different resolutions (250 bp, 500 bp, 1000 bp) than Hi-GDT. It would be helpful to include an interpretation of these differences in detection, as well as an explanation of how they might impact downstream analyses.
(RESPONSE) Thank you for the suggestion. Since the number of identified domains is highly dependent on the statistical cutoff of each tool, it would be appropriate to examine whether each tool can identify a sufficient number of contact domains at high resolution and the identification is reproducible. The higher number of identified domains by OnTAD (An et al., 2019) is likely derived from its specialized logic, which is optimized to detect hierarchical (nested) domain structures. As recommended, we have added relevant descriptions in the Discussion section of the revised manuscript. In addition, we implemented OnTAD with a higher cutoff value of 0.2 (default = 0.1) to reduce the number of identified contact domains (Figure R1). However, the Jaccard index was still comparable to the previous results, suggesting that reproducibility is likely independent of the number of identified contact domains (Figure R2). We carefully concluded that Hi-GDT outperforms existing tools, including OnTAD that has intrinsically low reproducibility in identifying fine-scale contact domains.

Figure R1. The number of contact domains identified by OnTAD using a cutoff value of 0.2

Figure R2. The Jaccard index of contact domains identified by OnTAD using a cutoff value of 0.2

2. The section on "Identification of differentially insulated single-gene domains associated with transcriptional activity depending on tissue type" notes that Hi-GDTdiff performed well on both the wild type (Col-0) and the triple mutant (bmi1a bmi1b bmi1c). However, the manuscript does not include figures or tables to support this claim. Including visual data (e.g., charts or tables) would strengthen this section and provide clarity on the results.
(RESPONSE) We indicated figures with supporting results (Supplementary Figure S10 and Supplementary Table S2).

3. While Hi-GDT is shown to perform well across different plant species (e.g., Arabidopsis, tomato, maize, and Marchantia), it is unclear whether the model was trained separately for each species or designed as a generalized model. Additional details on training strategy and cross-species generalization would provide valuable insights into its adaptability and robustness.
(RESPONSE) Please note that we did not employ machine learning models but design rule-based algorithms. Based on our previous findings that gene domain structures are widely conserved across various plant species (Lee and Seo, 2023), we designed Hi-GDT to recognize the common feature of gene domains from Hi-C matrices, including enhanced intra-domain contacts within the boundaries of TSS and/or TES. We tried to further validate cross-species generalization by applying Hi-GDT to additional plant genomes, such as in rice, soybean, and Jatropha curcas (Supplementary Figure S14). We also provided additional explanations in the revised manuscript for better understanding.

4. Although the performance of Hi-GDT is notable, a detailed framework diagram of the model would enhance reader comprehension. This could clarify the structural design of Hi-GDT and facilitate reproducibility for future research.
(RESPONSE) We have revised Figure 1 and also added relevant information in our github page (https://github.com/CDL-HongwooLee/Hi-GDT) to improve clarity.

5. Given the growing body of research on predicting structural units and regulatory elements within three-dimensional genomes (e.g., PMID: 35997565P, PMID: 36161334, MID: 37843994, PMID: 37402191, PMID: 38485768, PMID: 39085477), it

Powered by Editorial Manager® and ProduXion Manager® from Aries Systems Corporation

would be beneficial for the authors to reference additional recent studies in this area. Doing so would strengthen the context of the work, highlight its relevance to current advancements, and potentially broaden the article's readership.
(RESPONSE) We have cited relevant papers to further support our claims.

Responses to Reviewer 2
1. Through your manuscript, I noticed that there are certain differences between the three-dimensional genome organization of plants and animals. For example, you mentioned that "The CTCF core architectural protein is not conserved across various plant species…lack strong interactions at anchor sites." Additionally, your results demonstrate that your method outperforms existing methods originally developed for analyzing animal data. I suggest that you further analyze the reasons why your method is particularly well-suited for plant three-dimensional genome analysis. Specifically, does your method explicitly account for the differences between plant and animal genome organization, which might have contributed to its superior performance?
(RESPONSE) The superior performance of Hi-GDT stems from addressing two major challenges (Supplemental Figure S4A): (1) the weak interactions at contact domain boundaries due to the lack of CTCFs in plants; (2) high noise in high-resolution analysis (e.g. 250 or 500 bp). The semi-targeted approach that uses gene border regions (TSS and TES) as boundaries of local contact domains (Lee and Seo, 2023; Sun et al., 2024; Zhang et al., 2024), rather than random genome-wide search, improves accuracy and sensitivity in identifying gene domains from noisy Hi-C data. We have added relevant descriptions in the Discussion section.

Lee H, Seo Pil J. Accessible gene borders establish a core structural unit for chromatin architecture in Arabidopsis. Nucleic Acids Res. 2023;51:10261-77. doi:10.1093/nar/gkad710.
Sun L, Zhou J, Xu X, et al. Mapping nucleosome-resolution chromatin organization and enhancer-promoter loops in plants using Micro-C-XL. Nat Commun. 2024;15:35. doi:10.1038/s41467-023-44347-z.
Zhang Y, Dong Q, Wang Z, et al. A fine-scale Arabidopsis chromatin landscape reveals chromatin conformation-associated transcriptional dynamics. Nat Commun. 2024;15:3253. doi:10.1038/s41467-024-47678-7.

2. There is a method called HiCDB(HiCDB: a sensitive and robust method for detecting contact domain boundaries) that is designed for detecting contact domains. Similar to your approach, HiCDB considers two key characteristics: the self-association and the insulation properties of contact domains. Moreover, their results demonstrate that HiCDB is effective in precisely detecting smaller-scale contact domains and have high reproducibility. Therefore, I recommend including a comparative experiment between HiCDB and your method.
(RESPONSE) Thank you very much for your valuable suggestion. However, we noticed that HiCDB effectively identifies the ‘boundary’ of local contact domains, rather than the entire contact domain structure. Our benchmark analysis evaluates several features of contact domains identified by Hi-GDT and other domain callers, including the number and size of contact domains as well as the reproducibility of contact domain identification. These analyses do not align with the output results of HiCDB, and the direct comparison is not feasible. Please understand our reasoning.

3. I have noticed a concept similar to gene domain, namely gene-body-associating domains (GADs), which can be found in the paper " tagHi-C reveals 3D chromatin architecture dynamics during mouse hematopoiesis". GADs are structurally independent of CTCF binding and highly correlated with gene expression, active histone modifications (H3K4me1), and RNA Pol II binding at the gene body. Concurrently, you have mentioned that "the concerted action of transcription factors and paused RNA polymerase II (RNA Pol II) at gene borders plays a crucial role in shaping local chromatin structure in Arabidopsis." Thus, I believe that it would be beneficial to include the distributions of RNA Pol II signals at the gene domains identified by different methods via an aggregation plot. This addition would provide a more comprehensive demonstration of the accuracy of Hi-GDT in detecting gene domains.
(RESPONSE) We provided a metaplot analysis showing the RNA Pol II enrichment around the local contact domains identified by Hi-GDT or other domain callers. The data were newly included as Supplementary Figure S15.

Powered by Editorial Manager® and ProduXion Manager® from Aries Systems Corporation

	4. It is recommended to expand the analysis and validation experiments of Hi-GDT to other publicly available datasets.<br>(RESPONSE) We applied Hi-GDT to additional plant Hi-C data including rice, soybean, and Jatropha curcas, and found that Hi-GDT successfully identified gene domain structures in such plant species. The data were newly included as Supplementary Figure S17.
Additional Information:	
Question	Response
Are you submitting this manuscript to a special series or article collection?	No
**Experimental design and statistics**<br><br>Full details of the experimental design and statistical methods used should be given in the Methods section, as detailed in our Minimum Standards Reporting Checklist. Information essential to interpreting the data presented should be made available in the figure legends.<br><br>Have you included all the information requested in your manuscript?	Yes
**Resources**<br><br>A description of all resources used, including antibodies, cell lines, animals and software tools, with enough information to allow them to be uniquely identified, should be included in the Methods section. Authors are strongly encouraged to cite Research Resource Identifiers (RRIDs) for antibodies, model organisms and tools, where possible.<br><br>Have you included the information requested as detailed in our Minimum Standards Reporting Checklist?	Yes
**Availability of data and materials**<br><br>All datasets and code on which the conclusions of the paper rely must be either included in your submission or deposited in publicly available repositories	Yes

Powered by Editorial Manager® and ProduXion Manager® from Aries Systems Corporation

(where available and ethically appropriate), referencing such data using a unique identifier in the references and in the “Availability of Data and Materials” section of your manuscript.<br><br>Have you have met the above requirement as detailed in our [Minimum Standards Reporting Checklist]?	
GigaScience has policies and guidelines in place for the use of generative AI-writing tools such as ChatGPT. If you have used such writing tools to assist with writing the manuscript this must be declared and cited in the text. Authors should not list AI-writing tools and other AI-assisted technologies as an author or co-author and should acknowledge that they are fully responsible for text generated or refined by AI-writing tools.<br><br>A summary of use (particularly in the introduction or among methods) needs to be included at the end of the paper, and the outputs should also be included as a supplementary file hosted in GigaDB or other open repositories. Please read our guidelines for more information. <br>By submitting to GigaScience, you are aware of the journal's AI-writing tools policy, and if you have declared use of such tools below, you have acknowledged this where appropriate in your manuscript and have made a summary of use and outputs available. <br><br>**AI-assisted writing tools have been used in the preparation of this manuscript?<br>** 	No

Powered by Editorial Manager® and ProduXion Manager® from Aries Systems Corporation

Manuscript Click here to access/download;Manuscript;394-Manuscript-revision-PJS.docx

Hi-GDT: A Hi-C-based 3D gene domain analysis tool for analyzing local chromatin contacts in plants

Hongwoo Lee[1] and Pil Joon Seo[1,2,*]

[1]Department of Chemistry, Seoul National University, Seoul 08826, Korea

[2]Plant Genomics and Breeding Institute, Seoul National University, Seoul 08826, Korea

[*]To whom correspondence should be addressed: pjseo1@snu.ac.kr

ORCID iDs:

Hongwoo Lee [0000-0002-9339-2757]; Pil Joon Seo [0000-0002-5499-3138];

Abstract

Background

Three-dimensional (3D) chromatin organization is emerging as a key factor in gene regulation in eukaryotes. Recent studies using high-resolution Hi-C analysis have explored fine-scale local chromatin contact domains in plants, as exemplified by the basic contact domains established at accessible gene border regions in *Arabidopsis* (*Arabidopsis thaliana*). However, we lack effective tools to identify these contact domains and examine their structural dynamics.

Results

We developed the Hi-C-based 3D Gene Domain analysis Tool (Hi-GDT) to identify fine-scale local chromatin contact domains in plants, with a particular focus on gene borders. Hi-GDT successfully identifies local contact domains, including single-gene and multigene domains, with high reproducibility. Hi-GDT can also be used to discover local contact domains that are differentially organized in association with differences in gene expression between tissue types, genotypes or in response to environmental stimuli.

Conclusions

Hi-GDT is a valuable tool for identifying genes regulated by dynamic 3D conformational changes, expanding our understanding of the structural and functional relevance of local 3D chromatin organization in plants. Hi-GDT is publicly available at https://github.com/CDL-HongwooLee/Hi-GDT.

Key words: Arabidopsis, Hi-C, fine-scale contact domain, gene domain, gene domain analysis tool.

Key Points

- We developed the Hi-C-based 3D Gene Domain analysis Tool (Hi-GDT) to identify fine-scale local chromatin contact domains.
- Hi-GDT successfully identifies local contact domains, including single-gene and multigene domains, with high reproducibility.
- Hi-GDT also identifies 3D contact domains that are dynamically organized based on their transcriptional states.

Introduction

Three-dimensional (3D) chromatin organization is important for the proper structural arrangement of the genome and for controlling gene transcription [1-7]. Eukaryotic genomes are organized into structural units known as topologically associating domains (TADs), which exhibit strong self-interaction and thus appear as triangular shapes in 3D chromatin contact maps [8-10]. TADs can be classified into two types depending on the presence or absence of strong contacts at anchor sites (shown as corner dots in 3D Hi-C chromatin contact maps) [11-15]. 3D contact domains with strong contacts at anchor sites (called loop domains) are established by cohesin and chromatin insulator proteins, including CCCTC-binding factor (CTCF), through a loop-extrusion mechanism [16-18]. By contrast, 3D contact domains without strong contacts at anchor sites (called compartment domains) form independently of CTCF and cohesin and are instead associated with local A/B compartments and epigenetic states [11-13, 15, 19].

The CTCF core architectural protein is not conserved across various plant species [20]. Consistent with this finding, a majority of plant 3D chromatin domains lack strong interactions at anchor sites. Instead, their formation is likely to be dependent on epigenetic states, as observed in tomato (*Solanum lycopersicum*), rice (*Oryza sativa*), sorghum (*Sorghum bicolor*), and foxtail millet (*Setaria italica*) [21-24]. Plants with large genomes, such as maize (*Zea mays*), cotton (*Gossypium hirsutum*), pepper, and wheat (*Triticum aestivum*), also frequently contain 3D chromatin domains demarcated by gene-to-gene loops, which are enriched with active chromatin marks [25-29], suggesting that epigenetic states are critical for organizing the 3D conformation of chromatin in plants. Although the molecular mechanisms of 3D chromatin domain formation vary, the sizes of such domains in these plant species are comparable to those of TADs in mammals. Therefore, conventional TAD analysis tools have been reasonably

applied to identify and analyze large 3D chromatin domains in plants.

The compartment domains in plant species with small genomes have recently been explored. Using ultra-deep Hi-C sequencing and advanced Hi-C-based techniques, fine-scale local chromatin domains have been identified in the *Arabidopsis* (*Arabidopsis thaliana*) genome, which are intimately associated with epigenetic states [30-33]. Notably, representative local chromatin domains (called gene domains) are organized at gene border regions with high chromatin accessibility in *Arabidopsis* [34]. These contact domains form at the single-gene scale through a process driven by self-interaction between transcriptional start sites (TSSs) and transcriptional end sites (TESs). They also form at chromatin regions spanning a series of multiple genes with high chromatin accessibility at their borders [34]. Although gene domains are conserved across diverse plant species, including tomato, maize and *Marchantia polymorpha* (*Marchantia*), the functional importance of these fine-scale local chromatin domains in plants has received little attention. One study examined the *Arabidopsis* biosynthetic gene clusters of the specialized metabolites thalianol and marneral, finding that each biosynthetic gene cluster is located in a 3D chromatin domain whose structure is likely reorganized in response to internal and/or external cues to ensure the transcriptional coordination of the associated genes [35]. Otherwise, this area of research has been limited, largely due to the lack of proper analysis tools for such contact domains. In particular, most existing contact domain callers were originally designed for analyzing animal TADs. However, given that gene domains are much smaller than conventional TADs or compartment domains and exhibit relatively low contact frequencies between anchor sites, new tools are needed that can analyze the fine-scale chromatin domains with high sensitivity and precision from high-resolution 3D contact maps.

In this study, we describe the Hi-C-based Gene Domain analysis Tool (Hi-GDT), which

we designed to identify 3D contact domains with a focus on gene border regions, enabling more stringent fine-scale gene domain analysis. This tool successfully identified local contact domains, including single-gene and multigene domains, across various Hi-C datasets from *Arabidopsis*, tomato, maize and *Marchantia*. Hi-GDT also identified genes with differential 3D contact domains that are dynamically organized based on their transcriptional states in different tissue types, genotypes, and in response to environmental stimuli. Hi-GDT serves as a valuable tool for analyzing fine-scale local chromatin organization in plants.

Materials and Methods

Plant materials

Arabidopsis (*Arabidopsis thaliana*) accession Columbia (Col-0) was grown at 23°C under long-day conditions (16 h light/8 h dark) using white fluorescent lamps (120 μmol photons m^{-2} s^{-1}). For callus induction, the third and fourth leaves from 2-week-old seedlings were excised and incubated on callus-inducing medium (CIM; Murashige and Skoog medium supplemented with 0.5 μg/mL 2,4-D and 0.05 μg/mL kinetin) for 7 days. The callus tissues were selectively harvested for Hi-C analyses.

RNA-sequencing experiments

Leaf explant samples were harvested immediately after excision (0 day), and leaf explant–derived callus samples were harvested at 7 days after incubation on CIM. RNA libraries were constructed with 1 μg of total RNA using a TruSeq stranded mRNA library kit (Illumina, USA) as previously described [36].

Hi-C experiments and processing

Leaf explant–derived callus samples were harvested at 7 days after incubation on CIM. Hi-C data for callus tissue were generated using an Arima-HiC kit (Arima Genomics, USA) according to the manufacturer's protocol, followed by next-generation sequencing performed by Macrogen Inc. (Seoul, South Korea). The publicly available Hi-C sequencing files were downloaded from the NCBI Sequence Read Archive (SRA; Supplementary Table S1) [24, 37-42]. Fastq files were extracted from the SRA files and processed using Juicer (v2.0) (RRID:SCR_017226) [43] with the corresponding reference genome (TAIR10 for *Arabidopsis*, M82v1.0 for tomato, B73v5 for maize and MpTak1v5 for *Marchantia*). Mapped reads were filtered with a mapping quality cutoff value of 30 in *Arabidopsis*, tomato and *Marchantia*, whereas no mapping quality cutoff was applied in maize. SCALE normalization was applied to the Hi-C contact matrices to correct for sequencing bias. The compressed binary files (.hic) generated by Juicer were used as inputs for Hi-GDT.

Single-gene domain analysis by Hi-GDT

Hi-GDT identifies single-gene domains by comparing intragenic contact frequencies with those in designated control regions. Given the relatively high contact frequencies at TSS-TES anchor sites, the intragenic target region near the anchor site is visualized as a triangle in Figure 1B. The size of this region was defined as half the length of the corresponding gene. The control regions were set to be equidistant with a target region from the diagonal axis of a Hi-C contact map, including intragenic regions at one side of the anchor (Figure 1B). The distance-normalized intragenic contact frequencies (OE values; observed contact frequency/expected contact frequency) within both intragenic and control regions were collected and tested using the Mann–Whitney U-test. Genes with significantly higher contact frequencies within

intragenic regions compared to the surrounding control regions were defined as single-gene domains. Benjamini-Hochberg corrected P-value (Q-value) cutoff of 0.05 was applied for the identification of single-gene domains from the Hi-C datasets at 250-bp resolution.

For differential single-gene domain analysis, the OE values within (1) the surrounding 2-kb regions and (2) the gene body regions of individual genes from two different Hi-C datasets were collected. The OE values within the surrounding regions were subjected to a Wilcoxon signed-rank test to identify genes with differential (Q-value < 0.05) surrounding contact frequencies between two Hi-C datasets. In addition, a cutoff value of 0.1 for mean fold change of OE values was used to filter the differentially insulated genes that exhibited both increased contact frequencies within gene body regions and decreased contact frequencies within surrounding 2-kb regions. For example, fold change filters of OE values to identify single-gene domains with enhanced insulation under certain treatment condition are applied as below.

$$\frac{Gene\ \ body\ \ OE_{control}}{Gene\ \ body\ \ OE_{treat}} < (1-0.1), \qquad \frac{Surrounding\ \ 2\ \ kb\ \ OE_{treat}}{Surrounding\ \ 2\ \ kb\ \ OE_{control}} < (1-0.1)$$

Multigene domain analysis by Hi-GDT

The identification of multigene domains involved two types of comparisons between intra-domain regions and outside control regions: (1) comparisons of gene-to-gene contact frequencies at the domain boundaries with those in outside control regions, and (2) comparisons of intra-domain contact frequencies at each domain boundary with those in respective control regions (Figure 1C; Supplementary Figure S1). The control and target regions were set to be equidistant from the diagonal axis of a Hi-C contact map. Mann–Whitney U-tests were employed to compare OE values between pairs of target and control regions. A multigene domain was defined as a cluster of adjacent genes that exhibited significantly higher OE values within target regions compared to all their respective control regions.

To analyze multigene domain dynamics, multigene domains were identified at 500-bp resolution. Multigene domains were identified based on *Q*-value cutoffs of 0.05. Dual- and triple-gene domains identified in each specific tissue were compared to analyze the structural dynamics of multigene domains. Genes forming dual-gene domains with a TSS at one boundary, without forming single-gene domains, were used to analyze changes in gene expression.

Identification of 3D contact domains using existing domain callers

Domain calling with Arrowhead [43], HiCExplorer (RRID:SCR_022111) [44-46], and OnTAD [47] was conducted at 250-, 500-, and 1,000-bp resolution for benchmarking with Hi-GDT. The parameter '-m 100 -k SCALE' was used for Arrowhead, and the parameter '-penalty 0.1 -minsz 3 -maxsz 200 -hic_norm SCALE' was used for OnTAD. For HiCExplorer, SCALE-normalized .hic files were converted to .h5 format using the hicConvertFormat command, and domain calling was conducted using hicFindTADs with the parameter '--correctForMultipleTesting fdr'.

Analysis of A and B compartments

To define the local A/B compartment, Pearson's correlation matrix of the SCALE-normalized Hi-C contact map at 750-bp resolution was obtained using Juicer Tools [43]. The first eigenvector of the correlation matrix (compartment eigenvector) was calculated using numpy.linalg.eig (RRID:SCR_008633) [48]. For single-gene domain analysis, compartment eigenvector values were measured at the TSS, the center region of the gene body, and the TES. Compartment eigenvector values > 0 were assigned to the A compartment, and values < 0 were assigned to the B compartment.

Metaplot analysis

Metaplot analysis for directionality index (DI) score, chromatin accessibility, and compartment eigenvector was performed using deepTools (RRID:SCR_016366) [49]. The DI score with a 5-kb window was calculated at 250-bp resolution as described previously [8]. The processed BigWig file of Assay for Transposase-Accessible Chromatin (ATAC)-seq data was downloaded from the Plant Chromatin State Database (PCSD) [50, 51]. The compartment eigenvector at 750-bp resolution was converted to a BigWig file and used as input for metaplot analysis. For single-gene domain analysis, protein-coding genes without a single-gene domain structure were used for comparison.

Pile-up analysis

The SCALE-normalized OE values of Hi-C contact matrices at 250-bp resolution were used for pile-up analysis. The OE values within identified contact domains and their surrounding regions (0.5× domain size) were obtained with a bin size of 10 bp and subsequently resized to an 80 × 80 matrix using bicubic interpolation. For single-gene domain analysis, the images of (−)-stranded genes were flipped to synchronize gene orientation. Pile-up analysis was conducted using PileUpDomain.py [52].

Analysis of chromatin state

Information about the 26 chromatin states was obtained from a previous report [53]. For single-gene domain analysis, chromatin states that overlapped with the TSS, the center of the gene body, and the TES were measured. For multigene domain analysis, chromatin states overlapping with the centers of genes located at multigene domain borders, at internal regions of multigene domains, and at outside regions of multigene domains were investigated. Genes

forming single-gene domains were excluded from multigene domain analysis. To investigate the similarity of chromatin states between gene pairs in quadruple-gene domains, both genes in chromatin at the same states (constitutive heterochromatin [H], facultative heterochromatin [F], intergenic [I], and euchromatin [E]) were examined.

RNA-sequencing analysis

The RNA-sequencing reads used in this study were downloaded from the SRA database (Supplementary Table S1) [21, 24, 38, 42, 54]. The reads were mapped to the corresponding reference genome using STAR (v2.7.10a) (RRID:SCR_004463) [55] with the parameter '--peOverlapNbasesMin 12 --peOverlapMMp 0.1 --twopassMode Basic'. Transcript abundance was quantified using RSEM (v1.3.1) (RRID:SCR_000262) [56]. Differentially expressed genes (DEGs) and fold change values were identified by Deseq2 (v1.34.0) (RRID:SCR_015687) [57] with a threshold of $\log_2$(fold change) > 1 and *Q*-value < 0.05. Log_2(transcripts per million [TPM] + 1) values were used to quantify gene expression levels.

Calculation of Tau score

Tau score, a measure of tissue-specific gene expression, was calculated as previously described [58]. The expression levels of genes from various *Arabidopsis* tissues were downloaded from a previous report [59]. The corrected reads per kilobase of transcript per million mapped reads (cRPKM) values were used to calculate the Tau score.

Gene Ontology (GO) analysis

GO analysis was performed using AmiGO2 powered by PANTHER (RRID:SCR_004869) [60-62]. The top 10 or 20 significantly enriched GO terms with *P*-value < 0.05 and number of gene

hits > 1 were visualized.

Colinear block analysis

Colinear blocks were identified using a pipeline that utilizes MCScanX (RRID:SCR_022067) [63]. Briefly, inter-species BLASTP analysis (RRID:SCR_001010) [64] was conducted between the *Arabidopsis* and tomato genomes, and between the *Arabidopsis* and maize genomes. A pairwise BLASTP was conducted by switching the reference genome, and the best five hits with an E-value cutoff of 1×10^{-10} were kept for the detection of colinear blocks. Colinear blocks between two different species were defined using MCScanX [65] with the default parameters. Among the genes within the colinear blocks in the tomato and maize genomes, those homologous to *Arabidopsis* single-gene domains, and adjacent gene pairs homologous to *Arabidopsis* dual-gene domains were collected and visualized.

Data visualization

All heatmaps and one-dimensional plots were generated with matplotlib (RRID:SCR_008624) [66] and seaborn (RRID:SCR_018132) [67]. Hi-C maps were visualized using JuiceBox (RRID:SCR_021172) [43].

Results

Development of Hi-GDT to analyze local contact domain structures

The discovery of gene domains in plants motivated us to develop Hi-GDT, a specialized tool for identifying fine-scale gene domain structures [34]. Since gene domains are prevalent in *Arabidopsis*, which have been analyzed with various high-resolution Hi-C datasets, we

employed high-resolution *Arabidopsis* Hi-C data to validate the performance of our tool [38]. We design a rule-based algorithm considering key features of gene domains, which include self-interaction patterns and strong insulation at gene borders in Hi-C contact maps, to identify such domains (Hi-GDT_{single} for single-gene domains and Hi-GDT_{multi} for multigene domains) (Figure 1A). For further analysis of the structural dynamics of single-gene domains, we also developed Hi-GDT_{diff} as a means to identify single-gene domains showing the 3D structural changes that are associated with differential gene expression in different tissue types, genotypes or in response to environmental stimuli.

Given that single-gene domains display a self-interaction pattern with boundaries of TSSs and TESs that have high chromatin accessibility [34], we compared the distance-normalized intragenic contact frequencies (observed contact frequency/expected contact frequency, or OE) within a genic region defined by a TSS and TES with those of control regions outside genic regions to identify single-gene domains (Figure 1B). For this comparison, we extracted OE values from the triangular region of a Hi-C contact map whose size was defined based on gene length (0.5× gene length). The target region (red triangle in Figure 1B) was defined as the intragenic region near gene borders, whereas the control regions (green triangles) were determined based on two criteria: (1) the distance from the diagonal axis of a Hi-C contact map should be equal to that of the target region, to minimize distance-dependent bias; and (2) they should consist of interacting chromatin pairs, with one located outside and the other inside a gene body. We conducted a Mann–Whitney U-test to compare OE values between target and control regions. Genes with significantly higher OE values in intragenic regions compared to those in both control regions were considered to form single-gene domains (Figure 1B).

To identify multigene domains, we conducted two distinct comparisons of OE values between target (red squares) and control regions (green and yellow squares) that were

equidistant from the diagonal axis of a Hi-C contact map (Figure 1C; Supplementary Figure S1): (1) OE values of gene-to-gene contact sites located at multigene domain boundaries were compared to those within the external control regions; and (2) OE values at domain boundaries were compared to those in the two external control regions positioned in opposite directions (Figure 1C; Supplementary Figure S1). Adjacent gene sets that passed all of these statistical comparisons were annotated as multigene domains. In summary, Hi-GDT identified single-gene and multigene domains based on image analysis of Hi-C contact maps.

Hi-GDT is suitable for analyzing local contact domains in *Arabidopsis*

Since Hi-GDT was designed specifically for gene domain identification, we examined its possible advantages over conventional TAD callers, including Arrowhead [43], HiCExplorer [44-46], and OnTAD [47], in terms of the fine-scale identification of local contact domains in *Arabidopsis*. Hi-GDT identified a substantial number of local contact domains: 13,049 at 250-bp resolution and 10,089 at 500-bp resolution. These numbers were higher than the numbers of local contact domains identified by Arrowhead and HiCExplorer (Figure 2A). The majority of contact domains identified by Hi-GDT were small in terms of size (<10 kb; Figure 2B; Supplementary Figure S2A). Notably, conventional domain callers generally predicted local contact domains at gene border regions (Supplementary Figure S3), in agreement with the local contact domains formed mainly at gene boundaries in *Arabidopsis* [34]. We conducted pile-up analysis of the local contact domains identified by each domain caller to estimate their collective structural features. The contact domains identified by Hi-GDT and Arrowhead showed stronger contact domain structures that were more obvious than those identified by HiCExplorer and OnTAD across all tested resolutions, from 250 to 1,000 bp (Figure 2C; Supplementary Figure S2B).

We also assessed the reproducibility of local contact domain identification by each domain caller by comparing the contact domains identified from technical replicates with 80% subsampling of a merged Hi-C dataset, biological replicates, or two Hi-C datasets from different batches. The reproducibility of the conventional domain callers for domain identification was low, ranging from 29% to 61% between technical replicates, 4% to 29% between biological replicates, and 1% to 10% between datasets from different batches (Supplementary Figure S4A), suggesting that conventional domain callers have a limited ability to identify fine-scale local contact domains. In comparison, Hi-GDT showed higher reproducibility (Supplementary Figure S4A). Since, unlike the other domain callers, Hi-GDT primarily focuses on pre-defined boundaries (such as TSSs and TESs), we reanalyzed the reproducibility of the conventional domain callers for gene domain identification by allowing the callers to adjust domain boundaries to coincide with gene border regions for a fair comparison. Although this increased the reproducibility of the conventional domain callers to some extent, Hi-GDT still exhibited the highest reproducibility score as well as Jaccard index, which represents the similarity between two groups (Figure 2D; Supplementary Figure S4B). Taken together, these results indicate that Hi-GDT is highly optimized for fine-scale local contact domain identification in *Arabidopsis* with high reproducibility.

Validation of single-gene domains identified by Hi-GDT$_{single}$

We investigated whether the single-gene domains identified by Hi-GDT$_{single}$ showed key characteristics of previously reported single-gene domains [34]. Pile-up analysis and metagene analysis of directionality index (DI) revealed that the Hi-GDT$_{single}$-identified single-gene domains at 250-bp resolution were insulated at the TSS and TES regions (Figure 3A, B). Furthermore, the border regions of these Hi-GDT$_{single}$-identified gene domains had highly

accessible chromatin conformations (Figure 3C), as observed in previously reported single-gene domains [34]. We then analyzed the compartment eigenvector at high resolution (750 bp) to explore the distribution of local A/B compartments in these single-gene domains. The compartment eigenvector in Hi-GDT_{single}-identified gene domains showed positive values (A compartments) at gene domain borders but negative values (B compartments) inside gene domains (Figure 3D). Consistent with the local compartment distribution of single-gene domains in *Arabidopsis* [34], the Hi-GDT_{single}-identified gene domains predominantly had an A-B-A compartment pattern: A compartments at TSS and TES regions and B compartments at gene bodies (Figure 3D, E). This observation is also in line with the finding that active TSSs are primarily found within local A compartments in both plants and animals [34, 68].

We further explored the association of Hi-GDT_{single}-identified gene domains with transcriptional states. In general, genes with local contact domain structures had higher expression levels than control genes without local contact domains, implying that transcriptionally active genes are likely to form single-gene domains (Figure 3F). We also analyzed the chromatin states of Hi-GDT_{single}-identified gene domains based on the 26 previously reported chromatin states, including 6 constitutive heterochromatin states (H1–H6), 6 facultative heterochromatin states (F1–F6), 3 intergenic states with the highest chromatin accessibility (I1–I3), and 11 euchromatin states (E1–E11) [53]. Genes with local contact domain structures were frequently located in euchromatin, whereas genes without these structures were more frequently located in facultative heterochromatin (Figure 3G), indicating that Hi-GDT_{single} successfully identifies gene domains in transcriptionally active states.

Validation of multigene domains identified by Hi-GDT_{multi}

In addition to identifying single-gene domains, Hi-GDT also enabled us to identify multigene

domains by recognizing their insulating gene borders (Figure 1C). Using Hi-GDT$_{multi}$, we identified 6,420 multigene domains at 500-bp resolution, with a median size of 11,550 bp comprising an average of 3.66 genes in *Arabidopsis* (Figure 4A, B). Numerous multigene domains with distinct domain boundaries were observed in Hi-C contact maps (Figure 4C; Supplementary Figure S5A). Pile-up images of Hi-GDT$_{multi}$-identified contact domains also revealed strong domain boundaries, regardless of the number of constituent genes (Figure 4D). Genes located at the borders of multigene domain were predominantly oriented in a convergent direction, leading to highly accessible TSS–TSS interactions (Supplementary Figure S5B-D), which is consistent with previous findings [34].

Similar to single-gene domains, the borders of Hi-GDT$_{multi}$-identified multigene domains exhibited high chromatin accessibility (Figure 4E) and were usually located in the local A compartment, leading to strong A-B-A compartmentalization (Figure 4F). Consistent with this finding, genes located at the borders of multigene domains resided in euchromatin regions with high transcriptional activity, whereas genes inside multigene domains were present in facultative heterochromatin, which usually has low transcriptional activity (Figure 4G, H; Supplementary Figure S6). Furthermore, pairs of genes located at the contact domain borders were coordinated to have active chromatin states in common, whereas series of internal genes within a multigene domain had similar transcriptionally repressive heterochromatic states (Figure 4I). These results suggest that Hi-GDT can reliably identify multigene domains in *Arabidopsis*.

Identification of differentially insulated single-gene domains associated with transcriptional activity depending on tissue type

Taking advantage of Hi-GDT$_{single}$, we analyzed independent Hi-C datasets and compared them

to examine the potential dynamics of single-gene domain structures. Our goal was to determine whether Hi-GDT_{single} is reliable across various resolutions and when using different Hi-C datasets. Despite the variation in the number of identified contact domains depending on the resolution (Supplementary Figure S7A), we obtained relatively consistent results at all resolutions examined: Hi-GDT_{single}-identified single-gene domains were characterized by strong anchors at the TSS-TES regions and transcriptionally active states (Figure 3A, F; Supplementary Figure S7B, C). We also confirmed the reliability of Hi-GDT_{single} for processing independent *Arabidopsis* Hi-C datasets obtained from different tissues or environmental conditions (Supplementary Figure S7D).

Given that TADs are closely linked with cellular identity in animals [69-73], we reasoned that local contact domains might differ in *Arabidopsis* depending on tissue identity. We employed Hi-C and RNA-seq datasets obtained independently from shoot and root tissues and extracted 'tissue-specific single-gene domains' [38, 39, 54]. While the majority of single-gene domains were common between shoot and root tissues, ~25% of single-gene domains from each tissue were defined as tissue-specific single-gene domains (Figure 5A). Pile-up analysis of these gene domains revealed stronger contact strengths in their respective tissues (Figure 5B).

Genes with single-gene domains in both tissues exhibited constitutive expression patterns, with little differential expression (Figure 5C, D) as well as relatively low Tau scores, a measure of tissue-specific expression (Figure 5E), suggesting that constitutively organized single-gene domains are associated with stable gene expression. Unexpectedly, however, despite the association of single-gene domains with transcriptional activity, there was little correlation between tissue-specific gene domain formation and tissue-specific gene expression (Figure 5D; Supplementary Figure S8). Given that gene domain formation is associated with

transcriptional activity, our results imply that the Hi-GDT analysis for two independent, different Hi-C datasets should be more optimized.

To better define the linkage of single-gene domain structural dynamics with gene expression changes, we put our focus on the gene domain structure changes of differentially expressed genes (DEGs) between shoot and root tissues and found that differences in gene expression correlated positively with intragenic contact frequency changes, but also negatively with contact frequency changes within surrounding chromatin regions outside gene bodies, consistent with previous findings (Supplementary Figure S9) [12, 33, 34]. We therefore developed Hi-GDT_{diff} to compare the gene domain insulation strength, which is represented by chromatin contact frequencies within intragenic regions over those within the 2-kb surrounding regions of individual genes, across different Hi-C datasets. Hi-GDT_{diff} identified differentially insulated single-gene domains in one tissue compared to the other tissue (Figure 6A; Supplementary Table S2). As expected, single-gene domains with enhanced insulation strength, which are marked by increased intragenic contact frequencies and decreased surrounding contact frequencies, exhibited higher transcription activity in the corresponding tissue (Figure 6B-E). To further validate our findings, we performed Gene Ontology (GO) analysis on differentially insulated single-gene domains between shoot and root tissues. Shoot-specific GO terms, including ‘Carbohydrate biosynthetic process’ and ‘Photosynthesis, light harvesting in photosystem I’, were significantly enriched for single-gene domains having enhanced insulation strength in shoots (Figure 6F; Supplementary Table S3), whereas GO terms related to transport systems, including ‘Phloem unloading’ and ‘Vascular transport’, were enriched for those with enhanced insulation strength in roots (Figure 6G; Supplementary Table S3). These results support the relevance of our approach for searching for differentially insulated single-gene domains that are associated with changes in gene expression.

We further applied our tool to other Hi-C datasets generated using *Arabidopsis* shoot tissues exposed to different environmental conditions or using different genotypes. Hi-GDT$_{diff}$ identified only a small number of single-gene domains (n = 11) with differential insulation strength between mock and heat shock conditions even with weaker Q-value cutoff (Q-value < 0.1), leading to mild changes in gene expression (Supplementary Figure S10A, B; Supplementary Table S2) (see **Discussion**). In contrast, when we applied Hi-GDT$_{diff}$ to different genotypes, it identified a substantial number of differentially insulated single-gene domains between wild type (Col-0) and *bmi1a bmi1b bmi1c* (*bmi1abc*), which also exhibited differential gene expression, supporting the reliability and broad applicability of our tool (Supplementary Figure S10C-F; Supplementary Table S2). Overall, these results suggest that Hi-GDT$_{diff}$ can be used to compare single-gene domain structures across different, independent Hi-C datasets and extract differentially insulated single-gene domains associated with changes in transcriptional activity. They also suggest that gene domain dynamics are more closely associated with tissue identity than with plant responses to environmental stimuli.

Single-gene domains are dynamically regulated during cellular reprogramming in *Arabidopsis*

Considering the close association between local contact domains and tissue identity, we hypothesized that gene domains are dynamically regulated during the cellular reprogramming process, similar to TADs in animal systems [69, 71, 74-76]. To test this hypothesis, we generated high-resolution Hi-C data with four biological replicates from *Arabidopsis* leaf explant–derived calli, which undergo genome-wide reprogramming of cellular identity (Supplementary Table S4). Based on the fact that the four replicates of callus Hi-C data showed

high Spearman's correlation coefficient with each other, we merged them into single callus Hi-C data that exhibited a distance-decay model similar to that of shoot and root Hi-C data (Supplementary Figure S11A, B). We also performed callus RNA-seq in parallel and confirmed that the data were also highly correlated each other (Supplementary Figure S11C). The high-resolution Hi-C contact maps of callus tissue showed numerous local contact domains, most with boundaries near gene borders (Figure 7A), as observed in other *Arabidopsis* tissues (Figure 4C), confirming that gene domains are prevalent 3D chromatin conformation units in *Arabidopsis*.

We then extracted genes showing dynamic changes in contact domain structures between shoots and calli. Although global Hi-C contact maps of calli showed a high degree of similarity to those of shoots, as evidenced by a Spearman's correlation coefficient of 0.88 at 25-kb resolution, we identified 961 differentially insulated single-gene domains including 697 genes with enhanced insulation in calli (Figure 7B; Supplementary Table S2), which were expressed at higher levels in calli compared to leaf explants (Figure 7C, D). Moreover, GO terms related to callus development, such as 'auxin biosynthetic process', and 'regulation of cell growth', were significantly enriched for these single-gene domains (Figure 7E; Supplementary Table S3). These results indicate that gene domain structures are tightly reorganized during the reprogramming of tissue identity, which may play a key role in this process.

Multigene domains are dynamically regulated by tissue type

We then analyzed dynamic changes in multigene domain structures in different tissues. We extracted tissue-specific multigene domains consisting of up to 10 genes: 2,109 shoot-specific, 4,305 root-specific, and 4,311 common multigene domains (Figure 8A). Since diverse types of

structural changes can occur in multigene domains, we focused on a specific case: a tissue-specific change in multigene domain structure from a dual-gene domain to a triple-gene domain with one side of the multigene domain border fixed. For instance, the shoot-specific dual-gene domain consisting of AT5G46260 and AT5G46270 changed to the triple-gene domains incorporating their 3′-downstream gene AT5G46280 in roots (Figure 8B). Pile-up images demonstrated the expansion of multigene domain structures and tissue-dependent structural dynamics (Figure 8C; Supplementary Figure S12A).

This approach allowed us to identify genes with multigene domain dynamics in association with gene expression levels by simply comparing the Hi-GDT$_{multi}$ results from different Hi-C datasets. Genes located at the borders of multigene domains are transcriptionally active, whereas genes within multigene domains are usually silenced (Figure 4G, H) [34]. Thus, structural changes from a dual-gene domain to a triple-gene domain may induce changes in the expression of genes comprising the domain: Border genes of dual-gene domains that become internalized in triple-gene domains during the structural transition are expected to be silenced. It should also be noted that since a TSS has higher chromatin accessibility than a TES [77, 78], changes in gene expression were more obvious for genes whose TSSs, located at domain borders, were moved to the internal region during structural changes (Supplementary Figure S13A-C).

Genes of shoot-specific dual-gene domains with their TSSs at domain borders that were internalized in roots (domain-internalized genes in roots) exhibited high expression in shoots but low expression in roots (Figure 8D, E; Supplementary Table S5). We also identified domain-internalized genes in shoots, meaning those that formed dual-gene domains in roots but were internalized into triple-gene domains in shoots. These genes strongly overlapped with DEGs that were more highly expressed in roots than in shoots (Figure 8F, G; Supplementary Table

S5).

Finally, we identified dynamic multigene domain structures by comparing dual- and triple-gene domains between callus and shoot tissues. In particular, we focused on genes in the middle of triple-gene domains in shoots that moved to the borders of dual-gene domains in calli (domain-insulated genes in calli) (Supplementary Figure S12B, C). These genes, including *CINNAMOYL COA REDUCTASE* (*CCR2*) and *NITRATE TRANSPORTER* (*NRT1.8*), were indeed transcriptionally activated in callus tissues (Figure 8H, I; Supplementary Table S5). Overall, our results demonstrate that Hi-GDT is an effective tool for identifying local chromatin structures, including single-gene and multigene domains. Furthermore, this tool can also be used to identify gene domains with dynamic structural changes in association with transcriptional activity.

Application of Hi-GDT in other plant species

Given that gene domains have been identified in other plant species, including tomato, maize and *Marchantia* (34), we examined the applicability of Hi-GDT to various plant species. As expected, both Hi-GDT$_{single}$ and Hi-GDT$_{multi}$ successfully identified single-gene and multigene domains characterized by strong internal contact frequencies (Figure 9A, B; Supplementary Figure S14). Furthermore, consistent with the findings in *Arabidopsis*, genes with single-gene domain structures or genes located at the borders of multigene domains were transcriptionally active, whereas genes located in the middle of multigene domains were transcriptionally repressed (Figure 9C, D). However, given a weak association between changes in insulation strength and gene expression levels in single-gene domains from tomato and *Marchantia*, Hi-GDT$_{diff}$ is most effective in high-depth *Arabidopsis* Hi-C analysis (Supplementary Figure S15).

Taking advantage of the broad applicability of Hi-GDT, we examined whether the gene

domain structure is evolutionarily conserved across plant species. To this end, we identified colinear blocks between *Arabidopsis* and tomato genomes, and between *Arabidopsis* and maize genomes. Notably, tomato and maize genes, which are homologous to *Arabidopsis* single-gene and dual-gene domains in colinear blocks, exhibited single- or dual-gene domain structures (Figure 9E, F; Supplementary Figure S16). Overall, these results indicate that gene domain structures and their formation mechanisms are widely conserved across various plant species.

Discussion

Numerous bioinformatics tools, including those using deep learning models, have been developed to identify hierarchical chromatin structures in animal genomes [43-47, 79, 80]. Although some of them have been applied to analyze TAD-like structures and compartment domains in large genome plants [28, 43-46], they usually showed low reliability and reproducibility in identifying fine-scale chromatin contact domains (Supplementary Figure S4A). This limitation is mainly due to the combined effects of weak interaction strength within contact domains and high noise of the Hi-C matrix at a high resolution. To overcome this limitation, we developed a novel tool designed to analyze fine-scale local chromatin contact domains, with a particular focus on single-gene and multigene domains that are established at gene boundaries (Supplementary Figure S3) [34]. This semi-targeted approach allows for high sensitivity and reproducibility, outperforming existing tools (Figure 2; Supplementary Figure S4B). For example, although OnTAD, which is optimized to identify hierarchical (nested) contact domain structures [47], identified a large number of potential fine-scale contact domains (Figure 2A, B), its low reproducibility may limit the broad application (Figure 2; Supplementary Figure S4B). Indeed, our tool successfully identified local contact domains in

various plant species, including *Arabidopsis*, tomato, soybean, rice, maize, *Jatropha curcas* and *Marchantia*, and found that gene domains are conserved across plant species. Hi-GDT showed higher reproducibility than conventional domain callers, enabling effective analysis at a high resolution. This advantage makes it feasible to analyze 3D gene domain dynamics in different tissues and environmental conditions in association with their transcriptional activity.

Our findings confirm that changes in local chromatin structure, especially gene domains, are associated with transcriptional changes. In general, local contact domains identified by Hi-GDT showed high chromatin accessibility at their boundaries (Figure 3C and 4E). Moreover, RNA polymerase II (RNA Pol II) is highly enriched at the gene domain boundaries (Supplementary Figure S17), and the binding of multiple transcription factors is crucial for the formation of local 3D chromatin structure [7, 34, 81]. Hence, we suggest that the concerted action of transcription factors and RNA Pol II at gene borders plays a crucial role in shaping local chromatin structure in *Arabidopsis*. Indeed, transcriptional activity and RNA Pol II binding influence the single-gene domain structures of both animals and plants, as revealed by the finding that inhibiting RNA Pol II activity alters local chromatin structure at individual genes [12, 33].

Despite the strong association between local contact domain formation and transcriptional activity, the biochemical relevance of local contact domain formation to the regulation of 3D-conformation-dependent gene expression remains unclear. Considering the extremely short intergenic sequences in the small genome of *Arabidopsis*, local contact domain formation might be particularly important in that proximal *cis*-regulatory elements near gene borders play an additional role in regulating adjacent genes. Indeed, deletion of the promoter region of a gene affects expression of its adjacent gene connected by a promoter-promoter loop [82]. Alternatively, given that these local contact domains frequently form at gene borders,

efficient transcription might be facilitated by the anchoring of border regions, similar to the transcription factory model that has been suggested for plants with large genomes [26, 28]. Integrative analysis of Hi-GDT data with intergenic genomic components, such as super enhancers, could provide a comprehensive understanding of the intricate principles governing the relationship between 3D chromatin structure and gene regulation.

Our understanding of the biological relevance of local contact domains in plants also remains limited. Using Hi-GDT, we determined that stable local contact domain structures are associated with constitutive gene expression. In addition, genes with the structural dynamics of local contact domains are frequently related to tissue identity. Notably, the structural plasticity of gene domains in response to environmental stimuli (such as heat) is relatively low (Supplementary Figure S10), suggesting that local chromatin structures are more tightly linked with cell or tissue identity compared to environmental conditions. In this context, the low structural dynamics of gene domains in response to environmental stimuli might also be due to the dilution effect of heterogeneous cell types. Hi-GDT should facilitate functional studies of local contact domains in *Arabidopsis* and provide broader insight into the biological impact of local chromatin organization in plants.

We placed a particular emphasis on the relevance of our tool for analyzing dynamic structural changes associated with transcriptional activity at the gene scale, as this issue has not previously been analyzed due to the lack of optimal analytical tools. Although the analysis of local chromatin domains requires high sequencing depth for precise analysis, this limitation can be overcome using micrococcal nuclease–based techniques such as highly sensitive transposase-mediated analysis of chromatin (Hi-TrAC), Micro-C or chemical-crosslinking assisted proximity capture (CAP-C) [81-84]. Notably, the newer cutting-edge techniques known as CAP-C and Micro-C-XL can be used to generate contact maps at an extremely high

resolution of 200 bp to support analysis of fine-scale chromatin structure in plants [33, 82]. Hi-GDT is compatible with high-resolution contact maps, and the combinations of these techniques with Hi-GDT should be a powerful tool for analyzing fine-scale local contact domains.

Availability of source code and requirements

- Project name: Hi-GDT
- Project homepage: https://github.com/CDL-HongwooLee/Hi-GDT
- Operating system(s): Linux
- Programming language: Python
- Other requirements: python 3.8, subprocess, numpy, pickle, multiprocessing, PIL, functools, itertools, scipy
- License: MIT license
- RRID: SCR_025798
- Software Heritage PID: swh:1:snp:a4d7864a7adfdf451f1058edde2c0014d7b51fe6

Data availability

All publicly available datasets used in this study are listed in Supplementary Table S1. The source codes and step-by-step instructions of Hi-GDT are available from the GitHub repository [85]. Snapshots of the code and data are available in Software Heritage [86]. Raw data and all implemented codes used in this study have been deposited at Zenodo [52]. Hi-C and RNA-seq data produced in this study were deposited in NCBI under accession number PRJNA1112149.

List of abbreviations

3D: three-dimensional; TAD: topologically associating domain; TSS: transcriptional start site; TES: transcriptional end site; OE: observed contact frequency/expected contact frequency; DI: directionality index; GO: gene ontology; DEG: differentially expressed gene; TPM: transcripts per million.

Funding

This work was supported by the Basic Science Research (NRF-2022R1A2B5B02001266) and Basic Research Laboratory (NRF-2022R1A4A3024451) programs provided by the National Research Foundation of Korea.

Conflict of interest

The authors declare no conflict of interest.

Acknowledgments

Author contributions: P.J.S. and H.L. conceived and designed the study. H.L. performed all analyses and experiments. P.J.S. and H.L. wrote the manuscript.

References

1. Dixon JR, Jung I, Selvaraj S, et al. Chromatin architecture reorganization during stem cell differentiation. *Nature*. 2015;518:331-6. doi:10.1038/nature14222.
2. Fukaya T, Lim B, Levine M. Enhancer Control of Transcriptional Bursting. *Cell*. 2016;166:358-68. https://doi.org/10.1016/j.cell.2016.05.025.
3. Gonzalez-Sandoval A, Gasser SM. On TADs and LADs: Spatial Control Over Gene Expression. *Trends Genet*. 2016;32:485-95. doi:10.1016/j.tig.2016.05.004.
4. Gu B, Swigut T, Spencley A, et al. Transcription-coupled changes in nuclear mobility

of mammalian cis-regulatory elements. *Science*. 2018;359:1050-5. doi:10.1126/science.aao3136.

5. Kaushal A, Mohana G, Dorier J, et al. CTCF loss has limited effects on global genome architecture in Drosophila despite critical regulatory functions. *Nat Commun*. 2021;12:1011. doi:10.1038/s41467-021-21366-2.
6. Schoenfelder S, Fraser P. Long-range enhancer–promoter contacts in gene expression control. Nat Rev Genet. 2019;20:437-55. doi:10.1038/s41576-019-0128-0.
7. Stadhouders R, Vidal E, Serra F, et al. Transcription factors orchestrate dynamic interplay between genome topology and gene regulation during cell reprogramming. *Nat Genet*. 2018;50:238-49. doi:10.1038/s41588-017-0030-7.
8. Dixon JR, Selvaraj S, Yue F, et al. Topological domains in mammalian genomes identified by analysis of chromatin interactions. *Nature*. 2012;485:376-80. doi:10.1038/nature11082.
9. Nora EP, Lajoie BR, Schulz EG, et al. Spatial partitioning of the regulatory landscape of the X-inactivation centre. *Nature*. 2012;485:381-5. doi:10.1038/nature11049.
10. Rao SSP, Huntley MH, Durand NC, et al. A 3D Map of the Human Genome at Kilobase Resolution Reveals Principles of Chromatin Looping. *Cell*. 2014;159:1665-80. https://doi.org/10.1016/j.cell.2014.11.021.
11. Rao SSP, Huang S-C, Glenn SHB, et al. Cohesin Loss Eliminates All Loop Domains. *Cell*. 2017;171:305-20.e24. https://doi.org/10.1016/j.cell.2017.09.026.
12. Rowley MJ, Nichols MH, Lyu X, et al. Evolutionarily Conserved Principles Predict 3D Chromatin Organization. *Mol Cell*. 2017;67:837-52.e7. https://doi.org/10.1016/j.molcel.2017.07.022.
13. Schwarzer W, Abdennur N, Goloborodko A, et al. Two independent modes of chromatin organization revealed by cohesin removal. *Nature*. 2017;551:51-6. doi:10.1038/nature24281.
14. Rowley MJ, Corces VG. Organizational principles of 3D genome architecture. *Nat Re v Genet*. 2018;19:789-800. doi:10.1038/s41576-018-0060-8.
15. Beagan JA, Phillips-Cremins JE. On the existence and functionality of topologically associating domains. *Nat Genet*. 2020;52:8-16. doi:10.1038/s41588-019-0561-1.
16. Alipour E, Marko JF. Self-organization of domain structures by DNA-loop-extruding

enzymes. *Nucleic Acids Res*. 2012;40:11202-12. doi:10.1093/nar/gks925.

17. Sanborn AL, Rao SSP, Huang S-C, et al. Chromatin extrusion explains key features of loop and domain formation in wild-type and engineered genomes. *Proc Natl Acd Sci U S A*. 2015;112:E6456-65. doi:10.1073/pnas.1518552112.

18. Fudenberg G, Imakaev M, Lu C, et al. Formation of Chromosomal Domains by Loop Extrusion. *Cell Rep*. 2016;15:2038-49. https://doi.org/10.1016/j.celrep.2016.04.085.

19. Nichols MH, Corces VG. Principles of 3D compartmentalization of the human genome. Cell Rep. 2021;35:109330. https://doi.org/10.1016/j.celrep.2021.109330.

20. Heger P, Marin B, Bartkuhn M, et al. The chromatin insulator CTCF and the emergence of metazoan diversity. *Proc Natl Acd Sci U S A*. 2012;109:17507-12. doi:10.1073/pnas.1111941109.

21. Dong P, Tu X, Chu P-Y, et al. 3D Chromatin Architecture of Large Plant Genomes Determined by Local A/B Compartments. *Mol Plant*. 2017;10:1497-509. https://doi.org/10.1016/j.molp.2017.11.005.

22. Dong Q, Li N, Li X, et al. Genome-wide Hi-C analysis reveals extensive hierarchical chromatin interactions in rice. *Plant J*. 2018;94:1141-56. https://doi.org/10.1111/tpj.13925.

23. Dong P, Tu X, Li H, et al. Tissue-specific Hi-C analyses of rice, foxtail millet and maize suggest non-canonical function of plant chromatin domains. *J Integr Plant Biol*. 2020;62:201-17. https://doi.org/10.1111/jipb.12809.

24. Huang Y, An J, Sircar S, et al. HSFA1a modulates plant heat stress responses and alters the 3D chromatin organization of enhancer-promoter interactions. *Nat Commun*. 2023;14 1:469. doi:10.1038/s41467-023-36227-3.

25. Wang M, Wang P, Lin M, et al. Evolutionary dynamics of 3D genome architecture following polyploidization in cotton. *Nat Plants*. 2018;4:90-7. doi:10.1038/s41477-017-0096-3.

26. Concia L, Veluchamy A, Ramirez-Prado JS, et al. Wheat chromatin architecture is organized in genome territories and transcription factories. *Genome Biol*. 2020;21:104. doi:10.1186/s13059-020-01998-1.

27. Sun Y, Dong L, Zhang Y, et al. 3D genome architecture coordinates trans and cis regulation of differentially expressed ear and tassel genes in maize. *Genome Biol*. 2020;21:143. doi:10.1186/s13059-020-02063-7.

28. Liao Y, Wang J, Zhu Z, et al. The 3D architecture of the pepper genome and its relationship to function and evolution. *Nat Commun*. 2022;13:3479. doi:10.1038/s41467-022-31112-x.
29. Pei L, Huang X, Liu Z, et al. Dynamic 3D genome architecture of cotton fiber reveals subgenome-coordinated chromatin topology for 4-staged single-cell differentiation. *Genome Biol*. 2022;23:45. doi:10.1186/s13059-022-02616-y.
30. Huang Y, Sicar S, Ramirez-Prado JS, et al. Polycomb-dependent differential chromatin compartmentalization determines gene coregulation in Arabidopsis. *Genome Res*. 2021;31:1230-44. doi:10.1101/gr.273771.120.
31. Deng L, Zhou Q, Zhou J, et al. 3D organization of regulatory elements for transcriptional regulation in Arabidopsis. *Genome Biol*. 2023;24:181. doi:10.1186/s13059-023-03018-4.
32. Yin X, Romero-Campero FJ, Yang M, et al. Binding by the Polycomb complex component BMI1 and H2A monoubiquitination shape local and long-range interactions in the Arabidopsis genome. *Plant Cell*. 2023;35:2484-503. doi:10.1093/plcell/koad112.
33. Sun L, Zhou J, Xu X, et al. Mapping nucleosome-resolution chromatin organization and enhancer-promoter loops in plants using Micro-C-XL. *Nat Commun*. 2024;15:35. doi:10.1038/s41467-023-44347-z.
34. Lee H, Seo Pil J. Accessible gene borders establish a core structural unit for chromatin architecture in Arabidopsis. *Nucleic Acids Res*. 2023;51:10261-77. doi:10.1093/nar/gkad710.
35. Nützmann H-W, Doerr D, Ramírez-Colmenero A, et al. Active and repressed biosynthetic gene clusters have spatially distinct chromosome states. *Proc Natl Acd Sci U S A*. 2020;117:13800-9. doi:10.1073/pnas.1920474117.
36. Koo D, Lee HG, Bae SH, et al. Callus proliferation-induced hypoxic microenvironment decreases shoot regeneration competence in Arabidopsis. *Mol Plant*. 2024;17:395-408. https://doi.org/10.1016/j.molp.2024.01.009.
37. Sayers EW, Bolton EE, Brister JR, et al. Database resources of the national center for biotechnology information. *Nucleic Acids Res*. 2022;50:D20-6. https://doi.org/10.1093/nar/gkab1112
38. Sun L, Jing Y, Liu X, et al. Heat stress-induced transposon activation correlates with 3D chromatin organization rearrangement in Arabidopsis. *Nat Commun*. 2020;11:1886.

doi:10.1038/s41467-020-15809-5.
39. Sun L, Cao Y, Li Z, et al. Conserved H3K27me3-associated chromatin looping mediates physical interactions of gene clusters in plants. *J Integr Plant Biol*. 2023;65:1966-82. https://doi.org/10.1111/jipb.13502.
40. Wang C, Liu C, Roqueiro D, et al. Genome-wide analysis of local chromatin packing in Arabidopsis thaliana. *Genome Res*. 2015;25:246-56. doi:10.1101/gr.170332.113
41. Liu C, Wang C, Wang G, et al. Genome-wide analysis of chromatin packing in Arabidopsis thaliana at single-gene resolution. *Genome Res*. 2016;26:1057-68. doi:10.1101/gr.204032.116.
42. Karaaslan ES, Wang N, Faiß N, et al. Marchantia TCP transcription factor activity correlates with three-dimensional chromatin structure. *Nat Plants*. 2020;6:1250-61. doi:10.1038/s41477-020-00766-0.
43. Durand NC, Shamim MS, Machol I, et al. Juicer Provides a One-Click System for Analyzing Loop-Resolution Hi-C Experiments. *Cell Syst*. 2016;3:95-8. doi:10.1016/j.cels.2016.07.002.
44. Ramírez F, Bhardwaj V, Arrigoni L, et al. High-resolution TADs reveal DNA sequences underlying genome organization in flies. *Nat Commun*. 2018;9:189. doi:10.1038/s41467-017-02525-w.
45. Wolff J, Bhardwaj V, Nothjunge S, et al. Galaxy HiCExplorer: a web server for reproducible Hi-C data analysis, quality control and visualization. *Nucleic Acids Res*. 2018;46:W11-6. doi:10.1093/nar/gky504.
46. Wolff J, Rabbani L, Gilsbach R, et al. Galaxy HiCExplorer 3: a web server for reproducible Hi-C, capture Hi-C and single-cell Hi-C data analysis, quality control and visualization. *Nucleic Acids Res*. 2020;48:W177-84. doi:10.1093/nar/gkaa220.
47. An L, Yang T, Yang J, et al. OnTAD: hierarchical domain structure reveals the divergence of activity among TADs and boundaries. *Genome Biol*. 2019;20:282. doi:10.1186/s13059-019-1893-y.
48. Harris CR, Millman KJ, van der Walt SJ, et al. Array programming with NumPy. *Nature*. 2020;585:357-62. doi:10.1038/s41586-020-2649-2.
49. Ramírez F, Ryan DP, Grüning B, et al. deepTools2: a next generation web server for deep-sequencing data analysis. *Nucleic Acids Res*. 2016;44:W160-5.

doi:10.1093/nar/gkw257.
50. Lu Z, Hofmeister BT, Vollmers C, et al. Combining ATAC-seq with nuclei sorting for discovery of cis-regulatory regions in plant genomes. *Nucleic Acids Res*. 2017;45:e41. doi:10.1093/nar/gkw1179.
51. Liu Y, Tian T, Zhang K, et al. PCSD: a plant chromatin state database. *Nucleic Acids Res*. 2018;46:D1157-67. doi:10.1093/nar/gkx919.
52. Lee H and Seo PJ. Hi-GDT: A Hi-C-based 3D gene domain analysis tool for analyzing local chromatin contacts in plants. *Zenodo*. 2024. doi:10.5281/zenodo.11201542
53. Jamge B, Lorković ZJ, Axelsson E, et al. Histone variants shape chromatin states in Arabidopsis. *eLife*. 2023;12:RP87714. doi:10.7554/eLife.87714.
54. Potter KC, Wang J, Schaller GE, et al. Cytokinin modulates context-dependent chromatin accessibility through the type-B response regulators. *Nat Plants*. 2018;4:1102-11. doi:10.1038/s41477-018-0290-y.
55. Dobin A, Davis CA, Schlesinger F, et al. STAR: ultrafast universal RNA-seq aligner. *Bioinformatics*. 2013;29:15-21. doi:10.1093/bioinformatics/bts635.
56. Li B, Dewey CN. RSEM: accurate transcript quantification from RNA-Seq data with or without a reference genome. *BMC Bioinform*. 2011;12:323. doi:10.1186/1471-2105-12-323.
57. Love MI, Huber W, Anders S. Moderated estimation of fold change and dispersion for RNA-seq data with DESeq2. *Genome Biol*. 2014;15:550. doi:10.1186/s13059-014-0550-8.
58. Yanai I, Benjamin H, Shmoish M, et al. Genome-wide midrange transcription profiles reveal expression level relationships in human tissue specification. *Bioinformatics*. 2005;21:650-9. doi:10.1093/bioinformatics/bti042.
59. Martín G, Márquez Y, Mantica F, et al. Alternative splicing landscapes in Arabidopsis thaliana across tissues and stress conditions highlight major functional differences with animals. *Genome Biol*. 2021;22:35. doi:10.1186/s13059-020-02258-y.
60. Ashburner M, Ball CA, Blake JA, et al. Gene Ontology: tool for the unification of biology. *Nat Genet*. 2000;25:25-9. doi:10.1038/75556.
61. Carbon S, Ireland A, Mungall CJ, et al. AmiGO: online access to ontology and annotation data. *Bioinformatics*. 2009;25:288-9. doi:10.1093/bioinformatics/btn615.
62. The Gene Ontology C, Aleksander SA, Balhoff J, et al. The Gene Ontology

knowledgebase in 2023. *Genetics*. 2023;224:iyad031. doi:10.1093/genetics/iyad031.

63. Wang Y, Tang H, Wang X, et al. Detection of colinear blocks and synteny and evolutionary analyses based on utilization of MCScanX. *Nat Prot*. 2024;19:2206-29. doi:10.1038/s41596-024-00968-2.

64. Altschul SF, Madden TL, Schäffer AA, et al. Gapped BLAST and PSI-BLAST: a new generation of protein database search programs. *Nucleic Acids Res*. 1997;25 17:3389-402. doi:10.1093/nar/25.17.3389.

65. Wang Y, Tang H, DeBarry JD, et al. MCScanX: a toolkit for detection and evolutionary analysis of gene synteny and collinearity. *Nucleic Acids Res*. 2012;40:e49. doi:10.1093/nar/gkr1293.

66. Hunter JD. Matplotlib: A 2D Graphics Environment. *Comput Sci Eng*. 2007;9:90-5. doi:10.1109/MCSE.2007.55.

67. Waskom ML. Seaborn: statistical data visualization. *J Open Source Softw*. 2021;6 60:3021. https://doi.org/10.21105/joss.03021.

68. Harris HL, Gu H, Olshansky M, et al. Chromatin alternates between A and B compartments at kilobase scale for subgenic organization. *Nat Commun*. 2023;14:3303. doi:10.1038/s41467-023-38429-1.

69. Bonev B, Mendelson CN, Szabo Q, et al. Multiscale 3D Genome Rewiring during Mouse Neural Development. *Cell*. 2017;171:557-72.e24. https://doi.org/10.1016/j.cell.2017.09.043.

70. Ke Y, Xu Y, Chen X, et al. 3D Chromatin Structures of Mature Gametes and Structural Reprogramming during Mammalian Embryogenesis. *Cell*. 2017;170:367-81.e20. https://doi.org/10.1016/j.cell.2017.06.029.

71. Winick-Ng W, Kukalev A, Harabula I, et al. Cell-type specialization is encoded by specific chromatin topologies. *Nature*. 2021;599:684-91. doi:10.1038/s41586-021-04081-2.

72. Rahman S, Dong P, Apontes P, et al. Lineage specific 3D genome structure in the adult human brain and neurodevelopmental changes in the chromatin interactome. *Nucleic Acids Res*. 2023;51:11142-61. doi:10.1093/nar/gkad798.

73. Rajderkar S, Barozzi I, Zhu Y, et al. Topologically associating domain boundaries are required for normal genome function. *Commun Biol*. 2023;6:435. doi:10.1038/s42003-023-04819-w.

74. Fraser J, Ferrai C, Chiariello AM, et al. Hierarchical folding and reorganization of chromosomes are linked to transcriptional changes in cellular differentiation. *Mol Syst Biol*. 2015;11:852. https://doi.org/10.15252/msb.20156492.
75. Zhang Y, Li T, Preissl S, et al. Transcriptionally active HERV-H retrotransposons demarcate topologically associating domains in human pluripotent stem cells. *Nat Genet*. 2019;51:1380-8. doi:10.1038/s41588-019-0479-7.
76. Wang J, Yu H, Ma Q, et al. Phase separation of OCT4 controls TAD reorganization to promote cell fate transitions. *Cell Stem Cell*. 2021;28:1868-83.e11. https://doi.org/10.1016/j.stem.2021.04.023.
77. Starks RR, Biswas A, Jain A, et al. Combined analysis of dissimilar promoter accessibility and gene expression profiles identifies tissue-specific genes and actively repressed networks. *Epigenet Chromatin*. 2019;12:16. doi:10.1186/s13072-019-0260-2.
78. Sanghi A, Gruber JJ, Metwally A, et al. Chromatin accessibility associates with protein -RNA correlation in human cancer. *Nat Commun*. 2021;12:5732. doi:10.1038/s41467-021-25872-1.
79. Zhang P, Wu Y, Zhou H, et al. CLNN-loop: a deep learning model to predict CTCF-mediated chromatin loops in the different cell lines and CTCF-binding sites (CBS) pair types. *Bioinformatics*. 2022;38:4497-504. doi: 10.1093/bioinformatics/btac575.
80. Zhang P, Wu H. An attention-based deeplearning model for identifying chromatin interactions. *IEEE J Biomed Health Inform*. 2023;27:4559-68. doi: 10.1109/JBHI.2023.3292299.
81. Liu S, Cao Y, Cui K, et al. Hi-TrAC reveals division of labor of transcription factors in organizing chromatin loops. *Nat Commun*. 2022;13:6679. doi:10.1038/s41467-022-34276-8.
82. Zhang Y, Dong Q, Wang Z, et al. A fine-scale Arabidopsis chromatin landscape reveals chromatin conformation-associated transcriptional dynamics. *Nat Commun*. 2024;15:3253. doi:10.1038/s41467-024-47678-7.
83. Hsieh T-HS, Weiner A, Lajoie B, et al. Mapping Nucleosome Resolution Chromosome Folding in Yeast by Micro-C. *Cell*. 2015;162:108-19. https://doi.org/10.1016/j.cell.2015.05.048.
84. Hsieh T-HS, Fudenberg G, Goloborodko A et al. Micro-C XL: assaying chromosome conformation from the nucleosome to the entire genome.

Nat Methods. 2016;13:1009-11. doi:10.1038/nmeth.4025.

85. Hayward RJ, Ebbecke T, Fricke H et al.. Hi-GDT : A Hi-C-based 3D gene do main analysis tool for analyzing local chromatin contacts in plants. [Computer s oftware]. Software Heritage 2025. https://archive.softwareheritage.org/browse/snap shot/a4d7864a7adfdf451f1058edde2c0014d7b51fe6/directory/.

86. Lee H. Hi-GDT [Computer software]. *GitHub*. 2024. https://github.com/CDL-Ho ngwooLee/Hi-GDT. Accessed 19 May 2024.

Figure legends

Figure 1. Workflow and strategy of Hi-GDT for identifying local contact domains.

(**A**) The Hi-GDT workflow. Hi-GDT analysis begins with a .hic file generated by Juicer software. Hi-GDT identifies single- and multigene domains (Hi-GDT$_{single}$ and Hi-GDT$_{multi}$, respectively). Hi-GDT also provides an analysis of the structural dynamics of single-gene domains, which are associated with changes in gene expression (Hi-GDT$_{diff}$). (**B** and **C**) Schematic diagrams illustrating the target and control regions in a Hi-C contact map, which are defined for gene domain identification by Hi-GDT. The regions used for single-gene domain identification (**B**) and multigene domain identification (**C**) are shown. Target regions are shown in red, and control regions are shown in green or yellow. The line at the bottom indicates the diagonal axis in a Hi-C contact map, and the black triangle indicates an individual gene. In (**C**), target and control regions used to identify quadruple-gene domains are shown as an example. Boundaries of a multigene domain are indicated by blue lines.

Figure 2. Benchmarking of Hi-GDT against conventional domain callers.

(**A**) Comparison of the number of contact domains identified by Arrowhead, HiCExplorer, OnTAD, and Hi-GDT. Local contact domains were identified at 250-, 500-, and 1,000-bp

resolution. (**B**) Density plots showing the size distribution of contact domains identified by each domain caller at 250- and 500-bp resolution. (**C**) Pile-up images of Hi-C contact matrices for contact domains identified by each domain caller. The images of contact domains identified at 250- (top) and 500-bp (bottom) resolution are shown. Black lines indicate the boundaries of the identified contact domains. (**D**) Comparison of the Jaccard index values of contact domains identified by each domain caller across different datasets. Domain calling was conducted with two technical replicates subsampled (80%) from a merged Hi-C dataset, two biological replicates, and two distinct datasets from different batches at 250-, 500-, and 1,000-bp resolution. Jaccard index was calculated as the intersection over the union between two sets of identified contact domains.

Figure 3. Validation of single-gene domains identified by Hi-GDT$_{single}$.

(**A**) Pile-up images of Hi-C matrices for single-gene domains identified by Hi-GDT$_{single}$. Pile-up images of genes with single-gene domains (Hi-GDT$_{single}$) and without single-gene domains (other genes) at 250-bp resolution are shown. Black lines indicate gene borders. (**B**-**D**) Metagene plots of DI score (**B**), chromatin accessibility (**C**), and compartment eigenvector (**D**) for Hi-GDT$_{single}$-identified (Hi-GDT$_{single}$) or remaining genes (other genes). (**E**) The proportion of local A/B compartments within genic regions with or without single-gene domains. The local A/B compartments at TSS, gene body, and TES regions were analyzed. (**F**) Expression levels of genes with or without single-gene domains. Log$_2$(transcripts per million [TPM] + 1) values were used to quantify gene expression levels. The *P*-value was calculated by a two-sided Mann–Whitney U-test. (**G**) Distribution of chromatin states at TSS, gene body, and TES regions for genes with or without single-gene domains. Twenty-six chromatin states were used for analysis, including 6 constitutive heterochromatin (H1–H6), 6 facultative heterochromatin (F1–F6), 3

accessible intergenic (I1–I3), and 11 euchromatin (E1–E11) states.

Figure 4. Validation of multigene domains identified by Hi-GDT$_{multi}$.

(**A**) The number of multigene domains identified by Hi-GDT$_{multi}$ based on their number of constituent genes. (**B**) The size distribution of identified multigene domains. (**C**) An example region in a Hi-C contact map showing multigene domains identified by Hi-GDT$_{multi}$. The blue lines indicate the boundaries of multigene domains, and black boxes indicate gene borders. (**D**) Pile-up images of Hi-C matrices for identified multigene domains based on the number of constituent genes. Black lines indicate the boundaries of identified multigene domains. (**E** and **F**) Metaplots of chromatin accessibility (**E**) and compartment eigenvector (**F**) for the identified multigene domains. (**G**) The distribution of chromatin states of genes located at boundaries of multigene domains (border), internal regions of multigene domains (inner), and outside of multigene domains (other genes). Twenty-six chromatin states were used for analysis, including 6 constitutive heterochromatin (H1–H6), 6 facultative heterochromatin (F1–F6), 3 accessible intergenic (I1–I3), and 11 euchromatin (E1–E11) states. (**H**) Expression levels of genes located at boundaries of multigene domains, internal regions of multigene domains, and outside of multigene domains. Log_2(transcripts per million [TPM] + 1) values were used to quantify gene expression levels. *P*-values were calculated by a Kruskal–Wallis with Dunn's *post-hoc* test. In (**G**) and (**H**), genes forming single-gene domains were excluded. (**I**) The proportion of gene pairs with similar chromatin states in quadruple-gene domains. Four nearby genes lacking quadruple-gene domains were used for the random control (control genes). Gene 1 (g1) to gene 4 (g4) indicate the sequential order of genes from the 5′ to 3′ direction within a quadruple-gene domain. The ratio between gene pairs from quadruple-gene domains and control nearby genes is indicated above the graph.

Figure 5. Identification of single-gene domains in shoot and root tissues.

(**A**) Venn diagram illustrating the number of Hi-GDT$_{single}$-identified single-gene domains from shoot and root tissues. (**B**) Pile-up images of Hi-C matrices for single-gene domains identified from shoot and root tissues based on their tissue specificity. Images of shoot (top) and root (bottom) Hi-C contact maps are shown. Black lines indicate the boundaries of the identified single-gene domains; +, the presence of a single-gene domain; −, the absence of a single-gene domain. (**C** and **D**) Changes in gene expression levels for single-gene domains grouped by their tissue specificity. Absolute value $\log_2$(fold change) of gene expression levels (**C**) and the proportions of overlap with DEGs between shoot and root tissues (**D**) in each group are shown. (**E**) Tau scores of single-gene domains grouped by their tissue specificity. In (**C**) and (**E**), different letters indicate statistically significant differences determined by a Kruskal–Wallis with Dunn's *post-hoc* test (P-value < 0.05).

Figure 6. Identification of shoot- and root-specific active single-gene domains.

(**A**) Pile-up images showing differences between shoot and root Hi-C matrices for differentially insulated single-gene domains identified by Hi-GDT$_{diff}$. The differences in pile-up images of single-gene domains with enhanced insulation strength in shoots (upper) and roots (lower) are shown. Black lines indicate the boundaries of the identified single-gene domains. (**B** and **C**) Changes in gene expression levels for single-gene domains exhibiting enhanced insulation strength in shoots. $\log_2$(fold change) of expression levels (**B**) and the proportions of overlap with DEGs between shoot and root tissues (**C**) in each group are shown. (**D** and **E**) Changes in gene expression levels for single-gene domains exhibiting enhanced insulation strength in roots. $\log_2$(fold change) of expression levels (**D**) and the proportions of overlap with DEGs (**E**) in

each group are shown. In (**B**) and (**D**), *P*-values were calculated by two-sided Mann–Whitney U-tests. (**F** and **G**) Top 10 significantly enriched GO terms of single-gene domains exhibiting enhanced insulation strength in shoots (**F**) and roots (**G**). GO terms with *P*-value < 0.05 and number of genes in each category > 1 are shown.

Figure 7. Identification of callus-specific active single-gene domains.

(**A**) Hi-C contact map of callus tissue with local contact domains identified by Hi-GDT. Black squares indicate gene borders, and blue lines indicate the boundaries of the identified contact domains. (**B**) Pile-up images showing differences between shoot and callus Hi-C matrices for differentially insulated single-gene domains identified by Hi-GDT$_{diff}$. The differences in pile-up images of single-gene domains with enhanced insulation strength in shoot (left) and callus tissues (right) are shown. Black lines indicate the boundaries of the identified single-gene domains. (**C** and **D**) Changes in gene expression levels for single-gene domains exhibiting enhanced insulation strength in calli. Log$_2$(fold change) of expression levels (**C**) and the proportions of overlap with DEGs between leaf explant and callus tissues (**D**) in each group are shown. In (**C**), the *P*-value was calculated by a two-sided Mann–Whitney U-test. (**E**) Top 20 significantly enriched GO terms of single-gene domains exhibiting enhanced insulation strength in calli. GO terms with *P*-value < 0.05 and number of genes in each category > 1 are shown.

Figure 8. Identification of dynamically regulated multigene domains in different tissue types.

(**A**) Venn diagram illustrating the number of multigene domains identified from shoot and root Hi-C datasets at 500-bp resolution. (**B**) An example region showing the transition from a dual-

gene domain to a triple-gene domain depending on tissue type. Upper-right triangles represent Hi-C maps in shoots, while lower-left triangles represent Hi-C maps in roots. Black boxes indicate the gene borders, and green and navy blue lines indicate the boundaries of identified multigene domains in shoot and root tissues, respectively. (**C**) Pile-up images of Hi-C matrices for differential multigene domains between shoot and root tissues. Dual-gene domains specifically identified in shoots and expanded to triple-gene domains in roots were collected. Pile-up images of dual-gene domains expanded to the 5′ direction (top) and the 3′ direction (bottom) in the formation of triple-gene domains are shown. Black lines indicate the boundaries of dual-gene domains. (**D** and **E**) Changes in gene expression levels for domain-internalized genes in roots (internalized). Log_2(fold change) of expression levels (**D**) and the proportions of overlap with DEGs between shoot and root tissues (**E**) for domain-internalized genes from dual-gene domains compared to the control genes located in dual-gene domains without structural changes in both tissues (unchanged) are shown. (**F** and **G**) Changes in gene expression levels for domain-internalized genes in shoots (internalized). Log_2(fold change) of expression levels (**F**) and the proportions of overlap with DEGs between shoot and root tissues (**G**) for domain-internalized genes from dual-gene domains compared to the control genes located in dual-gene domains without structural changes in both tissues (unchanged) are shown. (**H** and **I**) Changes in gene expression levels for domain-insulated genes in callus tissue, which are located within triple-gene domains in shoots but insulated at a border of a dual-gene domain in callus (insulated). Log_2(fold change) of expression levels (**H**) and the proportions of overlap with DEGs between leaf explant and callus tissues (**I**) for domain-insulated genes from triple-gene domains compared to the control genes located in dual-gene domains without structural changes in both tissues (unchanged) are shown. In (**D**), (**F**), and (**H**), *P*-values were calculated by two-sided Mann–Whitney U-tests. In (**B**)-(**I**), genes that form dual-gene domains with their TSSs at

the domain border were included in the analysis.

Figure 9. Application of Hi-GDT on other plant species.

(**A**) Pile-up images of Hi-C matrices for single-gene domains identified by Hi-GDT$_{single}$ in tomato, maize and *Marchantia*. Pile-up images of genes with single-gene domains (Hi-GDT$_{single}$) and without single-gene domains (other genes) at 250-bp resolution are shown. Black lines indicate gene borders in each species. (**B**) Pile-up images of Hi-C matrices for identified multigene domains in tomato, maize and *Marchantia* based on the number of constituent genes. Black lines indicate the boundaries of identified multigene domains. (**C**) Expression levels of genes with or without single-gene domains. *P*-values were calculated by two-sided Mann–Whitney U-tests. (**D**) Expression levels of genes located at boundaries of multigene domains, internal regions of multigene domains, and outside of multigene domains. *P*-values were calculated by Kruskal–Wallis with Dunn's *post-hoc* tests. Genes forming single-gene domains were excluded from all categories. In (**C**) and (**D**), $\log_2$(transcripts per million [TPM] + 1) values were used to quantify gene expression levels. (**E**) Pile-up image of Hi-C matrices for tomato genes homologous to *Arabidopsis* single-gene domains. All other genes in tomato genome were used as control genes. Black lines indicate gene borders. (**F**) Pile-up image of Hi-C matrices for tomato adjacent gene pairs homologous to *Arabidopsis* dual-gene domains. All other adjacent gene pairs in tomato genome were used as control gene pairs. Black lines indicate the boundaries of identified adjacent gene pairs. In (**E**) and (**F**), homologous genes or gene pairs within the colinear block of tomato and *Arabidopsis* were used.

Click here to access/download;Figure;Figures.pdf

Figure 1

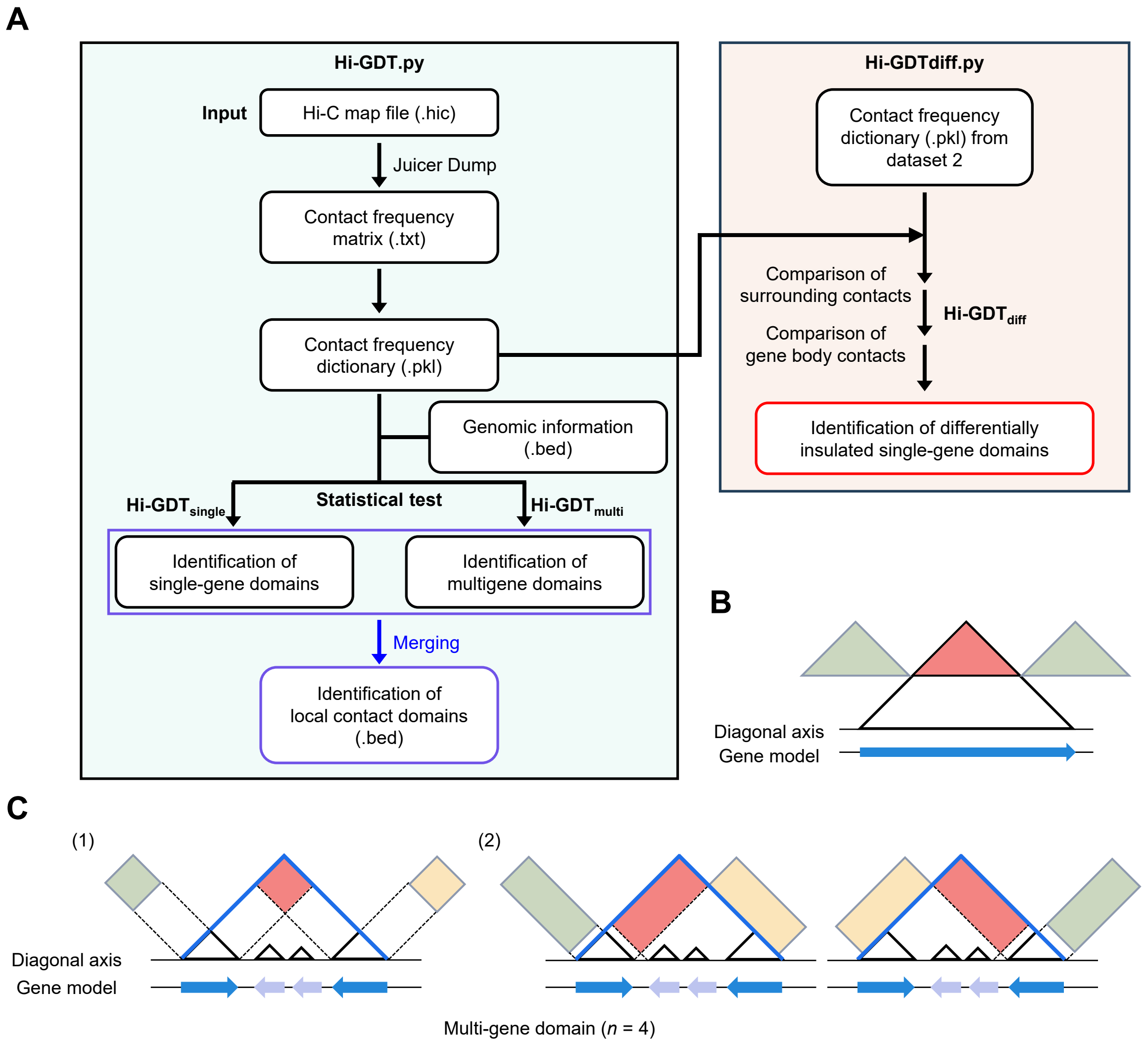

Figure 2

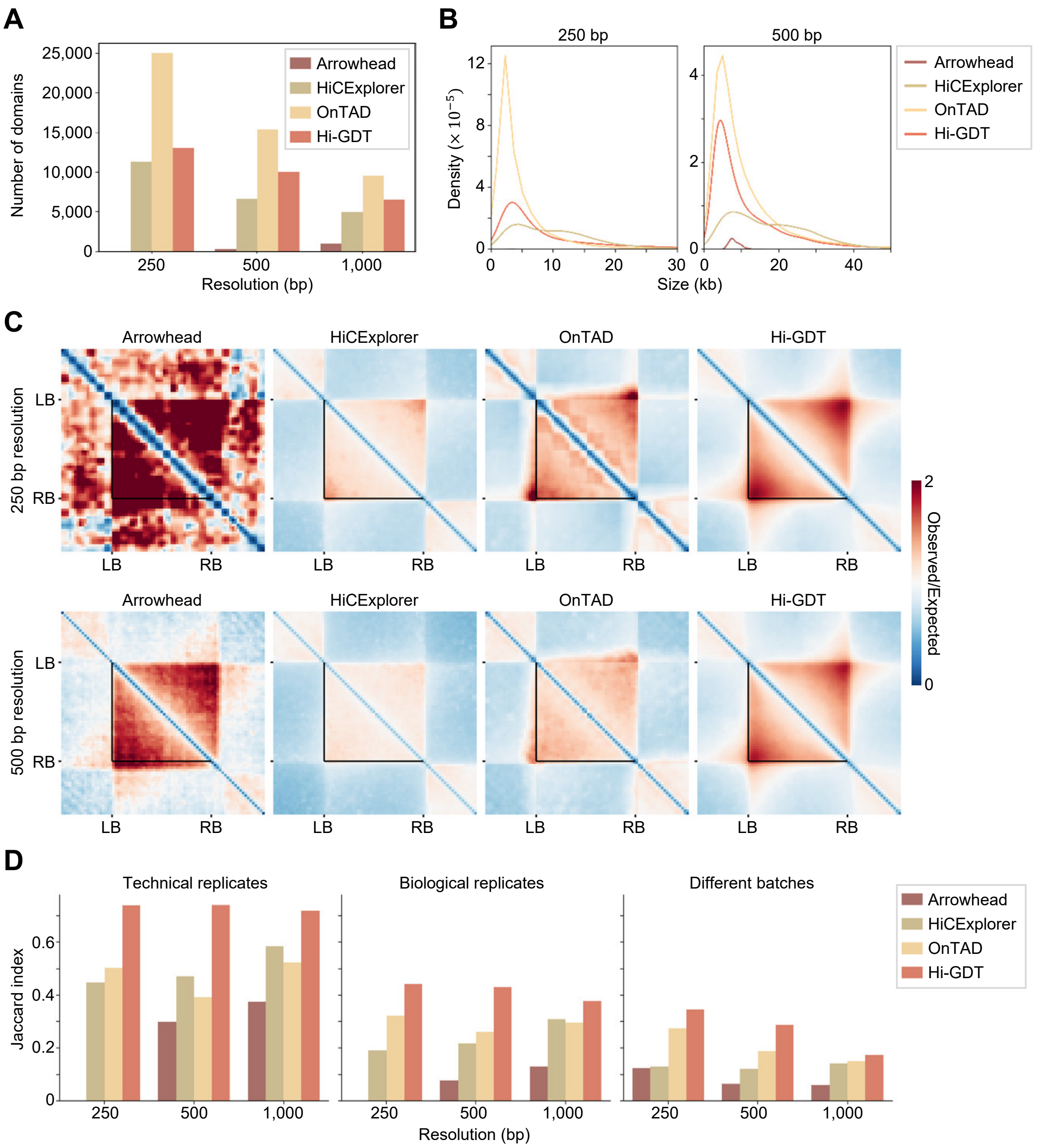

A
Number of domains
25,000
20,000
15,000
10,000
5,000
0
250
500
1,000
Resolution (bp)
Arrowhead
HiCExplorer
OnTAD
Hi-GDT
B
250 bp
500 bp
Density (× 10⁻⁵)
Size (kb)
C
Arrowhead
HiCExplorer
OnTAD
Hi-GDT
250 bp resolution
500 bp resolution
LB
RB
Observed/Expected
D
Technical replicates
Biological replicates
Different batches
Jaccard index
Resolution (bp)

Figure 3

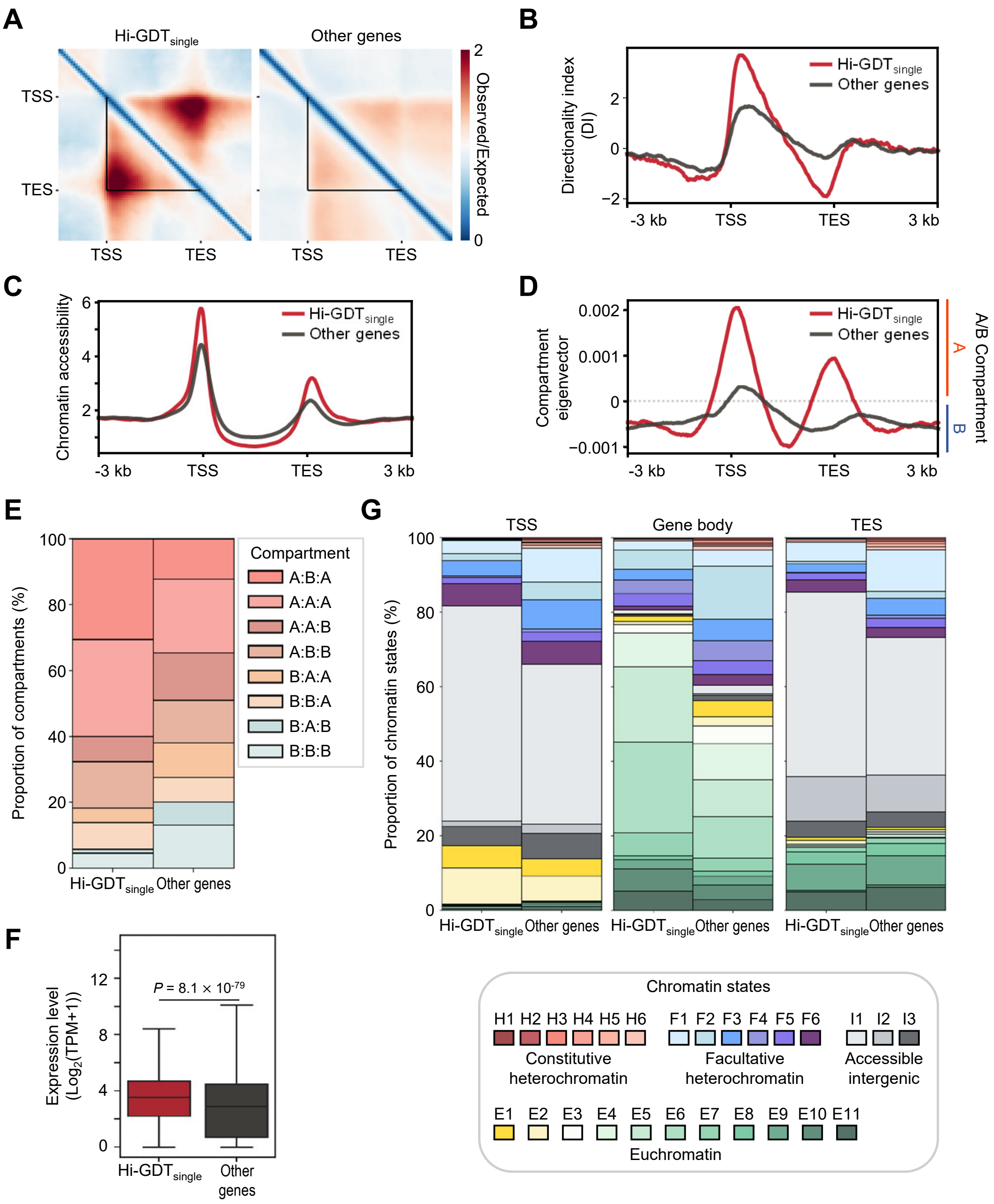

A
Hi-GDT single
Other genes
TSS
TES
2
0
Observed/Expected
B
Directionality index (DI)
Hi-GDT single
Other genes
-3 kb
TSS
TES
3 kb
C
Chromatin accessibility
D
Compartment eigenvector
0.002
0.001
0
−0.001
A/B Compartment
A
B
E
Proportion of compartments (%)
Compartment
A:B:A
A:A:A
A:A:B
A:B:B
B:A:A
B:B:A
B:A:B
B:B:B
F
Expression level (Log2(TPM+1))
P = 8.1 × 10-79
Hi-GDT single
Other genes
G
Gene body
Proportion of chromatin states (%)
Chromatin states
H1 H2 H3 H4 H5 H6
Constitutive heterochromatin
F1 F2 F3 F4 F5 F6
Facultative heterochromatin
I1 I2 I3
Accessible intergenic
E1 E2 E3 E4 E5 E6 E7 E8 E9 E10 E11
Euchromatin

Figure 4

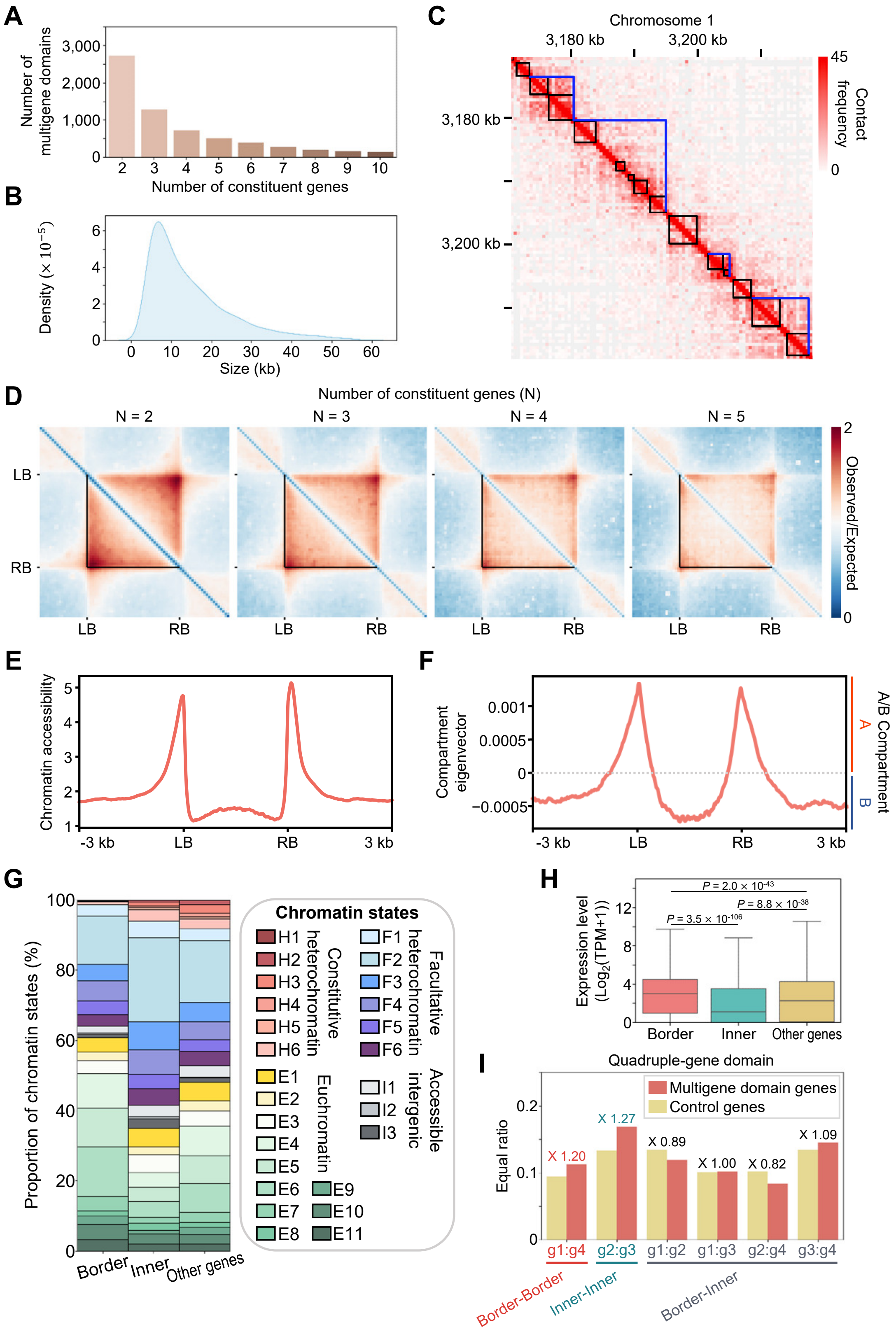
A
Number of multigene domains
3,000
2,000
1,000
0
2 3 4 5 6 7 8 9 10
Number of constituent genes
B
Density (× 10⁻⁵)
Size (kb)
C
Chromosome 1
3,180 kb
3,200 kb
Contact frequency
45
0
D
Number of constituent genes (N)
N = 2
N = 3
N = 4
N = 5
LB
RB
Observed/Expected
E
Chromatin accessibility
-3 kb
3 kb
F
Compartment eigenvector
A/B Compartment
A
B
G
Proportion of chromatin states (%)
Border
Inner
Other genes
Chromatin states
Constitutive heterochromatin
H1 H2 H3 H4 H5 H6
Facultative heterochromatin
F1 F2 F3 F4 F5 F6
Euchromatin
E1 E2 E3 E4 E5 E6 E7 E8 E9 E10 E11
Accessible intergenic
I1 I2 I3
H
Expression level (Log2(TPM+1))
P = 2.0 × 10-43
P = 8.8 × 10-38
P = 3.5 × 10-106
Border
Inner
Other genes
I
Quadruple-gene domain
Equal ratio
Multigene domain genes
Control genes
X 1.20
X 1.27
X 0.89
X 1.00
X 0.82
X 1.09
g1:g4
g2:g3
g1:g2
g1:g3
g2:g4
g3:g4
Border-Border
Inner-Inner
Border-Inner

Figure 5

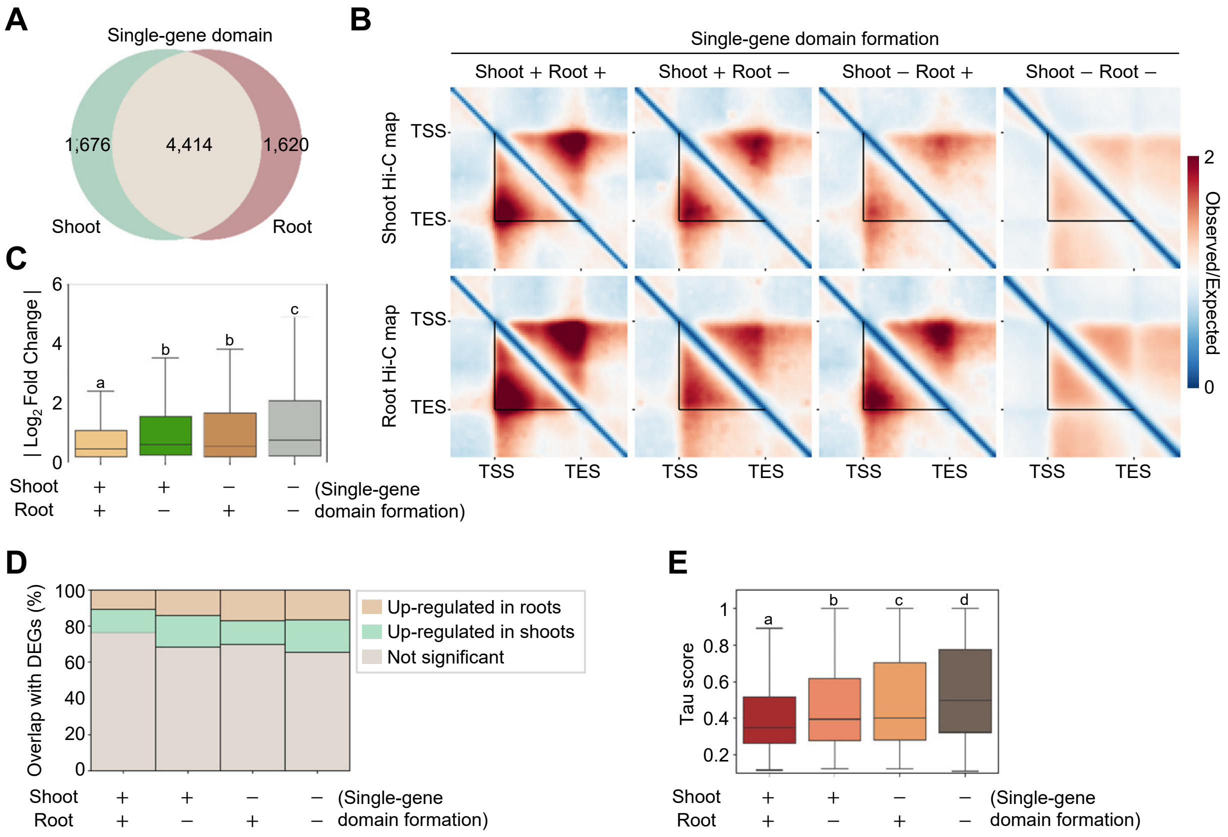
A
Single-gene domain
1,676
4,414
1,620
Shoot
Root
B
Single-gene domain formation
Shoot + Root +
Shoot + Root –
Shoot – Root +
Shoot – Root –
Shoot Hi-C map
Root Hi-C map
TSS
TES
Observed/Expected
C
| Log2 Fold Change |
a
b
b
c
Shoot + + – – (Single-gene
Root + – + – domain formation)
D
Overlap with DEGs (%)
Up-regulated in roots
Up-regulated in shoots
Not significant
Shoot + + – – (Single-gene
Root + – + – domain formation)
E
Tau score
a
b
c
d
Shoot + + – – (Single-gene
Root + – + – domain formation)

Figure 6

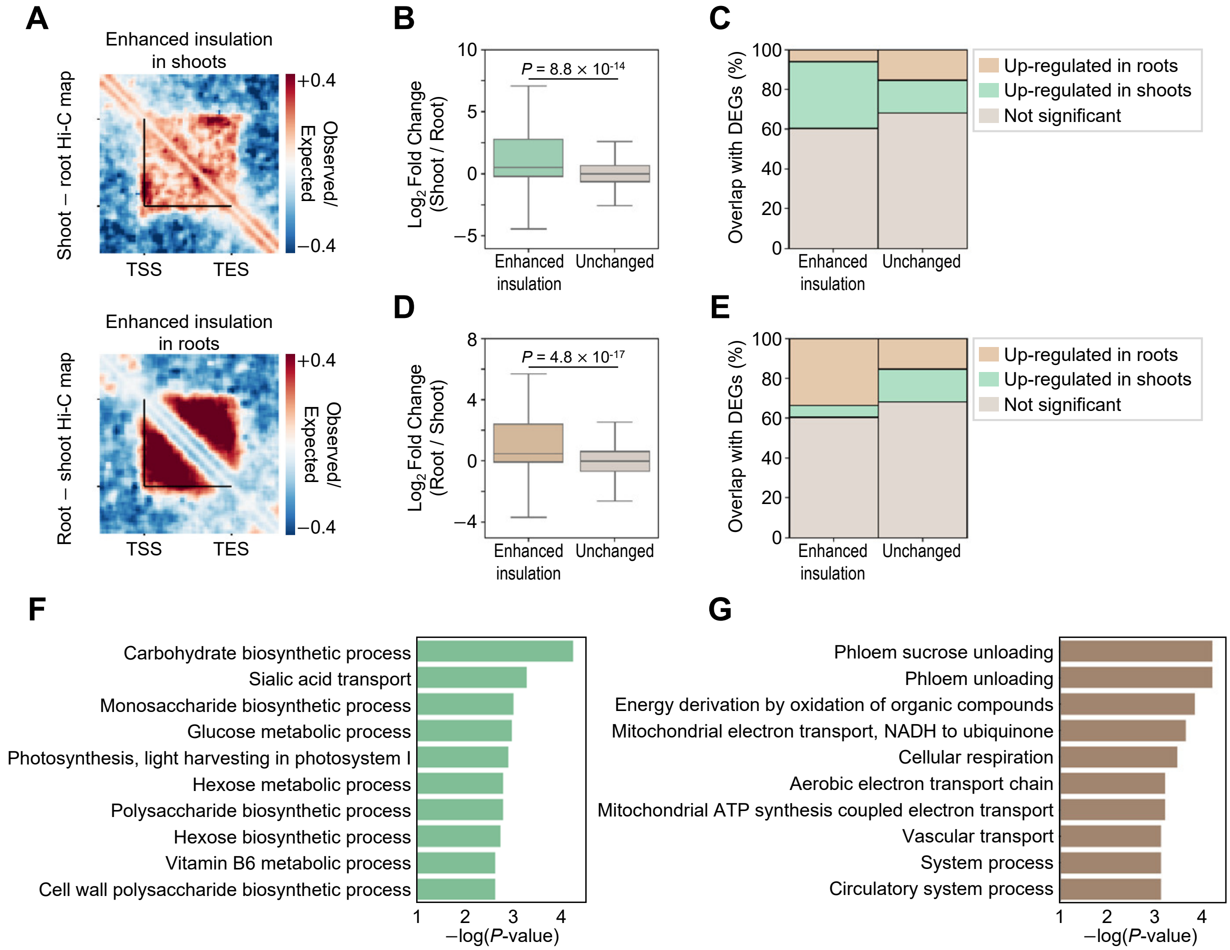
A
Enhanced insulation in shoots
Shoot – root Hi-C map
+0.4
−0.4
Observed/ Expected
TSS
TES
Enhanced insulation in roots
Root – shoot Hi-C map
B
Log2 Fold Change (Shoot / Root)
P = 8.8 × 10-14
Enhanced insulation
Unchanged
C
Overlap with DEGs (%)
Up-regulated in roots
Up-regulated in shoots
Not significant
D
Log2 Fold Change (Root / Shoot)
P = 4.8 × 10-17
E
F
Carbohydrate biosynthetic process
Sialic acid transport
Monosaccharide biosynthetic process
Glucose metabolic process
Photosynthesis, light harvesting in photosystem I
Hexose metabolic process
Polysaccharide biosynthetic process
Hexose biosynthetic process
Vitamin B6 metabolic process
Cell wall polysaccharide biosynthetic process
−log(P-value)
G
Phloem sucrose unloading
Phloem unloading
Energy derivation by oxidation of organic compounds
Mitochondrial electron transport, NADH to ubiquinone
Cellular respiration
Aerobic electron transport chain
Mitochondrial ATP synthesis coupled electron transport
Vascular transport
System process
Circulatory system process

Figure 7

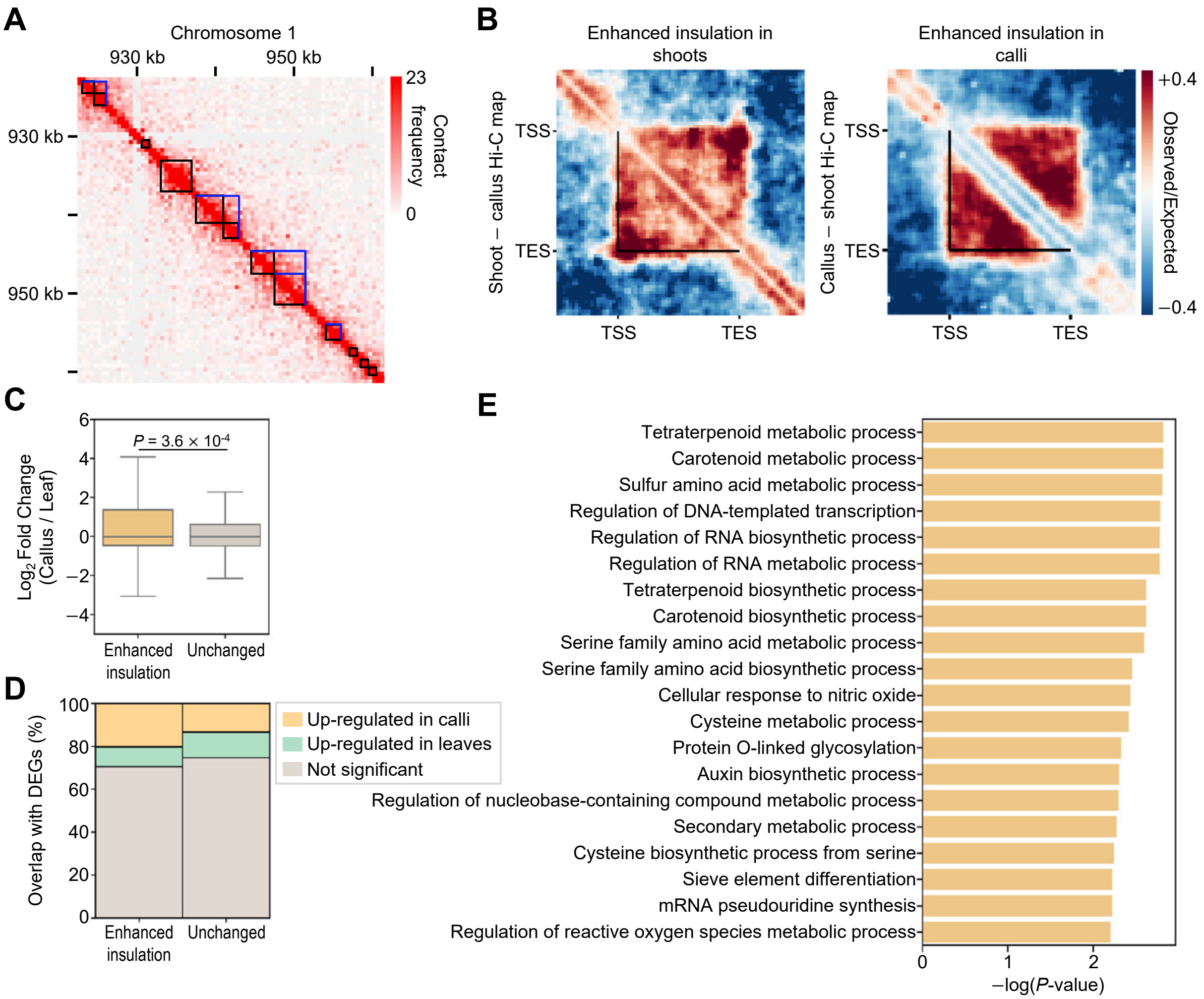
A
Chromosome 1
930 kb
950 kb
930 kb
950 kb
23
0
Contact frequency
B
Enhanced insulation in shoots
Enhanced insulation in calli
Shoot – callus Hi-C map
Callus – shoot Hi-C map
TSS
TES
+0.4
−0.4
Observed/Expected
C
P = 3.6 × 10-4
Log2 Fold Change (Callus / Leaf)
6
4
2
0
−2
−4
Enhanced insulation
Unchanged
D
Overlap with DEGs (%)
100
80
60
40
20
0
Up-regulated in calli
Up-regulated in leaves
Not significant
Enhanced insulation
Unchanged
E
Tetraterpenoid metabolic process
Carotenoid metabolic process
Sulfur amino acid metabolic process
Regulation of DNA-templated transcription
Regulation of RNA biosynthetic process
Regulation of RNA metabolic process
Tetraterpenoid biosynthetic process
Carotenoid biosynthetic process
Serine family amino acid metabolic process
Serine family amino acid biosynthetic process
Cellular response to nitric oxide
Cysteine metabolic process
Protein O-linked glycosylation
Auxin biosynthetic process
Regulation of nucleobase-containing compound metabolic process
Secondary metabolic process
Cysteine biosynthetic process from serine
Sieve element differentiation
mRNA pseudouridine synthesis
Regulation of reactive oxygen species metabolic process
0
1
2
−log(P-value)

Figure 8

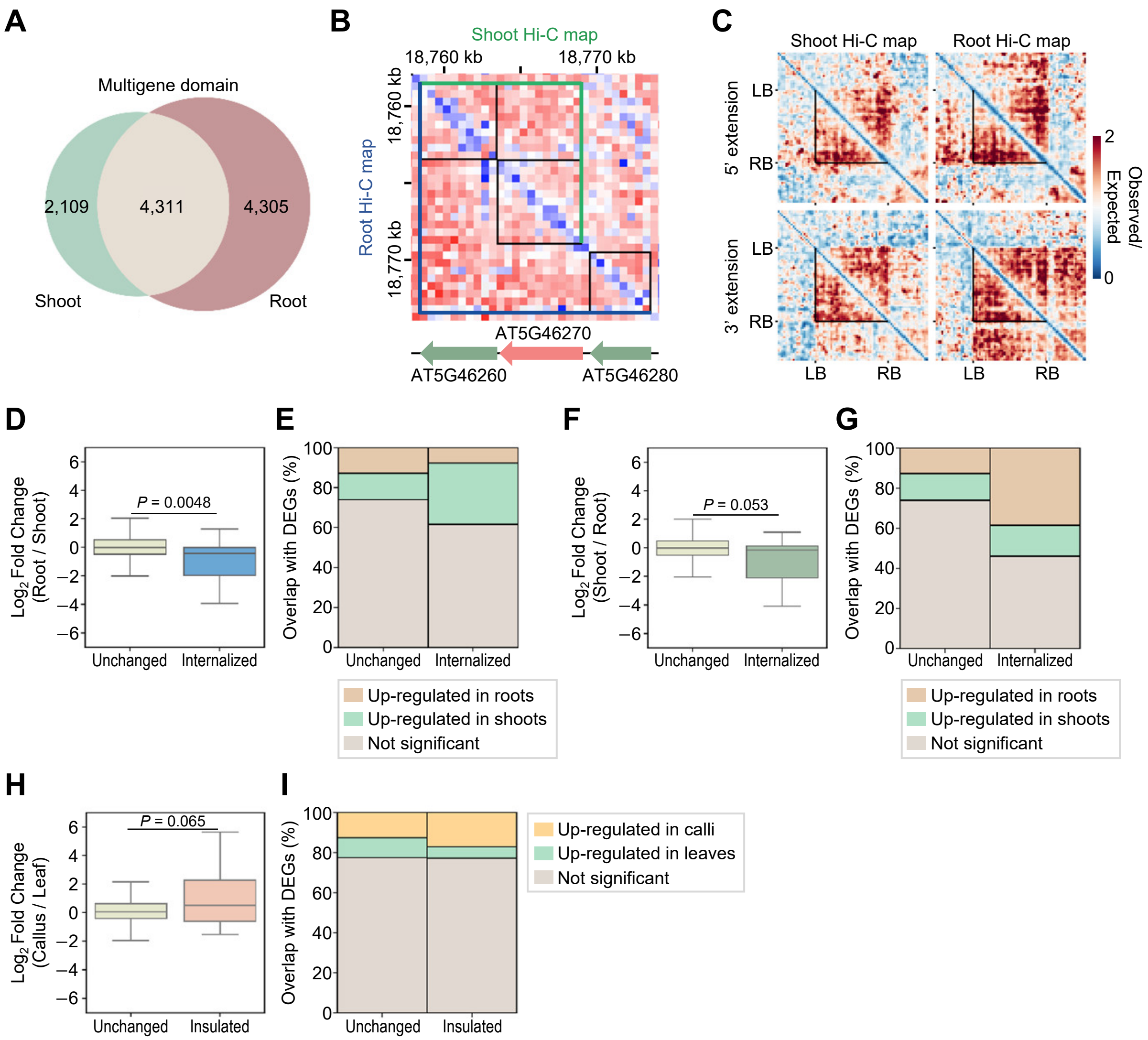

A
Multigene domain
2,109
4,311
4,305
Shoot
Root
B
Shoot Hi-C map
18,760 kb
18,770 kb
Root Hi-C map
AT5G46270
AT5G46260
AT5G46280
C
Shoot Hi-C map
Root Hi-C map
5' extension
3' extension
LB
RB
Observed/ Expected
D
Log2 Fold Change (Root / Shoot)
P = 0.0048
Unchanged
Internalized
E
Overlap with DEGs (%)
Unchanged
Internalized
Up-regulated in roots
Up-regulated in shoots
Not significant
F
Log2 Fold Change (Shoot / Root)
P = 0.053
Unchanged
Internalized
G
Overlap with DEGs (%)
Unchanged
Internalized
Up-regulated in roots
Up-regulated in shoots
Not significant
H
Log2 Fold Change (Callus / Leaf)
P = 0.065
Unchanged
Insulated
I
Overlap with DEGs (%)
Unchanged
Insulated
Up-regulated in calli
Up-regulated in leaves
Not significant

Figure 9

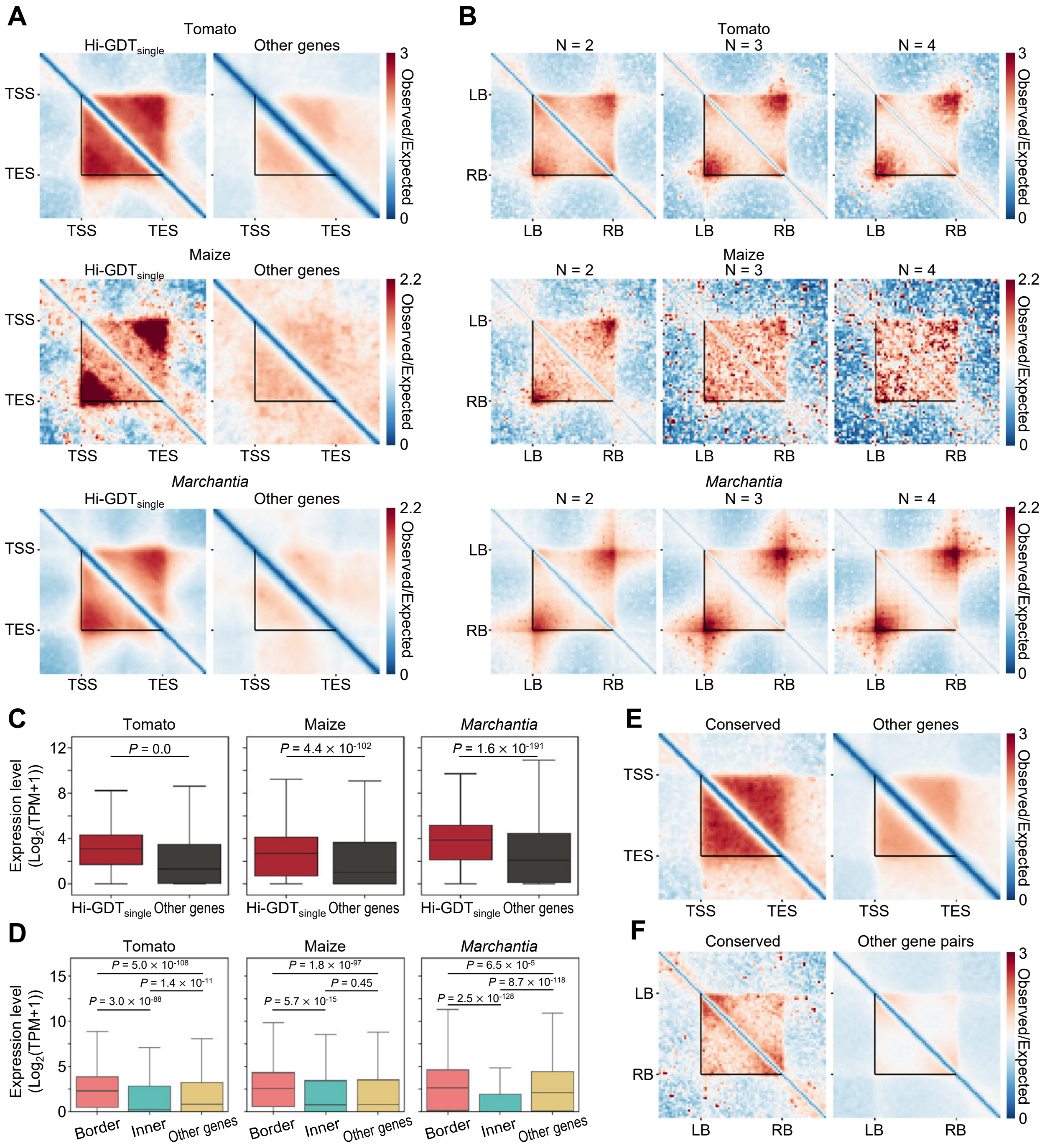

A
Tomato
Hi-GDT single
Other genes
TSS
TES
Observed/Expected
Maize
Marchantia
B
N = 2
N = 3
N = 4
LB
RB
C
Expression level (Log2(TPM+1))
P = 0.0
P = 4.4 × 10-102
P = 1.6 × 10-191
D
Border
Inner
P = 5.0 × 10-108
P = 1.4 × 10-11
P = 3.0 × 10-88
P = 1.8 × 10-97
P = 0.45
P = 5.7 × 10-15
P = 6.5 × 10-5
P = 8.7 × 10-118
P = 2.5 × 10-128
E
Conserved
F
Other gene pairs

Click here to access/download
Supplementary Material
Supplementary Figures.pdf

Supplementary Table S1

Click here to access/download
Supplementary Material
Supplementary Table S1.xlsx

Supplementary Table S2

Click here to access/download
Supplementary Material
Supplementary Table S2.xlsx

Supplementary Table S3

Click here to access/download
Supplementary Material
Supplementary Table S3.xlsx

Supplementary Table S4

Click here to access/download
Supplementary Material
Supplementary Table S4.xlsx

Supplementary Table S5

Click here to access/download
Supplementary Material
Supplementary Table S5.xlsx

Cover letter Click here to access/download;Personal Cover;Cover letter.docx

Pil Joon Seo, Ph.D.
Department of Chemistry
Seoul National University
Seoul 08826
Korea

January 6, 2025

Dr. Hongling Zhou
Editor at *GigaScience*

RE: Research article revision "Hi-GDT: A Hi-C-based 3D gene domain analysis tool for analyzing local chromatin contacts in plants" by Lee and Seo.

Dear Dr. Zhou,

Please find enclosed the revised version of the research article entitled: "Hi-GDT: A Hi-C-based 3D gene domain analysis tool for analyzing local chromatin contacts in plants" by Lee and Seo. We would like to thank you for the opportunity for revising the manuscript (GIGA-D-24-00394). After reading the reviewer´s comments, we have accordingly modified the manuscript. We believe that the changes have importantly improved the manuscript and we thank the reviewers for their invaluable comments and you, for your editorial suggestions.

The specific changes are detailed in a point-to-point response letter to the reviewers (see below). We hope that the incorporated changes make now the article suitable for publication in *GigaScience*.

Thank you very much for your consideration.

Sincerely yours,

Pil Joon Seo

Responses to Editorial Comments

Please register any new software application in the bio.tools and SciCrunch.org databases to receive RRID (Research Resource Identification Initiative ID) and biotoolsID identifiers, and include these in your manuscript. Computational workflows should be registered in workflowhub.eu and the DOIs cited in the relevant places in the manuscript. These will facilitate tracking, reproducibility and re-use of your tool.

→**(RESPONSE) We have provided relevant information for your requests.**

Responses to Reviewer 1

1. In Figure 2A, the comparison method OnTAD identifies a significantly higher number of local contact domains at different resolutions (250 bp, 500 bp, 1000 bp) than Hi-GDT. It would be helpful to include an interpretation of these differences in detection, as well as an explanation of how they might impact downstream analyses.

→ (RESPONSE) Thank you for the suggestion. Since the number of identified domains is highly dependent on the statistical cutoff of each tool, it would be appropriate to examine whether each tool can identify a sufficient number of contact domains at high resolution and the identification is reproducible. The higher number of identified domains by OnTAD (An et al., 2019) is likely derived from its specialized logic, which is optimized to detect hierarchical (nested) domain structures. As recommended, we have added relevant descriptions in the Discussion section of the revised manuscript.

In addition, we implemented OnTAD with a higher cutoff value of 0.2 (default = 0.1) to reduce the number of identified contact domains (Figure R1). However, the Jaccard index was still comparable to the previous results, suggesting that reproducibility is likely independent of the number of identified contact domains (Figure R2). We carefully concluded that Hi-GDT outperforms existing tools, including OnTAD that has intrinsically low reproducibility in identifying fine-scale contact domains.

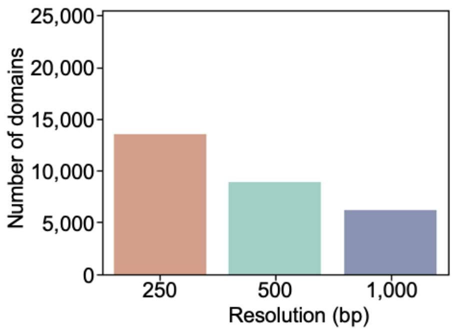

Figure R1. The number of contact domains identified by OnTAD using a cutoff value of 0.2

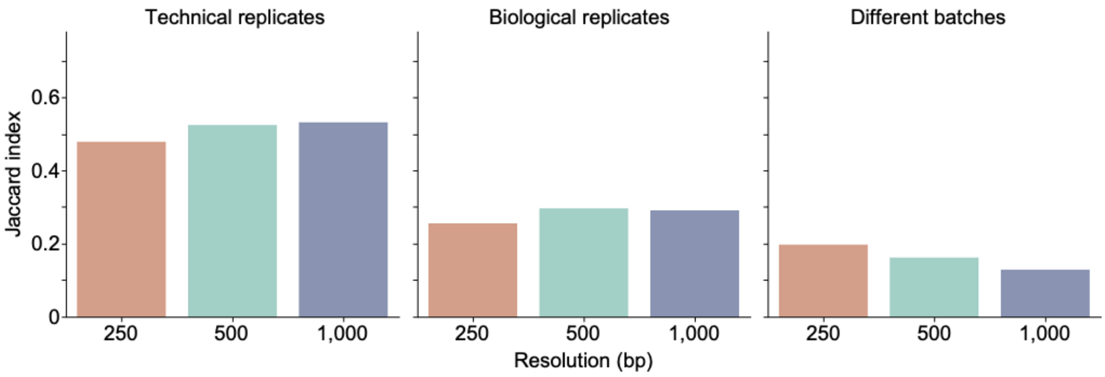

Figure R2. The Jaccard index of contact domains identified by OnTAD using a cutoff value of 0.2

2. The section on "Identification of differentially insulated single-gene domains associated with transcriptional activity depending on tissue type" notes that Hi-GDTdiff performed well on both

the wild type (Col-0) and the triple mutant (bmi1a bmi1b bmi1c). However, the manuscript does not include figures or tables to support this claim. Including visual data (e.g., charts or tables) would strengthen this section and provide clarity on the results.
→ (RESPONSE) We indicated figures with supporting results (Supplementary Figure S10 and Supplementary Table S2).

3. While Hi-GDT is shown to perform well across different plant species (e.g., Arabidopsis, tomato, maize, and Marchantia), it is unclear whether the model was trained separately for each species or designed as a generalized model. Additional details on training strategy and cross-species generalization would provide valuable insights into its adaptability and robustness.
→ (RESPONSE) Please note that we did not employ machine learning models but design rule-based algorithms. Based on our previous findings that gene domain structures are widely conserved across various plant species (Lee and Seo, 2023), we designed Hi-GDT to recognize the common feature of gene domains from Hi-C matrices, including enhanced intra-domain contacts within the boundaries of TSS and/or TES. We tried to further validate cross-species generalization by applying Hi-GDT to additional plant genomes, such as in rice, soybean, and *Jatropha curcas* (Supplementary Figure S14). We also provided additional explanations in the revised manuscript for better understanding.

4. Although the performance of Hi-GDT is notable, a detailed framework diagram of the model would enhance reader comprehension. This could clarify the structural design of Hi-GDT and facilitate reproducibility for future research.
→ (RESPONSE) We have revised Figure 1 and also added relevant information in our github page (https://github.com/CDL-HongwooLee/Hi-GDT) to improve clarity.

5. Given the growing body of research on predicting structural units and regulatory elements within three-dimensional genomes (e.g., PMID: 35997565P, PMID: 36161334, MID: 37843994, PMID: 37402191, PMID: 38485768, PMID: 39085477), it would be beneficial for the authors to reference additional recent studies in this area. Doing so would strengthen the context of the work, highlight its relevance to current advancements, and potentially broaden the article's readership.
→ (RESPONSE) We have cited relevant papers to further support our claims.

Responses to Reviewer 2

1. Through your manuscript, I noticed that there are certain differences between the three-dimensional genome organization of plants and animals. For example, you mentioned that "The CTCF core architectural protein is not conserved across various plant species…lack strong interactions at anchor sites." Additionally, your results demonstrate that your method outperforms existing methods originally developed for analyzing animal data. I suggest that you further analyze the reasons why your method is particularly well-suited for plant three-

dimensional genome analysis. Specifically, does your method explicitly account for the differences between plant and animal genome organization, which might have contributed to its superior performance?

→ (RESPONSE) The superior performance of Hi-GDT stems from addressing two major challenges (Supplemental Figure S4A): (1) the weak interactions at contact domain boundaries due to the lack of CTCFs in plants; (2) high noise in high-resolution analysis (e.g. 250 or 500 bp). The semi-targeted approach that uses gene border regions (TSS and TES) as boundaries of local contact domains (Lee and Seo, 2023; Sun et al., 2024; Zhang et al., 2024), rather than random genome-wide search, improves accuracy and sensitivity in identifying gene domains from noisy Hi-C data. We have added relevant descriptions in the Discussion section.

Lee H, Seo Pil J. Accessible gene borders establish a core structural unit for chromatin architecture in *Arabidopsis*. *Nucleic Acids Res*. 2023;51:10261-77. doi:10.1093/nar/gkad710.

Sun L, Zhou J, Xu X, et al. Mapping nucleosome-resolution chromatin organization and enhancer-promoter loops in plants using Micro-C-XL. *Nat Commun*. 2024;15:35. doi:10.1038/s41467-023-44347-z.

Zhang Y, Dong Q, Wang Z, et al. A fine-scale *Arabidopsis* chromatin landscape reveals chromatin conformation-associated transcriptional dynamics. *Nat Commun*. 2024;15:3253. doi:10.1038/s41467-024-47678-7.

2. There is a method called HiCDB(HiCDB: a sensitive and robust method for detecting contact domain boundaries) that is designed for detecting contact domains. Similar to your approach, HiCDB considers two key characteristics: the self-association and the insulation properties of contact domains. Moreover, their results demonstrate that HiCDB is effective in precisely detecting smaller-scale contact domains and have high reproducibility. Therefore, I recommend including a comparative experiment between HiCDB and your method.

→ (RESPONSE) Thank you very much for your valuable suggestion. However, we noticed that HiCDB effectively identifies the 'boundary' of local contact domains, rather than the entire contact domain structure. Our benchmark analysis evaluates several features of contact domains identified by Hi-GDT and other domain callers, including the number and size of contact domains as well as the reproducibility of contact domain identification. These analyses do not align with the output results of HiCDB, and the direct comparison is not feasible. Please understand our reasoning.

3. I have noticed a concept similar to gene domain, namely gene-body-associating domains (GADs), which can be found in the paper " tagHi-C reveals 3D chromatin architecture dynamics during mouse hematopoiesis". GADs are structurally independent of CTCF binding and highly correlated with gene expression, active histone modifications (H3K4me1), and RNA Pol II binding at the gene body. Concurrently, you have mentioned that "the concerted action of transcription factors and paused RNA polymerase II (RNA Pol II) at gene borders plays a crucial role in shaping local chromatin structure in Arabidopsis." Thus, I believe that it would be beneficial to include the distributions of RNA Pol II signals at the gene domains identified

by different methods via an aggregation plot. This addition would provide a more comprehensive demonstration of the accuracy of Hi-GDT in detecting gene domains.

→ (RESPONSE) We provided a metaplot analysis showing the RNA Pol II enrichment around the local contact domains identified by Hi-GDT or other domain callers. The data were newly included as Supplementary Figure S15.

4. It is recommended to expand the analysis and validation experiments of Hi-GDT to other publicly available datasets.

→ (RESPONSE) We applied Hi-GDT to additional plant Hi-C data including rice, soybean, and *Jatropha curcas*, and found that Hi-GDT successfully identified gene domain structures in such plant species. The data were newly included as Supplementary Figure S17.
