# Supplementary material for: Hi-GDT: A Hi-C-based 3D gene domain analysis tool for analyzing local chromatin contacts in plants
Source: Gigascience. 2025 Mar 21;14:giaf020. doi: 10.1093/gigascience/giaf020 (PMC11927400; doi:10.1093/gigascience/giaf020)
Supplement: giaf020_Response_to_Reviewer_Comments_Original_Submission [file giaf020_response_to_reviewer_comments_original_submission.pdf]

Author's Response To Reviewer Comments

Responses to Editorial Comments
Please register any new software application in the bio.tools and SciCrunch.org databases to receive RRID (Research Resource Identification Initiative ID) and biotoolsID identifiers, and include these in your manuscript. Computational workflows should be registered in workflowhub.eu and the DOIs cited in the relevant places in the manuscript. These will facilitate tracking, reproducibility and re-use of your tool.
◊(RESPONSE) We have provided relevant information for your requests.

Responses to Reviewer 1
1. In Figure 2A, the comparison method OnTAD identifies a significantly higher number of local contact domains at different resolutions (250 bp, 500 bp, 1000 bp) than Hi-GDT. It would be helpful to include an interpretation of these differences in detection, as well as an explanation of how they might impact downstream analyses.
◊ (RESPONSE) Thank you for the suggestion. Since the number of identified domains is highly dependent on the statistical cutoff of each tool, it would be appropriate to examine whether each tool can identify a sufficient number of contact domains at high resolution and the identification is reproducible. The higher number of identified domains by OnTAD (An et al., 2019) is likely derived from its specialized logic, which is optimized to detect hierarchical (nested) domain structures. As recommended, we have added relevant descriptions in the Discussion section of the revised manuscript.
In addition, we implemented OnTAD with a higher cutoff value of 0.2 (default = 0.1) to reduce the number of identified contact domains (Figure R1). However, the Jaccard index was still comparable to the previous results, suggesting that reproducibility is likely independent of the number of identified contact domains (Figure R2). We carefully concluded that Hi-GDT outperforms existing tools, including OnTAD that has intrinsically low reproducibility in identifying fine-scale contact domains.

Figure R1. The number of contact domains identified by OnTAD using a cutoff value of 0.2

Figure R2. The Jaccard index of contact domains identified by OnTAD using a cutoff value of 0.2

2. The section on "Identification of differentially insulated single-gene domains associated with transcriptional activity depending on tissue type" notes that Hi-GDTdiff performed well on both the wild type (Col-0) and the triple mutant (bmi1a bmi1b bmi1c). However, the manuscript does not include figures or tables to support this claim. Including visual data (e.g., charts or tables) would strengthen this section and provide clarity on the results.
◊ (RESPONSE) We indicated figures with supporting results (Supplementary Figure S10 and Supplementary Table S2).

3. While Hi-GDT is shown to perform well across different plant species (e.g., Arabidopsis, tomato, maize, and Marchantia), it is unclear whether the model was trained separately for each species or designed as a generalized model. Additional details on training strategy and cross-species generalization would provide valuable insights into its adaptability and robustness.
◊ (RESPONSE) Please note that we did not employ machine learning models but design rule-based algorithms. Based on our previous findings that gene domain structures are widely conserved across various plant species (Lee and Seo, 2023), we designed Hi-GDT to recognize the common feature of gene domains from Hi-C matrices, including enhanced intra-domain contacts within the boundaries of TSS and/or TES. We tried to further validate cross-species generalization by applying Hi-GDT to additional plant genomes, such as in rice, soybean, and Jatropha curcas (Supplementary Figure S14). We also provided additional explanations in the revised manuscript for better understanding.

4. Although the performance of Hi-GDT is notable, a detailed framework diagram of the model would enhance reader comprehension. This could clarify the structural design of Hi-GDT and facilitate

reproducibility for future research.
◊ (RESPONSE) We have revised Figure 1 and also added relevant information in our github page (https://github.com/CDL-HongwooLee/Hi-GDT) to improve clarity.

5. Given the growing body of research on predicting structural units and regulatory elements within three-dimensional genomes (e.g., PMID: 35997565P, PMID: 36161334, MID: 37843994, PMID: 37402191, PMID: 38485768, PMID: 39085477), it would be beneficial for the authors to reference additional recent studies in this area. Doing so would strengthen the context of the work, highlight its relevance to current advancements, and potentially broaden the article's readership.
◊ (RESPONSE) We have cited relevant papers to further support our claims.

Responses to Reviewer 2
1. Through your manuscript, I noticed that there are certain differences between the three-dimensional genome organization of plants and animals. For example, you mentioned that "The CTCF core architectural protein is not conserved across various plant species…lack strong interactions at anchor sites." Additionally, your results demonstrate that your method outperforms existing methods originally developed for analyzing animal data. I suggest that you further analyze the reasons why your method is particularly well-suited for plant three-dimensional genome analysis. Specifically, does your method explicitly account for the differences between plant and animal genome organization, which might have contributed to its superior performance?
◊ (RESPONSE) The superior performance of Hi-GDT stems from addressing two major challenges (Supplemental Figure S4A): (1) the weak interactions at contact domain boundaries due to the lack of CTCFs in plants; (2) high noise in high-resolution analysis (e.g. 250 or 500 bp). The semi-targeted approach that uses gene border regions (TSS and TES) as boundaries of local contact domains (Lee and Seo, 2023; Sun et al., 2024; Zhang et al., 2024), rather than random genome-wide search, improves accuracy and sensitivity in identifying gene domains from noisy Hi-C data. We have added relevant descriptions in the Discussion section.

Lee H, Seo Pil J. Accessible gene borders establish a core structural unit for chromatin architecture in Arabidopsis. Nucleic Acids Res. 2023;51:10261-77. doi:10.1093/nar/gkad710.
Sun L, Zhou J, Xu X, et al. Mapping nucleosome-resolution chromatin organization and enhancer-promoter loops in plants using Micro-C-XL. Nat Commun. 2024;15:35. doi:10.1038/s41467-023-44347-z.
Zhang Y, Dong Q, Wang Z, et al. A fine-scale Arabidopsis chromatin landscape reveals chromatin conformation-associated transcriptional dynamics. Nat Commun. 2024;15:3253. doi:10.1038/s41467-024-47678-7.

2. There is a method called HiCDB(HiCDB: a sensitive and robust method for detecting contact domain boundaries) that is designed for detecting contact domains. Similar to your approach, HiCDB considers two key characteristics: the self-association and the insulation properties of contact domains. Moreover, their results demonstrate that HiCDB is effective in precisely detecting smaller-scale contact domains and have high reproducibility. Therefore, I recommend including a comparative experiment between HiCDB and your method.
◊ (RESPONSE) Thank you very much for your valuable suggestion. However, we noticed that HiCDB effectively identifies the 'boundary' of local contact domains, rather than the entire contact domain structure. Our benchmark analysis evaluates several features of contact domains identified by Hi-GDT and other domain callers, including the number and size of contact domains as well as the reproducibility of contact domain identification. These analyses do not align with the output results of HiCDB, and the direct comparison is not feasible. Please understand our reasoning.

3. I have noticed a concept similar to gene domain, namely gene-body-associating domains (GADs), which can be found in the paper " tagHi-C reveals 3D chromatin architecture dynamics during mouse hematopoiesis". GADs are structurally independent of CTCF binding and highly correlated with gene expression, active histone modifications (H3K4me1), and RNA Pol II binding at the gene body. Concurrently, you have mentioned that "the concerted action of transcription factors and paused RNA polymerase II (RNA Pol II) at gene borders plays a crucial role in shaping local chromatin structure in Arabidopsis." Thus, I believe that it would be beneficial to include the distributions of RNA Pol II signals at the gene domains identified by different methods via an aggregation plot. This addition would provide a

more comprehensive demonstration of the accuracy of Hi-GDT in detecting gene domains.
◊ (RESPONSE) We provided a metaplot analysis showing the RNA Pol II enrichment around the local contact domains identified by Hi-GDT or other domain callers. The data were newly included as Supplementary Figure S15.

4. It is recommended to expand the analysis and validation experiments of Hi-GDT to other publicly available datasets.
◊ (RESPONSE) We applied Hi-GDT to additional plant Hi-C data including rice, soybean, and Jatropha curcas, and found that Hi-GDT successfully identified gene domain structures in such plant species. The data were newly included as Supplementary Figure S17.
