# Supplementary material for: Hi-GDT: A Hi-C-based 3D gene domain analysis tool for analyzing local chromatin contacts in plants
Source: Gigascience. 2025 Mar 21;14:giaf020. doi: 10.1093/gigascience/giaf020 (PMC11927400; doi:10.1093/gigascience/giaf020)
Supplement: giaf020_Reviewer_1_Report_Original_Submission — Hao Wu -- 11/9/2024 [file giaf020_reviewer_1_report_original_submission.pdf]

Reviewer Report

Title: Hi-GDT: A Hi-C-based 3D gene domain analysis tool for analyzing local chromatin contacts in plants.

Version: Original Submission **Date:** 11/9/2024

Reviewer name: Hao Wu

Reviewer Comments to Author:

The manuscript presents Hi-GDT, a tool designed for analyzing local chromatin contacts in plant cells, with demonstrated superior performance on plant cell data such as Arabidopsis thaliana when compared to traditional methods. The authors conducted extensive experiments to validate Hi-GDT's performance, and the paper provides detailed content with well-structured arguments. Below are a few suggestions for further improvement:1ã€In Figure 2A, the comparison method OnTAD identifies a significantly higher number of local contact domains at different resolutions (250 bp, 500 bp, 1000 bp) than Hi-GDT. It would be helpful to include an interpretation of these differences in detection, as well as an explanation of how they might impact downstream analyses.2ã€The section on "Identification of differentially insulated single-gene domains associated with transcriptional activity depending on tissue type" notes that Hi-GDTdiff performed well on both the wild type (Col-0) and the triple mutant (bmi1a bmi1b bmi1c). However, the manuscript does not include figures or tables to support this claim. Including visual data (e.g., charts or tables) would strengthen this section and provide clarity on the results.3ã€While Hi-GDT is shown to perform well across different plant species (e.g., Arabidopsis, tomato, maize, and Marchantia), it is unclear whether the model was trained separately for each species or designed as a generalized model. Additional details on training strategy and cross-species generalization would provide valuable insights into its adaptability and robustness.4ã€Although the performance of Hi-GDT is notable, a detailed framework diagram of the model would enhance reader comprehension. This could clarify the structural design of Hi-GDT and facilitate reproducibility for future research.5ã€Given the growing body of research on predicting structural units and regulatory elements within three-dimensional genomes (e.g., PMID: 35997565P, PMID: 36161334, MID: 37843994, PMID: 37402191, PMID: 38485768, PMID: 39085477), it would be beneficial for the authors to reference additional recent studies in this area. Doing so would strengthen the context of the work, highlight its relevance to current advancements, and potentially broaden the article's readership.

Level of Interest

Please indicate how interesting you found the manuscript: Choose an item.

Quality of Written English

Please indicate the quality of language in the manuscript: Choose an item.

Declaration of Competing Interests

Please complete a declaration of competing interests, considering the following questions:

- Have you in the past five years received reimbursements, fees, funding, or salary from an organisation that may in any way gain or lose financially from the publication of this manuscript, either now or in the future?
- Do you hold any stocks or shares in an organisation that may in any way gain or lose financially from the publication of this manuscript, either now or in the future?
- Do you hold or are you currently applying for any patents relating to the content of the manuscript?
- Have you received reimbursements, fees, funding, or salary from an organization that holds or has applied for patents relating to the content of the manuscript?
- Do you have any other financial competing interests?
- Do you have any non-financial competing interests in relation to this paper?

If you can answer no to all of the above, write 'I declare that I have no competing interests' below. If your reply is yes to any, please give details below.

N/A

I agree to the open peer review policy of the journal. I understand that my name will be included on my report to the authors and, if the manuscript is accepted for publication, my named report including any attachments I upload will be posted on the website along with the authors' responses. I agree for my report to be made available under an Open Access Creative Commons CC-BY license (http://creativecommons.org/licenses/by/4.0/). I understand that any comments which I do not wish to be included in my named report can be included as confidential comments to the editors, which will not be published.

Choose an item.

To further support our reviewers, we have joined with Publons, where you can gain additional credit to further highlight your hard work (see: https://publons.com/journal/530/gigascience). On publication of this paper, your review will be automatically added to Publons, you can then choose whether or not to claim your Publons credit. I understand this statement.

Yes Choose an item.
