# Supplementary material for: Hi-GDT: A Hi-C-based 3D gene domain analysis tool for analyzing local chromatin contacts in plants
Source: Gigascience. 2025 Mar 21;14:giaf020. doi: 10.1093/gigascience/giaf020 (PMC11927400; doi:10.1093/gigascience/giaf020)
Supplement: giaf020_Reviewer_2_Report_Original_Submission — Yusen Ye -- 11/20/2024 [file giaf020_reviewer_2_report_original_submission.pdf]

Reviewer Report

Title: Hi-GDT: A Hi-C-based 3D gene domain analysis tool for analyzing local chromatin contacts in plants.

Version: Original Submission **Date:** 11/20/2024

Reviewer name: Yusen Ye

Reviewer Comments to Author:

The study introduces Hi-GDT, a specialized tool for identifying fine-scale local chromatin contact domains in plants, emphasizing gene borders. It leverages Hi-C data to discern single-gene and multigene domains, revealing dynamic chromatin interactions linked to gene expression and tissue specificity. Hi-GDT's design focuses on plant genomes, where traditional TAD analysis tools underperform due to the lack of conserved architectural proteins like CTCF. The tool successfully identifies localized contact domains in Arabidopsis and other plants, with implications for understanding the epigenetic regulation of plant genomes.However, there are some issues that need to be addressed before considering this paper for publication. If these problems are appropriately addressed, this reviewer believes that the paper's essential contribution to TAD analysis is significant.Major 1:Through your manuscript, I noticed that there are certain differences between the three-dimensional genome organization of plants and animals. For example, you mentioned that "The CTCF core architectural protein is not conserved across various plant species…lack strong interactions at anchor sites." Additionally, your results demonstrate that your method outperforms existing methods originally developed for analyzing animal data. I suggest that you further analyze the reasons why your method is particularly well-suited for plant three-dimensional genome analysis. Specifically, does your method explicitly account for the differences between plant and animal genome organization, which might have contributed to its superior performance?Major 2:There is a method called HiCDB(HiCDB: a sensitive and robust method for detecting contact domain boundaries) that is designed for detecting contact domains. Similar to your approach, HiCDB considers two key characteristics: the self-association and the insulation properties of contact domains. Moreover, their results demonstrate that HiCDB is effective in precisely detecting smaller-scale contact domains and have high reproducibility. Therefore, I recommend including a comparative experiment between HiCDB and your method.Major 3:I have noticed a concept similar to gene domain, namely gene-body-associating domains (GADs), which can be found in the paper " tagHi-C reveals 3D chromatin architecture dynamics during mouse hematopoiesis". GADs are structurally independent of CTCF binding and highly correlated with gene expression, active histone modifications (H3K4me1), and RNA Pol II binding at the gene body. Concurrently, you have mentioned that "the concerted action of transcription factors and paused RNA polymerase II (RNA Pol II) at gene borders plays a crucial role in shaping local chromatin structure in Arabidopsis." Thus, I believe that it would be beneficial to include the distributions of RNA Pol II signals at the gene domains identified by different methods via an aggregation plot. This addition would provide a more comprehensive demonstration of the accuracy of Hi-GDT in detecting gene domains.4. It is

recommended to expand the analysis and validation experiments of Hi-GDT to other publicly available datasets.

Level of Interest

Please indicate how interesting you found the manuscript: Choose an item.

Quality of Written English

Please indicate the quality of language in the manuscript: Choose an item.

Declaration of Competing Interests

Please complete a declaration of competing interests, considering the following questions:

- Have you in the past five years received reimbursements, fees, funding, or salary from an organisation that may in any way gain or lose financially from the publication of this manuscript, either now or in the future?
- Do you hold any stocks or shares in an organisation that may in any way gain or lose financially from the publication of this manuscript, either now or in the future?
- Do you hold or are you currently applying for any patents relating to the content of the manuscript?
- Have you received reimbursements, fees, funding, or salary from an organization that holds or has applied for patents relating to the content of the manuscript?
- Do you have any other financial competing interests?
- Do you have any non-financial competing interests in relation to this paper?

If you can answer no to all of the above, write 'I declare that I have no competing interests' below. If your reply is yes to any, please give details below.

'I declare that I have no competing interests'

I agree to the open peer review policy of the journal. I understand that my name will be included on my report to the authors and, if the manuscript is accepted for publication, my named report including any attachments I upload will be posted on the website along with the authors' responses. I agree for my report to be made available under an Open Access Creative Commons CC-BY license (http://creativecommons.org/licenses/by/4.0/). I understand that any comments which I do not wish to be included in my named report can be included as confidential comments to the editors, which will not be published.

Choose an item.

To further support our reviewers, we have joined with Publons, where you can gain additional credit to further highlight your hard work (see: https://publons.com/journal/530/gigascience). On publication of this paper, your review will be automatically added to Publons, you can then choose whether or not to claim your Publons credit. I understand this statement.

Yes Choose an item.
